# Reaction of indoles with aromatic fluoromethyl ketones: an efficient synthesis of trifluoromethyl(indolyl)phenylmethanols using K_2_CO_3_/*n-*Bu_4_PBr in water

**DOI:** 10.3762/bjoc.16.71

**Published:** 2020-04-20

**Authors:** Thanigaimalai Pillaiyar, Masoud Sedaghati, Gregor Schnakenburg

**Affiliations:** 1 PharmaCenter Bonn, Pharmaceutical Institute, Department of Pharmaceutical and Medicinal Chemistry, University of Bonn, An der Immenburg 4, D-53121 Bonn, Germany; 2 Institute of Inorganic Chemistry, University of Bonn, Gerhard-Domagk-Str. 1, D-53121 Bonn, Germany

**Keywords:** C–C-bond formation, C3-funtionalization of indole, diindolylmethane, Friedel–Crafts reaction, indole, indole-3-carbinol, large-scale synthesis, recyclability

## Abstract

A new, mild and efficient protocol for the synthesis of trifluoromethyl(indolyl)phenylmethanols by the reaction of indoles with a variety of aromatic fluoromethyl ketones in the presence of K_2_CO_3_ (15 mol %) and *n-*Bu_4_PBr (15 mol %) in water. The desired products were obtained in good to excellent yields without requiring a column chromatographic purification. The reusability of the catalytic system and large-scale synthesis of indolyl(phenyl)methanols, which would further transform into biological active indole-derived compounds, are further advantages of this protocol.

## Introduction

(1*H*-Indol-3-yl)methanols have emerged as versatile pre-electrophiles for C–C functionalization at the position 3 of indoles [1–4]. Friedel–Crafts alkylation of (1*H*-indol-3-yl)methanols with indoles has proven to be a powerful strategy for the preparation of biologically important 3,3′-diindolylmethanes (DIMs) [5–14]. Additionally, (1*H*-indol-3-yl)methanols have been used as key precursors for the construction of complex indole derivatives that would be useful in pharmaceuticals as drugs and agrochemicals [2–14]. The simple (1*H*-indol-3-yl)methanol, a breakdown product of glucobrassicin, which can be found in cruciferous vegetables [15], has a wide range of biomedical applications as an anticancer [16], antioxidant, and antiatherogenic agent [17].

**Figure 1:**
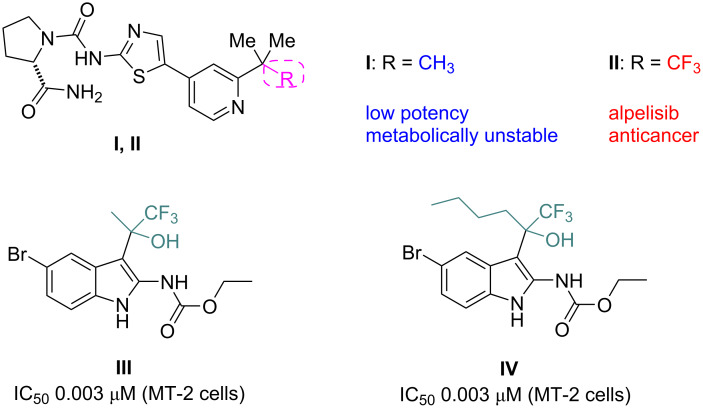
Structures of trifluoromethylated compounds and their biological activities.

Organofluorine compounds have attracted much attention due to their potential biological applications in medicinal and agricultural sciences. Introducing fluoro groups into organic molecules can dramatically influence their physiochemical and biological properties in comparison with non-fluorinated analogs [18] (see compounds **I** and **II** in Figure 1). Many pharmaceuticals and agrochemicals developed in recent decades have either a fluorine atom or a trifluoromethyl group [19–22]. Given this, the development of a method for the incorporation of fluorine or trifluoromethyl group into organic molecules perhaps remains a current challenge in organic chemistry methodology. For example, trifluoromethyl-substituted (1*H*-indol-3-yl)methanol derivatives were reported for their promising anti-HIV inhibitory activities [23], see for example compounds **III** and **IV** (Figure 1). However, these compounds were synthesized from multiple synthetic routes with the involvement of air sensitive conditions.

**Figure 2:**
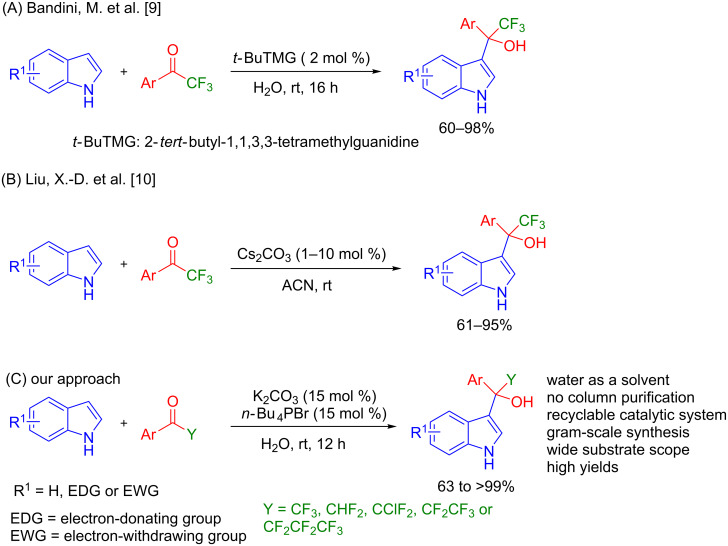
Synthetic approaches toward hydroxyalkylation of indole.

Trifluoromethyl-substituted (1*H*-indol-3-yl)methanol derivatives can be synthesized by Friedel–Crafts hydroxyalkylation reactions of indoles with trifluoromethyl ketones in the presence of either Lewis/Bronsted acid catalysts. Bandini et al. reported the trifluoromethyl hydroxyalkylation of indoles catalyzed by an organic base 2-*tert*-butyl-1,1,3,3-tetramethylguanidine (TMG), also known as Barton's base, in excellent yields (Figure 2A) [24]. Recently, Liu and co-workers reported this reaction in the presence of cesium carbonate in acetonitrile (Figure 2B) [25]. Dinuclear zinc [26], cinchonidine catalysts, and solvent-free conditions [27] have been also utilized for this reaction. The base-catalyzed reaction has a benefit because it may avoid the formation of diindolylmethane and biindoles as byproducts [28].

Although these methods were useful, they have limitations and drawbacks, which are the use of organic solvents [24], difficulties in recyclability of the catalyst [24–25], multiple reaction steps involving air sensitive/unhydrous conditions [23], generation of a large volume of waste liquids for the compound separation and column chromatographic purification [24–25], and moderate substrate scope of the reaction. Thus, finding an alternative method with broad substrate scope, functional group tolerance, and simple purification technique is highly desired.

In our continuous effort of synthesizing indole derivatives [29–31], herein, we report an efficient synthesis of multiple halogen-substituted (1*H*-indol-3-yl)methanol derivatives in the presence of potassium carbonate and tetrabutylphophonium bromide, which mediate the reaction in water through the formation of the interface between organic and aqueous phases. The advantageous of this reaction include high yields, no column chromatography, broad substrate scope, multiscale synthesis, and recyclable of the catalyst (Figure 2C).

## Results and Discussion

The optimization studies were carried out with model substrates 5-methoxyindole (**1a**, 3.4 mmol) and 2,2,2-trifluoroacetophenone (**2a**, 3.70 mmol) in water (5 mL, see Table 1 for results). Using water as a solvent has the advantage that the formed product **3a** would not be soluble, which makes the purification easier through a simple filtration. In a first attempt, the reaction did not initiate at all without any base or catalyst (Table 1, entry 1). Next, we investigated the reaction in the presence of 20 mol % of base such as NaOH (Table 1, entry 2) or KOH (Table 1, entry 3). However, no product was formed (Table 1, entries 2 and 3), and it was observerd that reactant **2a** separated from the aqueous phase. This makes us add quaternary salts into the reaction, which could make the interface between organic and aqueous phase. Very interestingly, the combination of 20 mol % of tetrabutylammonium bromide (TBAB) and NaOH (20 mol %) yielded the desired product, 2,2,2-trifluoro-1-(1*H*-indol-3-yl)ethan-1-ol (**3a**) in 93% yield (Table 1, entry 4). The product **3a** was isolated by filtration and confirmed by NMR and X-ray crystallography analysis (CCDC-1973322, see Supporting Information File 1 for detailed crystallographic data). It is noteworthy to mention that the formation of the product was even increased in the presence of tetrabutylphosphonium bromide (TBPB) to 96% (Table 1, entry 5). On the other hand, the reaction did not initiate only with TBPB (Table 1, entry 6). It indicates that quaternary salts mediate the reaction in the presence of a base through the formation of the interface between the organic and aqueous phase.

**Table 1:** Optimization of reaction conditions for the preparation of 2,2,2-trifluoro-1-(1*H*-indol-3-yl)ethan-1-ol (**3a**).^a^

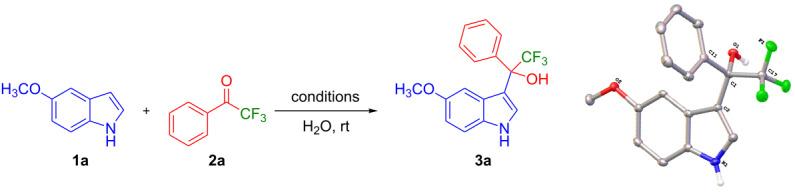				

Entry	Base (mol %)	Catalyst (mol %)	Time (h)	Yield (%)^b^

1	–	–	24	0
2	NaOH (20)	–	24	0
3	KOH (20)	–	24	0
4	NaOH (20)	TBAB (20)	15	93
5	NaOH (20)	TBPB (20)	15	96
6	–	TBPB (20)	24	0
7	KOH (20)	TBPB (20)	15	91
8	K_2_CO_3_ (20)	TBPB (20)	12	97
9	CS_2_CO_3_ (20)	TBPB (20)	15	81
10	K_3_PO_4_ (20)	TBPB (20)	15	89
11	K_2_HPO_4_ (20)	TBPB (20)	15	70
12	Na_5_P_3_O_10_ (20)	TBPB (20)	15	76
13	K_2_CO_3_ (20)	TBAC (20)	15	72
14	K_2_CO_3_ (20)	TBAF (20)	15	61
**15**	**K_2_CO_3 _(15)**	**TBPB (15)**	**12**	**>99** (**94**)^c^
16	K_2_CO_3_ (10)	TBPB (10)	18	90
17	K_2_CO_3_ (5)	TBPB (5)	24	69

^a^Reaction conditions: **1a** (500 mg, 3.4 mmol) and **2a** (524 µL, 3.70 mmol) were used in H_2_O (5 mL) at room temperature. ^b^Isolated yields. ^c^Obtained yield in tap water.

As a next step, different bases were investigated by using TBPB. In the presence of KOH and the Cs_2_CO_3_, the formation of the product was reduced to 91% (Table 1, entry 7) and 81% (Table 1, entry 9), respectively, while it was increased to 97% (Table 1, entry 8) in the presence of K_2_CO_3_. The reaction in the presence of tripotassium phosphate base (Table 1, entry 10; 89%), dipotassium phosphate base (Table 1, entry 11; 70%) or sodium triphosphate (Table 1, entry 12; 76%) did not improve the yield. These results suggest that K_2_CO_3_ is the best catalyst for the reaction among the bases investigated.

To find the best catalytic system, other quaternary salts were invested in the reaction. When tetrabutylammonium chloride (TBAC) or tetrabutylammonium fluoride (TBAF) was employed in the presence of K_2_CO_3_, the formation of the product was reduced to 72% (Table 1, entry 13) and 61% (Table 1, entry 14), respectively. These results suggest that the combination of K_2_CO_3_/TBPB could be the best catalytic system for this reaction.

Next, the amount of catalytic system K_2_CO_3_/TBPB was reduced from 20 mol % to 15 mol % to see if there any change in the yield. As indicated in Table 1, entry 15, the product **3a** was isolated in >99%. However, further reduction to 10 mol % or 5 mol % slowed the reaction rate, and only 90% (Table 1, entry 16) or 69% (Table 1, entry 17) of **3a** was isolated. These results suggest that the combination of K_2_CO_3_ (15 mol %)/*n-*Bu_4_PBr (15 mol %) is a suitable and efficient catalytic system for this reaction.

Having the optimized conditions in hand, the substrate scope of the reaction was explored. At first, different trifluoromethyl ketones were investigated, and the results are summarized in Table 2. Trifluoroacetophenones having a halogen substituent at the *para*-position of the phenyl ring such as *p*-F (**2b**), *p*-Cl (**2c**), and *p*-Br (**2d**) provided the corresponding trifluoro-1-(1*H*-indol-3-yl)ethan-1-ols **3b**, **3c**, and **3d** in 97, 92 and 89% yields, respectively. Similarly, trifluoroacetophenones having a *p*-methyl (**2e**) and *p*-methoxy (**2f**) group at the phenyl ring resulted in **3e**, and **3f** with excellent yields of 98 and 93%, respectively. These results suggest that the electronic properties of the substituent on the phenyl ring of the trifluoroacetophenones did not significantly influence the yield of the reaction.

**Table 2:** Substrate scope of the reaction with ketones.^a^

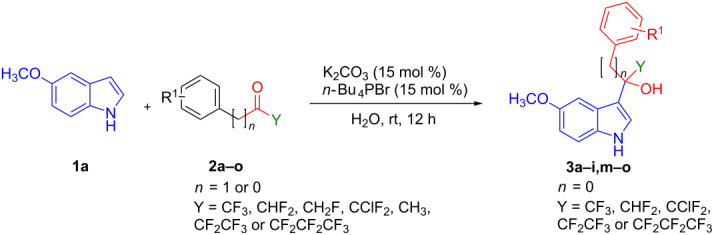				

Ketones	Multihalogen-substituted hydroxylakylated indoles	Mp (°C)	Purity (%)^b^	Yield (%)^c^

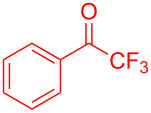 **2a**	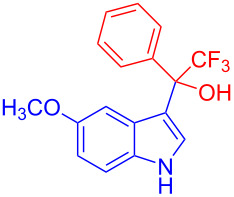 **3a** [24]	135–136 (135)^d^ [24]	99	>99% (98)^e^ [24]
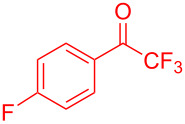 **2b**	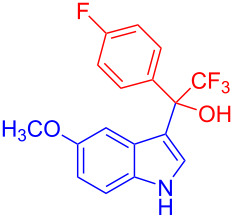 **3b**	115–116	98	97%
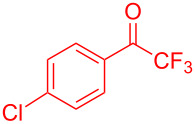 **2c**	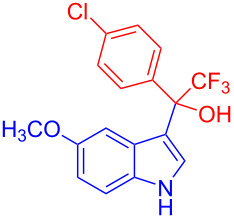 **3c**	153–154	99	92
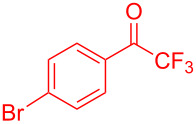 **2d**	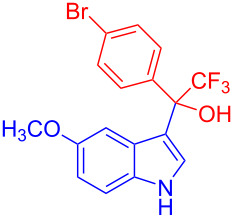 **3d**	172–173	97	89
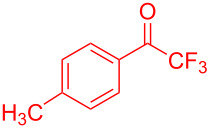 **2e**	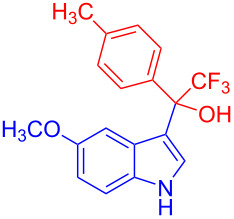 **3e**	130–131	99	98
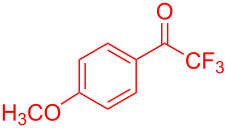 **2f**	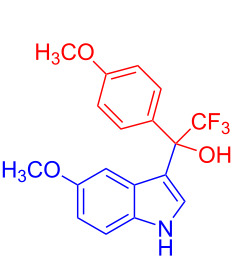 **3f**	151–152	98	93
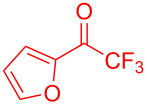 **2g**	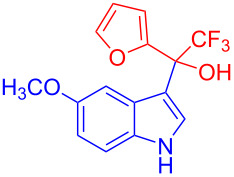 **3g**	158–159	99	97
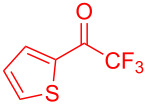 **2h**	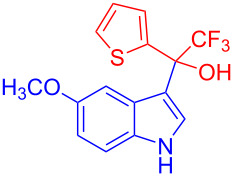 **3h**	148–149	97	98
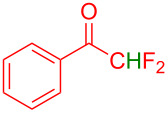 **2i**	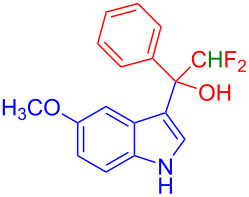 **3i**	185–186	96	63
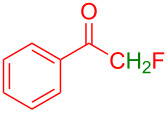 **2j**	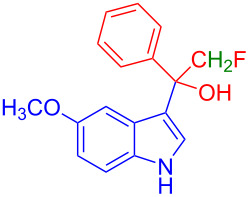 **3j**	–	–	0
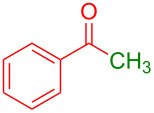 **2k**	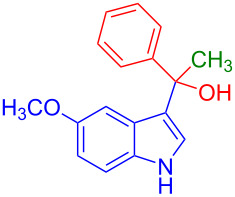 **3k**	–	–	0
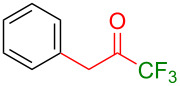 **2l**	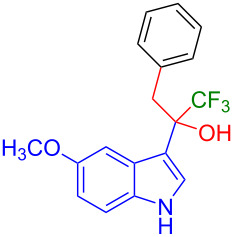 **3l**	–	–	0
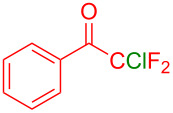 **2m**	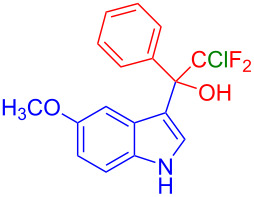 **3m**	158–159	98	94
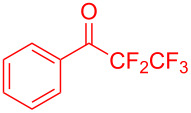 **2n**	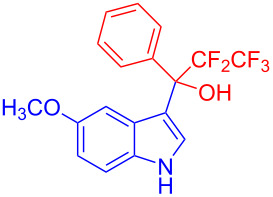 **3n**	168–169	97	93
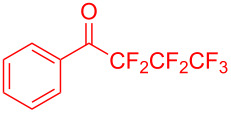 **2o**	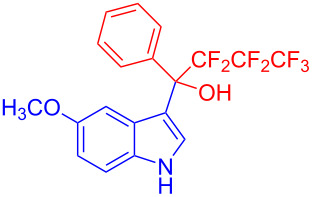 **3o**	161–163	97	90

^a^Reaction conditions: **1** (3.4 mmol) and **2** (3.75 mmol) in water (5 mL). ^b^Purity was determined by HPLC coupled to a UV diode array detector (DAD) at 220−400 nm. ^c^Isolated yields. ^d^Reported melting points. ^e^Reported yields.

The trifluoromethyl ketones having electron-rich heteroaromatics such as 2-(trifluoroacetyl)furan (**2g**) and 2-(trifluoroacetyl)thiophene (**2h**) gave the desired products **3g** (97%) and **3h** (98%) in excellent yields.

Next, the role of fluorine in the reactants was explored by subjecting the reaction of 2,2-difluoro-1-phenylethan-1-one (**2i**) with **1a**. The desired product **3i** was obtained in 63% yield. However, no product was formed when the reaction was carried out with 2-fluoro-1-phenylethan-1-one (**2j**) or acetophenone (**2k**). The reason could be due to either reducing the electrophilicity of the ketone or the presence of enolizable protons in α-position to the keto group in basic medium. Supporting this hypothesis, the reaction of **1a** with 1,1,1-trifluoro-3-phenyl-2-propanone (**2l**), which has a 3-methylene group also did not proceed at all.

The scope of ketones was further extended with mixed halogen-substituted, pentafluoro or heptafluoro ketones such as 2-chloro-2,2-difluoro-1-phenylethan-1-one (**2m**), 2,2,3,3,3-pentafluoro-1-phenylpropan-1-one (**2n**) and 2,2,3,3,4,4,4-heptafluoro-1-phenylbutan-1-one (**2o**). All these reactions provided the corresponding products (**3m**: 94%, **3n**: 93%, **3o**: 90%) in excellent yields.

Next, the scope of substituted indoles was studied with trifluoromethyl ketone **2a**, and the results are shown in Table 3. In the case of a simple indole (**1b**), the corresponding product **3p** was isolated in 96% yield. Indoles bearing electron-donating groups (i.e., methoxy, **1c**,**d**) and electron-withdrawing groups (i.e., F, **1e**,**f**) at different positions of indoles were well tolerated and delivered the desired fluorinated indol-3-yl-1-phenylethanols (**3q**–**t**) in excellent yields in the range from 79–96%. The reaction of **3a** with different azaindoles (4-. 5-, 6-, and 7-azaindoles, **1g**–**j**) provided the corresponding products in the range from 91–97% yield (**3u**–**x**).

**Table 3:** Substrate scope of the reaction with indoles.^a^

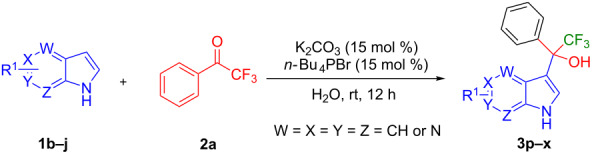				

Indoles	Trifluoromethly substituted hydroxylakylated indoles	Mp (°C)	Purity (%)^b^	Yield (%)^c^

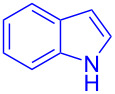 **1b**	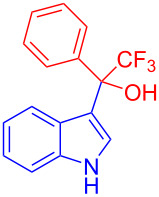 **3p**	122–124 (75)^d^ [25]	98	96 (95)^e^ [25]
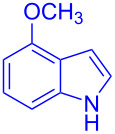 **1c**	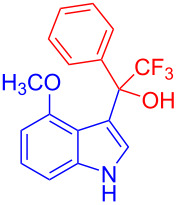 **3q**	160–161	99	79
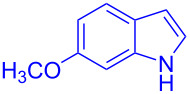 **1d**	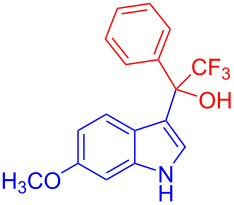 **3r**	192–193	97	90
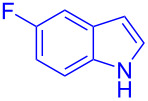 **1e**	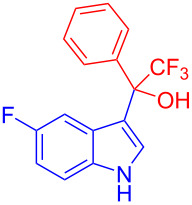 **3s**	112–113	99	94 (98)^e^ [24]
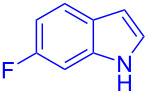 **1f**	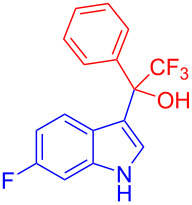 **3t**	90–91 (90)^d^ [24]	99	96 (98)^e^ [24]
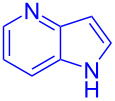 **1g**	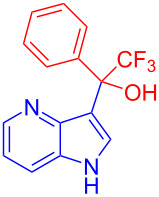 **3u**	165–166	98	91
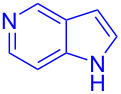 **1h**	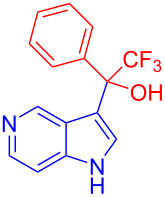 **3v**	227–228	97	92
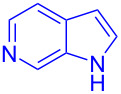 **1i**	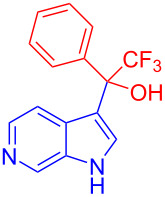 **3w**	239–240	99	97
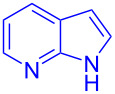 **1j**	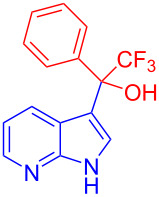 **3x**	185–186 185^d^ [24]	97	90 86^e^ [24]

^a^Reaction conditions: **1** (3.4 mmol) and **2** (3.75 mmol) in water (5 mL). ^b^Purity was determined by HPLC coupled to a UV diode array detector (DAD) at 220−400 nm. ^c^Isolated yields. ^d^Reported melting points. ^e^Reported yields.

**Figure 3:**
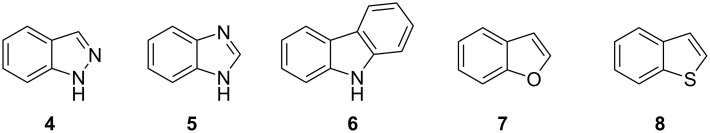
Structures of heterocycles that did not react with ketone **2a**.

We applied the developed protocol to reactions of other heterocyclic systems such as indazole (**4**), benzimidazole (**5**), carbazole (**6**), benzofuran (**7**), and benzothiophene (**8**) with ketone **2a** (Figure 3). However, no desired products are formed.

**Scheme 1:**
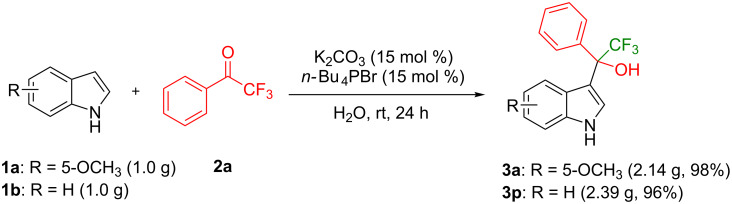
Gram-scale synthesis of 2,2,2-trifluoro-1-(1*H*-indol-3-yl)-1-phenylethan-1-ols (**3a** and **3p**).

As a next step, this protocol is employed in the large-scale preparation of 2,2,2-trifluoro-1-(1*H*-indol-3-yl)-1-phenylethan-1-ols. We performed gram-scale reactions of 5-methoxyindole (**1a**, 6.8 mmol) with 2,2,2-trifluoroacetophenone (**2a**, 7.5 mmol) or of indole (**1b**, 8.5 mmol) with **2a** (9.4 mmol, Scheme 1). In both reactions, the desired products are achieved in excellent yields (**3a**: 2.14 g, 98%; **3b**: 2.39 g, 96%).

**Figure 4:**
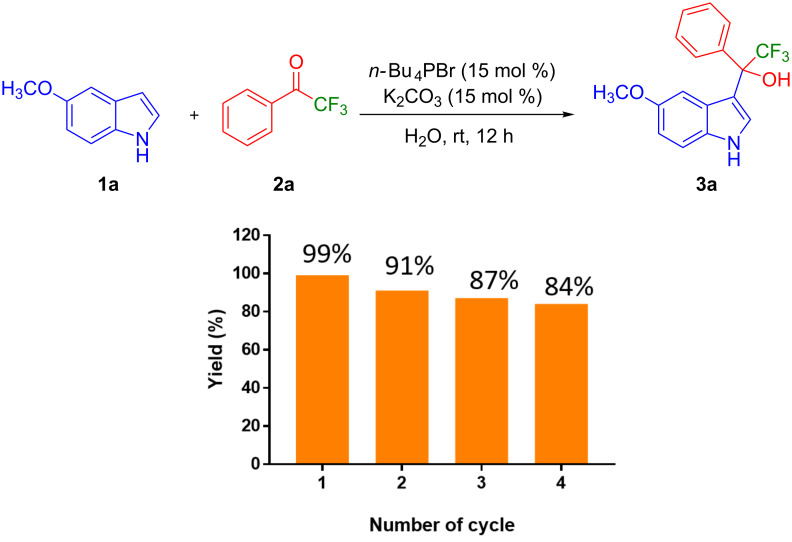
Recyclability of the catalytic system *n-*Bu_4_PBr/K_2_CO_3_ for the preparation of 2,2,2-trifluoro-1-(5-methoxy-1*H*-indol-3-yl)-1-phenylethan-1-ol (**3a**).

The recyclability of the catalytic system *n-*Bu_4_PBr/K_2_CO_3_ of this protocol was investigated in the preparation of **3a** using **1a** (3.40 mmol), **2a** (3.70 mmol), K_2_CO_3_ (0.5 mmol) and *n-*Bu_4_PBr (0.5 mmol) in distilled water (5 mL) at room temperature for 12 h (Figure 4). The product **3a** was filtered after completion of the reaction. The resulting filtrate was recovered, washed with ethyl acetate to remove any organic impurities, and reused for the next cycle. This procedure was followed for each cycle. Interestingly, the catalytic system was efficient to produce the product **3a** in excellent to good yields up to 4 cycles (99–84%). In the fifth cycle, the yield of **3a** was reduced to 67%. This could be due to the dilution of the catalytic system in each cycle.

**Scheme 2:**
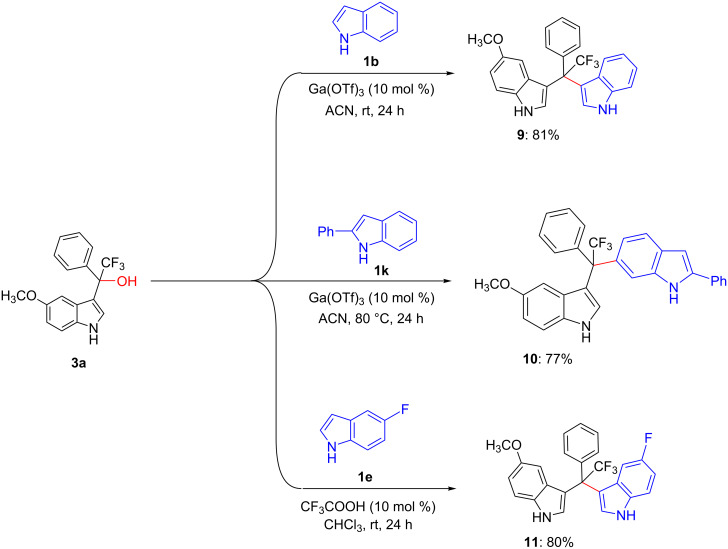
Synthesis of trifluoromethylated unsymmetrical 3,3'- and 3,6'-DIMs (**9–11**).

3-Indolylmethanols are versatile pre-electrophiles for C–C functionalization at the 3-position of indoles. Particularly, the Friedel–Crafts alkylation of 3-indolylmethanols with indoles has become a useful method for the preparation of 3,3'-, and 3,6'-DIMs, which are known to possess a wide variety of biological activities, including anti-inflammatory, and anticancer effects. Therefore, we decided to synthesize DIMs from **3a**, which reacted with indole (**1b**) or 2-phenylindole (**1k**) in the presence of Ga(OTf)_3_ in ACN at room temperature or at 80 °C as reported by Y. Ling et al. [32]. As indicated in Scheme 2, the desired unsymmetrical 3,3'-DIM (**9**: 81%) and 3,6'-DIM (**10**: 77%) with quaternary center were afforded in good yields. Besides, the protocol reported by S. Sasaki et al. [33] was also employed for the synthesis of 3,3'-DIMs in good yield, see for example product **11** (80%, Scheme 2).

**Scheme 3:**
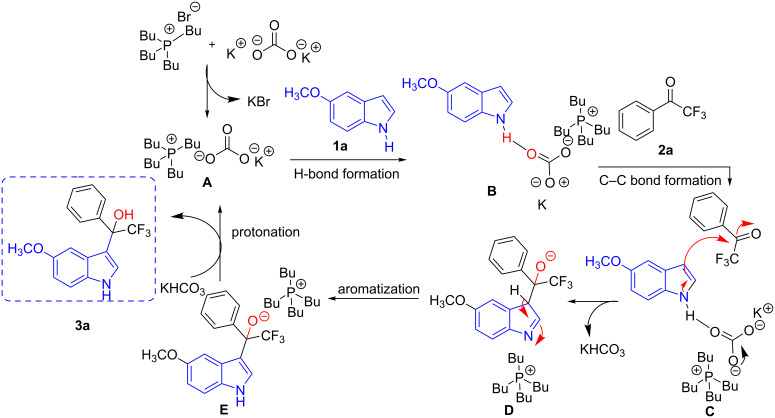
Proposed mechanism for the preparation of **3a** as an example.

Next, a plausible mechanism for the preparation of multihalogen-alkylated 1-(1*H*-indol-3-yl)-1-phenylethan-1-ols is proposed as indicated in Scheme 3. Based on the literature [34], this reaction initiates by the formation of *n-*Bu_4_P^+^KCO_3_^−^ salt (**A**) from the interaction of *n-*Bu4PBr and K_2_CO_3_. Intermediate **A** makes a hydrogen bond interaction with the NH of the 5-methoxyindole (**1a**) and form the adduct **B**. This interaction assists **1a** reacting with an electrophilic ketone (**2a**) to form the intermediate **D** via C–C bond formation (**C**). Re-aromatization of **D** generates **E**, which then protonates to form the desired product **3a** by excluding **A** for the next catalytic cycle.

**Scheme 4:**
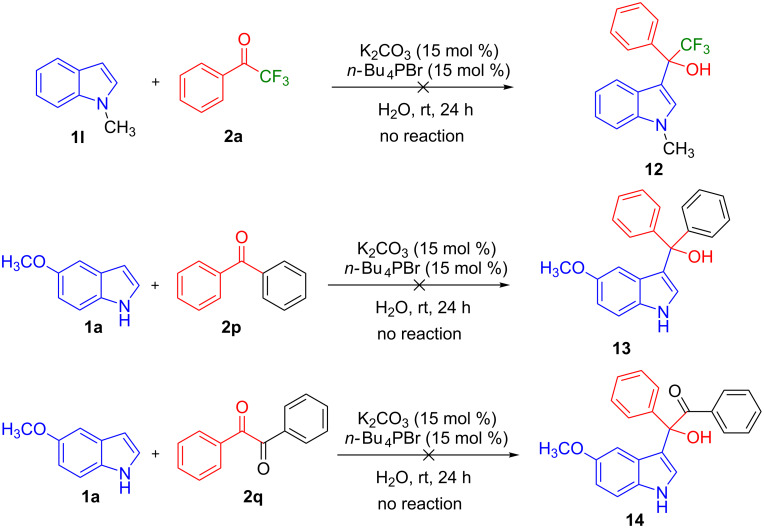
Control experiments.

Further, to prove this hypothesis the following control experiments were performed. The reaction of 1-methylindole (**1l**) with **2a** failed to provide the product (Scheme 4). It suggests that the indole having a free NH-functionality is important to interact with the base, thereby initiating the reaction. To find the importance of electrophilicity of ketones, different enolizable and nonenolizable ketones were screened with the reaction of 5-methoxyindole (**1a**). The enolizable ketones **2j**–**l** failed to provide the products (see Table 2). Similarly, nonenolizable ketones **2p** and **2q** (Scheme 4) failed to provide the products. This observation suggests that the multihalogen-substitution enhanced the electrophilicity of the ketone for the Friedel–Crafts hydroxyalkylation reaction of indole.

## Conclusion

In conclusion, we have developed an efficient and practical protocol for the preparation of trifluoromethyl(indolyl)phenylmethanols, which are of significant interest serving as pre-electrophiles for C–C functionalization at the 3-position of indoles. Particularly, the Friedel–Crafts alkylation of 3-indolylmethanols with indoles has become a useful method for the preparation of 3,3'-, and 3,6'-DIMs, which are known to possess a wide variety of biological activities, including anti-inflammatory, and anticancer effects. Additionally, trifluoromethyl(indolyl)phenylmethanols itselft have various biological properties including anti-HIV activity. The developed new synthetic protocol for the preparation of trifluoromethyl(indolyl)phenylmethanols is operationally simple and provided products in high yields without requiring silica gel column chromatography. The reaction has a broad substrate scope and proceeds with high regioselectivity. The recovery and reusability of the catalytic system and large-scale synthesis of products, which would further transform into biologically active indole-derived compounds, are further advantages of this protocol.

## Supporting Information

Materials and methods and detailed synthetic procedures and spectroscopic data of all compounds. Figure S1: ORTEP-type plot of the molecular structure of **3a**, Figures S2–S25: NMR spectra, Tables S1–S3: Crystal data and structure refinement for compound **3a**.

File 1 Experimental and analytical data.
