# Supplementary material for: Reaction of indoles with aromatic fluoromethyl ketones: an efficient synthesis of trifluoromethyl(indolyl)phenylmethanols using K2CO3/n-Bu4PBr in water
Source: Beilstein J Org Chem. 2020 Apr 20;16:778–90. doi: 10.3762/bjoc.16.71 (PMC7189012; doi:10.3762/bjoc.16.71)
Supplement: File 1 — Experimental and analytical data. [file Beilstein_J_Org_Chem-16-778-s001.pdf]

BEILSTEIN JOURNAL OF ORGANIC CHEMISTRY

Supporting Information

for

Reaction of indoles with aromatic fluoromethyl ketones: an efficient synthesis of trifluoromethyl(indolyl)phenylmethanols using K_2CO_3/*n*-Bu_4PBr in water

Thanigaimalai Pillaiyar, Masoud Sedaghati and Gregor Schnakenburg

Beilstein J. Org. Chem. **2020,** *16,* 778–790. doi:10.3762/bjoc.16.71

Experimental and analytical data

License and Terms: This is a supporting information file under the terms of the Creative Commons Attribution License (http://creativecommons.org/licenses/by/4.0). Please note that the reuse, redistribution and reproduction in particular requires that the authors and source are credited.
The license is subject to the *Beilstein Journal of Organic Chemistry* terms and conditions: (https://www.beilstein-journals.org/bjoc)

**Entry No:**	**Table of Contents**	**Page**
1	Materials and methods: general information	S2
2	General procedures for the synthesis of **3a**–**l**, **3m**–**o**	S3
3	Procedure for the synthesis of **9**	S3
4	Procedure for the synthesis of **10**	S3
5	Procedure for the synthesis of **11**	S4
6	Spectrochemical data	S4–S17
7	X-ray crystal structure determination	S18
8	**Figure S1**. ORTEP-type plot of the molecular structure of **3a**	S19
9	**Table S1.** Crystal data and structure refinement for **3a**	S20
10	**Table S2.** Bond lengths for **3a**	S21
11	**Table S3**. Bond angles for **3a**	S22
12	**Figures S2–S25**. ^{1}H and ^{13}C NMR spectrum of compounds	S23–S46
13	References	S47

Materials and methods

Experimental Section

General information: Chemicals were purchased from Merck (Darmstadt, Germany), ABCR (Karlsruhe, Germany), or TCI (Eschborn, Germany). Thin layer chromatography (TLC) was performed on TLC plates F254 (Merck) and analyzed using UV light. An LCMS instrument coupled to electrospray ionization mass spectrometry (LCESI-MS) determined the purities of isolated products using the following procedure: the compounds were dissolved at a concentration of 1.0 mg/mL in acetonitrile, containing 2 mM NH_4CH_3COO. Then, 10 µL of the sample was injected into an HPLC column (Phenomenex Luna 3 µ C18, 50 × 2.00 mm). Elution was performed with a gradient of water: methanol (containing 2 mM NH_4CH_3COO) from 90:10 to 0:100 starting the gradient immediately at a flow rate of 250 µL/min for 15 min followed by washing with 100% methanol for another 15 min. UV absorption was detected from 200 to 600 nm using a diode array detector (DAD). The purity of the compounds was determined at 220−400 nm and was ≥95% for all products. ^{1}H, ^{13}C and ^{19}F NMR data were measured in $CDCl_3$ or DMSO-d_6 as a solvent. Chemical shifts are reported in parts per million (ppm) relative to the deuterated solvents (DMSO-d_6), ^{1}H: 2.49 ppm, ^{13}C: 39.70 ppm; ($CDCl_3$) ^{1}H: 7.25 ppm, ^{13}C: 77.17 ppm; coupling constants *J* are given in Hertz and spin multiplicities are given as s (singlet), d (doublet), t (triplet), q (quartet), sext (sextet), m (multiplet), br (broad). HRMS was recorded on a micrOTOF-Q mass spectrometer (Bruker) with ESI-source coupled with an HPLC Dionex Ultimate 3000 (Thermo Scientific) using an EC 50/2 Nucleodur C18 Gravity 3 µm column (MachereyNagel). The column temperature was 425 °C. Ca. 1 µL of a 1 mg/mL solution of the sample in acetonitrile was injected and a flow rate of 0.3 mL/min was used. HPLC was started with a solution of acetonitrile in water (10:90), containing 2 mM CH_3COONH_4. The gradient was started after 1 min reaching 100% acetonitrile within 9 min and then flushed with this concentration for another 5 min. Melting points were measured on a melting point apparatus (BÜCHI melting point B-545) and are uncorrected.

General procedures for the Synthesis of 3a–I, 3m–o

The solution of 5-methoxyindole (**1a**, 3.4 mmol) and 2,2,2-trifluoro-1-phenylethan-1-one (**2a**, 3.70 mmol) was prepared in water (5 mL) and allowed it to stir at room temperature. To the solution, K_2CO_3 (0.5 mmol) and *n*-Bu_4PBr (0.5 mmol) were added. Initially, the mixture was allowed to stir vigorously due to the formation product in sticky mass. After keep stirring for a long time, the sticky mass was turned to be solid, which can be filtered through glass filter (pore size 5, 50 mL) and washed with 5% ethyl acetate in petroleum ether (boiling in the range 35–60 °C). The product was dried at 40 °C in a heating oven for further spectroscopic and physical characterizations.

Procedure for the synthesis of 9

To a solution of 2,2,2-trifluoro-1-(5-methoxy-1*H*-indol-3-yl)-1-phenylethan-1-ol (**3a**, (0.6 mmol) and indole (**1b**, 0.71 mmol) in CH_3CN (5 mL) was added $Ga(OTf)_3$ (0.06 mmol). The mixture was allowed to stir at room temperature for 24 h. The reaction mixture was evaporated under reduced pressure to dryness and resulting residue was purified by silica-gel column chromatography to give the desired product.

Procedure for the synthesis of 10

To a solution of 2,2,2-trifluoro-1-(5-methoxy-1*H*-indol-3-yl)-1-phenylethan-1-ol (**3a**, (0.6 mmol) and 2-phenylindole (**1k**, 0.71 mmol) in CH_3CN (5 mL) was added $Ga(OTf)_3$ (0.06 mmol). The mixture was allowed to stir at 80 °C for 24 h. The reaction mixture was evaporated under reduced pressure to dryness and resulting residue was purified by silica-gel column chromatography to give the desired product.

Procedure for the synthesis of 11

To a solution of 2,2,2-trifluoro-1-(5-methoxy-1*H*-indol-3-yl)-1-phenylethan-1-ol (**3a**, 0.6 mmol) in $CHCl_3$ (2 mL) were added 5-fluoroindole (**1e**, 0.75 mmol) and trifluoroacetic acid (10 mol %) at rt. The reaction mixture was stirred until the disappearance of alcohol derivatives as observed by TLC. The reaction mixture was quenched with an aqueous saturated $NaHCO_3$ solution 5 mL) and the organic layer was separated. The aqueous layer was extracted with chloroform (3 × 20 mL). The combined organic layer was dried with anhydrous Na_2SO_4 and then evaporated. The residue was purified by column chromatography.

2,2,2-Trifluoro-1-(5-methoxy-1*H*-indol-3-yl)-1-phenylethan-1-ol (3a)[1]

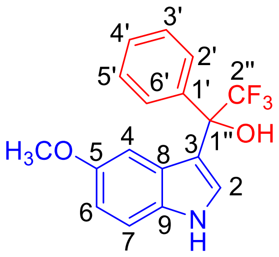

5-Methoxyindole (**1a**, 3.4 mmol), 2,2,2-trifluoro-1-phenylethan-1-one (**2a**, 3.70 mmol), K_2CO_3 (15 mol %), and *n*-Bu_4PBr (15 mol %) were used for this reaction in water (5 mL). Orange solid; yield: >99% (1.10 g); mp = 135-136 °C (135).[1] ^1H NMR (600 MHz, DMSO-d_6) δ 11.06 (d, J = 2.9 Hz, 1H, NH), 7.51 (d, J = 7.4 Hz, 2H, 3'-H, 6'-H), 7.40 – 7.29 (m, 4H, 2'-H, 4'-H, 5'-H), 7.26 (d, J = 8.7 Hz, 1H, 7-H), 6.91 (d, J = 0.8 Hz, 1H, 7-H), 6.68 (dd, J = 8.8, 2.4 Hz, 1H, 6-H), 6.41 (d, J = 2.6 Hz, 1H, 4-H), 3.46 (s, 3H, OCH_3). ^{13}C NMR (151 MHz, DMSO-d_6) δ 152.88 (5-C), 139.68 (1'-C), 131.70 (8-C), 128.04 (9-C), 127.81 (3'-C), 127.49 (2-C), 126.2 (q, J = 288.4 Hz, 2"-C), 126.1 (5'-C), 124.3 (2'-C, 4'-C), 113.3 (7-C), 112.6 (6-C), 111.6 (3-C), 103.3 (4-C), 76.2 (q, J = 28.5 Hz, 1"-C), 55.20 (O-CH_3). ^{19}F NMR (565 MHz, DMSO-d_6) δ -75.05 (CF_3). LC−MS (m/z) positive mode 322 $[M + H]^{1+}$. Purity by HPLC-UV (254 nm)-ESI-MS 99%. HRMS (ESI-QTOF) calculated for $C_{17}H_{14}F_3NO_2$ $[M + H]^+$: 322.1055; found: 322.1059.

2,2,2-Trifluoro-1-(4-fluorophenyl)-1-(5-methoxy-1*H*-indol-3-yl)ethan-1-ol (3b)

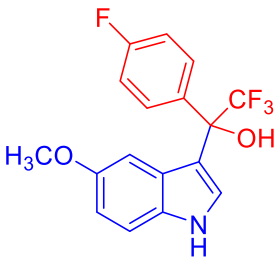

5-Methoxyindole (**1a**, 3.4 mmol), 2,2,2-trifluoro-1-(4-fluorophenyl)ethan-1-one (**2b**, 3.70 mmol), K_2CO_3 (15 mol %) and *n*-Bu_4PBr (15 mol %) were used for this reaction in water (5 mL). White crystals; yield: 97 % (1.11g); mp = 115-116 °C. ^1H NMR (600 MHz, DMSO-d_6) δ 11.12 (m, 1H, NH), 7.54 – 7.48 (m, 2'-H, 6'-H), 7.38 (t, *J* = 2.0 Hz, 1H, 4-H), 7.27 (d, *J* = 8.8 Hz, 1H, 7-H), 7.20 – 7.13 (m, 2H, 3'-H, 5'-H), 7.02 (s, 1H, 2-H), 6.70 (dd, *J* = 8.8, 2.4 Hz, 1H), 6.41 (d, *J* = 2.4 Hz, 1H, 6-H), 3.49 (s, 3H, OCH_3). ^{13}C NMR (151 MHz, DMSO-d_6) δ 161.76 (d, *J* = 244.3 Hz, 4'-C), 152.99 (5-C), 135.89 (1'-C), 131.74 (8-C), 129.77 (9-C), 125.94 (q, *J* = 289.5 Hz, 2''-C), 124.27 (2-C), 114.73 (2'-C), 114.58 (6'-C), 112. 67 (3-C), 112.38 (7-C), 111.34 (6-C), 102.82 (4-C), 75.35 (q, *J* = 29.9 Hz, 1''-C), 55.25 (OCH_3). ^{19}F NMR (565 MHz, DMSO-d_6) δ -75.34 (CF_3), -114.79, -114.80, -114.81, -114.82, -114.84 (PhF). LC−MS (m/z) positive mode 340 $[M + H]^{1+}$. Purity by HPLC-UV (254 nm)-ESI-MS 98%. HRMS (ESI-QTOF) calculated for $C_{17}H_{13}F_4NO_2$ $[M + H]^+$: 340.0961; found: 340.0970.

1-(4-Chlorophenyl)-2,2,2-trifluoro-1-(5-methoxy-1*H*-indol-3-yl)ethan-1-ol (3c)

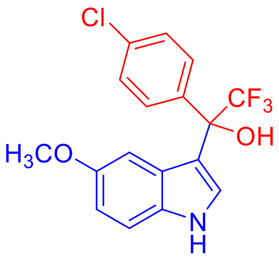

5-Methoxyindole (**1a**, 3.4 mmol), 1-(4-chlorophenyl)-2,2,2-trifluoroethan-1-one (**2c**, 3.70 mmol), K_2CO_3 (15 mol %) and *n*-Bu_4PBr (15 mol %) were used for this reaction in water (5 mL). Light brown crystals; yield: 92 % (1.10 g); mp = 153-154 °C. ^1H NMR (600 MHz, DMSO-d_6) δ 11.22 – 10.89 (s, 1H, NH), 7.53 – 7.46 (m, 2H, 3'-H, 5'-H), 7.45 – 7.39 (m, 2H, 2'-H, 4'-H), 7.39 – 7.35 (m, 2H, 2'-H, 6'-H), 7.27 (d, *J* = 8.8 Hz, 1H, 6-H), 7.07 (s, 1H, 4-H), 6.70 (dd, *J* = 8.8, 2.5 Hz, 1H, 7-H), 6.41 (d, *J* = 2.5 Hz, 1H, 2-H), 3.50 (s, 3H). ^{13}C NMR (151 MHz, DMSO-d_6) δ 153.01 (5-C), 138.72 (1'-C), 132.96 (4'-C), 131.74 (8-C), 129.48 (9-C), 127.92 (3'-C, 5'-C), 125.88 (q, *J* = 285.4 Hz, 2''-C), 124.32 (2'-C, 6'-C), 112.41 (3-C, 6-C),

102.79 (3-C), 75.5 (q, J = 29.1 Hz, 1''-C), 55.26 (OCH_3). ^{19}F NMR (565 MHz, DMSO-d_6) δ -75.31 (CF_3). LC−MS (m/z) positive mode 356 [M + H]$^{1+}$. Purity by HPLC-UV (254 nm)-ESI-MS 99%. HRMS (ESI-QTOF) calculated for $C_{17}H_{13}ClF_3NO_2$ [M + H]$^+$: 356.0665; found: 356.0667.

1-(4-Bromophenyl)-2,2,2-trifluoro-1-(5-methoxy-1*H*-indol-3-yl)ethan-1-ol (3d)

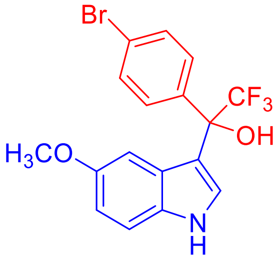

5-Methoxyindole (**1a**, 3.4 mmol), 1-(4-bromophenyl)-2,2,2-trifluoroethan-1-one (**2d**, 3.70 mmol), K_2CO_3 (15 mol %) and *n*-Bu_4PBr (15 mol %) were used for this reaction in water (5 mL). White crystals; yield: 89% (1.20 g); mp = 172-173 °C. ^1H NMR (600 MHz, DMSO-d_6) δ 11.33 (s, 1H, NH), 7.67 – 7.49 (m, 2H, 3'-H, 5'-H), 7.43 (d, J = 8.4 Hz, 2H, 2'-H, 6'-H), 7.37 (s, 1H, 2-H), 7.27 (d, J = 8.8 Hz, 1H, 6-H), 7.07 (s, 1H, 4-H), 6.70 (dd, J = 8.7, 2.5 Hz, 1H, 7-H), 6.41 (d, J = 2.5 Hz, 1H, 6-H), 3.50 (s, 3H, OCH_3). ^{13}C NMR (151 MHz, DMSO-d_6) δ 152.96 (5-C), 139.13 (1'-C), 131.69 (4'-C), 130.82 (8-C, 9-C), 129.76 (3'-C, 5'-C), 125.83 (q, J = 291.2 Hz, 2''-C), 124.27 (2'-C), 121.59 (2-C), 112.37 (3-C, 6-C), 111.25 (7-C), 102.76 (4-C), 75.5 (q, J = 28.0 Hz, 1''-C), 55.22 (OCH_3). ^{19}F NMR (565 MHz, DMSO-d_6) δ -75.27 (CF_3). LC−MS (m/z) positive mode 401 [M + H]$^{1+}$. Purity by HPLC-UV (254 nm)-ESI-MS 97%. HRMS (ESI-QTOF) calculated for $C_{17}H_{13}BrF_3NO_2$ [M + H]$^+$: 400.0160; found: 400.0153.

2,2,2-Trifluoro-1-(5-methoxy-1*H*-indol-3-yl)-1-(*p*-tolyl)ethan-1-ol (3e)

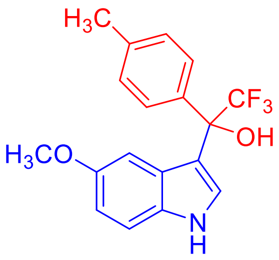

5-Methoxyindole (**1a**, 3.4 mmol), 2,2,2-trifluoro-1-(*p*-tolyl)ethan-1-one (**2e**, 3.70 mmol), K_2CO_3 (15 mol %) and *n*-Bu_4PBr (15 mol %) were used for this reaction in water (5 mL). White crystals; yield: 98% (1.11 g); mp = 130-131 °C. ^1H NMR (600 MHz, DMSO-d_6) δ 11.11 (s, 1H, NH), 7.49 – 7.30 (m, 3H, 2'-H, 6'-H, 2-H), 7.25 (d, J = 8.8 Hz, 1H, 2-H), 7.17 – 7.03 (m,

2H, 3'-H, 5'-H), 6.83 (s, 1H, 4-H), 6.68 (dd, *J* = 8.8, 2.5 Hz, 1H, 7-H), 6.45 (d, *J* = 2.4 Hz, 1H, 6-H), 3.48 (s, 3H, OCH_3), 2.28 (s, 3H, CH_3). ^{13}C NMR (151 MHz, DMSO-d_6) δ 152.84 (5-C), 137.21 (1'-C), 136.75 (4'-C), 131.71 (2-C), 128.38 (8-C, 9-C), 127.40 (3'-C, 5'-C), 126.10 (q, *J* = 286.4 Hz, 2"-C), 124.17 (2'-C, 6'-C), 113.14 (3-C), 112.20 (6-C), 111.09 (7-C), 103.20 (4-C), 75.37 (q, *J* = 28.6 Hz, 1"-C), 55.24 (OCH_3). ^{19}F NMR (565 MHz, DMSO-d_6) δ, -75.35 (CF_3). LC−MS (m/z) positive mode 336 $[M + H]^{1+}$. Purity by HPLC-UV (254 nm)-ESI-MS 99%. HRMS (ESI-QTOF) calculated for $C_{18}H_{16}F_3NO_2$ $[M + H]^+$: 336.1211; found: 336.1210.

2,2,2-Trifluoro-1-(5-methoxy-1*H*-indol-3-yl)-1-(4-methoxyphenyl)ethan-1-ol (3f)

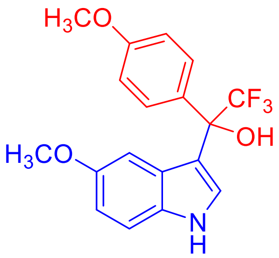

5-Methoxyindole (**1a**, 3.4 mmol), 22,2,2-trifluoro-1-(4-methoxyphenyl)ethan-1-one (**2f**, 3.70 mmol), K_2CO_3 (15 mol %) and *n*-Bu_4PBr (15 mol %) were used for this reaction in water (5 mL). White solid; yield: 93% (1.12 g); mp = 151-152 °C. ^{1}H NMR (600 MHz, DMSO-d_6) δ 11.22 (s, 1H, NH), 7.46 – 7.35 (m, 2H, 2'-H, 6'-H), 7.33 (q, *J* = 1.9 Hz, 1H, 2-H), 7.26 (dd, *J* = 8.9, 0.6 Hz, 1H, 7-H), 6.95 – 6.84 (m, 2H, 3'-H, 5'-H), 6.80 (s, 1H, 4-H), 6.69 (dd, *J* = 8.7, 2.5 Hz, 1H, 6-H), 3.73 (s, 3H, OCH_3), 3.49 (s, 3H, OCH_3). ^{13}C NMR (151 MHz, DMSO-d_6) δ 159.00 (5-C), 152.86 (5'-C), 131.73 (4'-C), 131.60 (1'-C), 128.78 (8-C, 9-C), 126.12 (q, *J* = 287.2 Hz, 2"-C), 124.16 (2'-C, 6'-C), 113.19 (2-C), 113.15 (3-C), 112.21 (6-C), 111.11 (7-C), 103.22 (4-C), 75.37 (q, *J* = 28.5 Hz, 1"-C), 55.27 (OCH_3), 55.18 (OCH_3). ^{19}F NMR (565 MHz, DMSO-d_6) δ -75.33 (CF_3). LC−MS (m/z) positive mode 352 $[M + H]^{1+}$. Purity by HPLC-UV (254 nm)-ESI-MS 98%. HRMS (ESI-QTOF) calculated for $C_{18}H_{16}F_3NO_3$ $[M + H]^+$: 352.1161; found: 352.1155.

2,2,2-Trifluoro-1-(furan-2-yl)-1-(5-methoxy-1*H*-indol-3-yl)ethan-1-ol (3g)

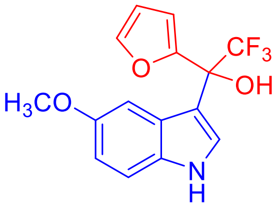

5-Methoxyindole (**1a**, 3.4 mmol), 2,2,2-trifluoro-1-(furan-2-yl)ethan-1-one (**2g**, 3.70 mmol), K_2CO_3 (15 mol %) and *n*-Bu_4PBr (15 mol %) were used for this reaction in water (5 mL). White solid; yield: 97% (1.0 g); mp = 158-159 °C. ^1H NMR (600 MHz, DMSO-*d₆*) δ 11.09 (s, 1H, NH), 7.68 (dd, *J* = 1.8, 0.9 Hz, 1H, 5'-H), 7.38 – 7.20 (m, 2H, 3'-H, 4'-H), 7.13 (s, 1H, 2-H), 6.72 (dd, *J* = 8.8, 2.5 Hz, 1H, 7-H), 6.65 (d, *J* = 2.5 Hz, 1H, 4-H), 6.59 – 6.54 (m, 1H, 6-H), 6.52 (dd, *J* = 3.3, 1.8 Hz, 1H, OH), 3.60 (s, 3H, OCH_3). ^{13}C NMR (151 MHz, DMSO-d_6) δ 153.26 (5-C), 152.19 (2'-C), 143.41 (5'-C), 131.61 (8-C, 9-C), 126.06 (q, *J* = 286.2 Hz, 2"-C), 125.52 (2-C), 112.36 (3-C), 111.32 (6-C), 111.16 (7-C), 110.49 (3'-C), 109.29 (4'-C), 102.43 (4-C), 73.18 (q, *J* = 30.5 Hz, 1"-C), 55.35 (OCH_3). ^{19}F NMR (565 MHz, DMSO-d_6) δ -76.13 (CF_3). LC−MS (m/z) positive mode 312 $[M + H]^{1+}$. Purity by HPLC-UV (254 nm)-ESI-MS 99%. HRMS (ESI-QTOF) calculated for $C_{15}H_{12}F_3NO_3$ $[M + H]^+$: 312.0848; found: 312.0852.

2,2,2-Trifluoro-1-(5-methoxy-1*H*-indol-3-yl)-1-(thiophen-2-yl)ethan-1-ol (3h)

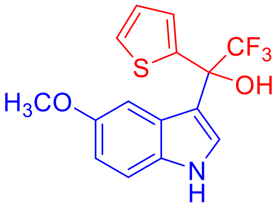

5-Methoxyindole (**1a**, 3.4 mmol), (2,2,2-trifluoro-1-(furan-2-yl)ethan-1-one (**2h**, 3.70 mmol), K_2CO_3 (15 mol %) and *n*-Bu_4PBr (15 mol %) were used for this reaction in water (5 mL). Light brown solid; yield: 98% (1.08 g); mp = 148-149 °C. ^1H NMR (600 MHz, DMSO-d_6) δ 11.30 (s, 1H, NH), 7.73 – 7.49 (m, 1H, 5'-H), 7.43 – 7.30 (m, 1H, 7-H), 7.30 – 7.17 (m, 2H, 2-H, 3'-H), 7.13 – 6.81 (m, 2H, 4'-H, 6-H), 6.81 – 6.51 (m, 2H, 4-H, OH), 3.57 (s, 3H, OCH_3). ^{13}C NMR (151 MHz, DMSO-d_6) δ 153.08 (5-C), 144.43 (2'-C), 131.68 (8-C, 9-C), 126.77 (5'-C, 4'-C), 126.53 (3'-C), 126.50 (2-C), 126.08 (q, *J* = 286.4 Hz, 2"-C), 112.34 (3-C, 6-C), 111.35 (7-C), 102.95 (4-C), 74.70 (q, *J* = 30.5 Hz, 1"-C), 55.28 (OCH_3). ^{19}F NMR (565 MHz, DMSO-d_6) δ -76.20 (CF_3). LC−MS (m/z) positive mode 328 $[M + H]^{1+}$. Purity by HPLC-UV (254 nm)-ESI-MS 97%. HRMS (ESI-QTOF) calculated for $C_{15}H_{12}F_3NO_2S$ $[M + H]^+$: 328.0619 found: 328.0633.

2,2-Difluoro-1-(5-methoxy-1*H*-indol-3-yl)-1-phenylethan-1-ol (3i)

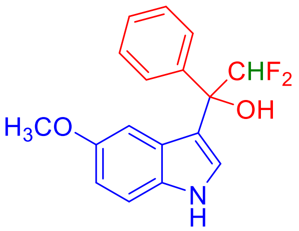

5-Methoxyindole (**1a**, 3.4 mmol), 2,2-difluoro-1-phenylethan-1-one (**2i**, 3.70 mmol), K_2CO_3 (15 mol %) and *n*-Bu_4PBr (15 mol %) were used for this reaction in water (5 mL). Brown solid; yield: 63% (0.650 g); mp = 185-186 °C. ^{1}H NMR (500 MHz, DMSO-d_6) δ 10.93 (s, 1H, NH), 7.51 – 7.45 (m, 2H, 2'-H, 6'-H), 7.35 – 7.19 (m, 5H, 3'-H, 4'-H, 5'-H, 4-H, 7-H), 6.67 (dd, *J* = 8.8, 2.5 Hz, 1H, 6-H), 6.58 (t, *J* = 2.3 Hz, 1H, 2-H), 6.17 (s, 1H, 2''-H), 3.51 (s, 3H, OCH_3). ^{13}C NMR (151 MHz, DMSO-d_6) δ 152.74 (5-C), 141.31 (1'-C), 131.71 (3'-C, 5'-C), 127.66 (8-C), 127.27 (9-C), 127.21 (2'-C), 126.18 (6'-C), 123.98 (4'-C), 118.65 (2-C), 117.00 (t, *J* = 247.4 Hz, 2''-C), 112.04 (3-C), 111.04 (6-C, 7-C), 103.14 (4-C), 74.88 (t, *J* = 21.3 Hz, 1''-C), 55.24 (OCH^3). ^{19}F NMR (565 MHz, DMSO-d_6) δ -126.11 (dd, *J* = 271.4 Hz, *J* = 56.3 Hz), -127.67 (dd, *J* = 271.3 Hz, *J* = 56.3 Hz). LC−MS (m/z) positive mode 304 $[M + H]^{1+}$. Purity by HPLC-UV (254 nm)-ESI-MS 96%. HRMS (ESI-QTOF) calculated for $C_{17}H_{15}F_2NO_2$ $[M + H]^+$: 304.1149 found: 304.1153.

2-Chloro-2,2-difluoro-1-(5-methoxy-1*H*-indol-3-yl)-1-phenylethan-1-ol (3m)

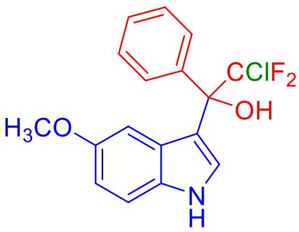

5-Methoxyindole (**1a**, 3.4 mmol), 2-chloro-2,2-difluoro-1-phenylethan-1-one (**2m**, 3.70 mmol), K_2CO_3 (15 mol %) and *n*-Bu_4PBr (15 mol %) were used for this reaction in water (5 mL). White solid; yield: 94% (1.07 g); mp = 158-159 °C. ^{1}H NMR (600 MHz, DMSO-d_6) δ 11.04 (s, 1H, NH), 7.63 – 7.48 (m, 2H, 2'-H, 6'-H), 7.40 (q, *J* = 1.8 Hz, 1H, 7-H), 7.36 – 7.27 (m, 3H, , 3'-H, 5'-H, 4-H), 7.24 (d, *J* = 8.7 Hz, 1H, 6-H), 7.02 (s, 1H, 2-H), 6.67 (dd, *J* = 8.8, 2.5 Hz, 1H, 4'-H), 6.45 (d, *J* = 2.4 Hz, 1H, OH), 3.47 (s, 3H, OCH_3). ^{13}C NMR (151 MHz, DMSO-d_6) δ 152.86 (5-C), 140.37 (1'-C), 131.53 (q, *J* = 303.9 Hz, 2''-C), 127.92 (8-C, 9-C), 127.90 (3'-C, 5'-C), 127.59 (2'-C, 6'-C, 4'-C), 124.30 (2-C), 113.41 (3-C), 112.17 (6-C), 111.17 (7-C), 103.16 (4-C), 79.40 (t, *J* = 24.5 Hz, 1''-C)), 55.22 (OCH_3). ^{19}F NMR (565

MHz, DMSO-d_6) δ -58.84 ($CClF_2$). LC−MS (m/z) positive mode 338 $[M + H]^{1+}$. Purity by HPLC-UV (254 nm)-ESI-MS 98%. HRMS (ESI-QTOF) calculated for $C_{17}H_{14}ClF_2NO_2$ $[M + H]^+$: 338.0759 found: 338.0765.

2,2,3,3,3-Pentafluoro-1-(5-methoxy-1*H*-indol-3-yl)-1-phenylpropan-1-ol (3n)

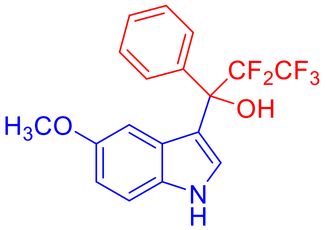

5-Methoxyindole (**1a**, 3.4 mmol), 2,2,3,3,3-pentafluoro-1-phenylpropan-1-one (**2n**, 3.70 mmol), K_2CO_3 (15 mol %) and *n*-Bu_4PBr (15 mol %) were used for this reaction in water (5 mL). Yellow solid; yield: 93% (1.17 g); mp = 168-169 °C. ^1H NMR (600 MHz, DMSO-d_6) δ 11.06 (s, 1H, NH), 7.66 – 7.50 (m, 2H, 2'-H, 6'-H), 7.43 (t, *J* = 2.1 Hz, 1H, 4'-H), 7.36 – 7.31 (m, 2H, 3'-H, 5'-H), 7.31 – 7.25 (m, 1H, 6-H), 7.24 (d, *J* = 8.8 Hz, 1H, 6-H), 7.06 (s, 1H, 2-H), 6.66 (dd, *J* = 8.8, 2.5 Hz, 1H, 7-H), 6.49 (d, *J* = 2.4 Hz, 1H, OH), 3.47 (s, 3H, OCH_3). ^{13}C NMR (151 MHz, DMSO-d_6) δ 152.81 (5-C), 139.74 (1'-C), 131.55 (4'-C), 127.90 (m, 3''-C), 127.73(8-C, 9-C), 127.32 (2'-C, 6'-C), 126.08 (3'-C, 5'-C), 123.99 (2-C), 118. 02 (m, 2''-C), 113.53 (3-C), 112.16 (6-C, 7-C), 111.15 (4-C), 103.07 (1''-C), 55.18 (OCH_3). ^{19}F NMR (565 MHz, DMSO-d_6) δ 116.78 (m, CF_2-CF_2), -76.11 (s, CF_3). LC−MS (m/z) positive mode 372 $[M + H]^{1+}$. Purity by HPLC-UV (254 nm)-ESI-MS 97%. HRMS (ESI-QTOF) calculated for $C_{18}H_{14}F_5NO_2$ $[M + H]^+$: 372.1023 found: 372.1027.

2,2,3,3,4,4,4-Heptafluoro-1-(5-methoxy-1*H*-indol-3-yl)-1-phenylbutan-1-ol (3o)

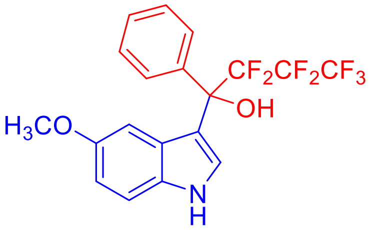

5-Methoxyindole (**1a**, 3.4 mmol), 2,2,3,3,4,4,4-heptafluoro-1-phenylbutan-1-one (**2o**, 3.70 mmol), K_2CO_3 (15 mol %) and *n*-Bu_4PBr (15 mol %) were used for this reaction in water (5 mL). Yellow solid; yield: 90% (1.29 g); mp = 161-163 °C. ^1H NMR (600 MHz, DMSO-d_6) δ 11.04 (s, 1H, NH), 7.62 – 7.50 (m, 2H, 2'-H, 6'-H), 7.43 (q, *J* = 2.2 Hz,

1H, 4'-H), 7.37 – 7.31 (m, 2H, 3'-H, 5'-H), 7.31 – 7.26 (m, 1H, 4-H), 7.24 (d, *J* = 8.7 Hz, 1H, 6-H), 7.06 (s, 1H, 2-H), 6.66 (dd, J = 8.8, 2.5 Hz, 1H, 7-H), 6.49 (d, *J* = 2.4 Hz, 1H, OH), 3.47 (s, 3H, OCH_3). ^{13}C NMR (151 MHz, DMSO-d_6) δ 152.81 (5-C), 139.74 (1'-C), 131.54 4'-C), 127.89 (8-C, 9-C), 127.73 (2'-C, 6'-C), 127.31 (3'-C), 126.08 (5'-C), 124.00 (2-C) , 120.21 (m, 2''-C), 118.29 (m, 3''-C), 115.23 (m, 4''-C), 113.54 (3-C), 112.15 (6-C), 111.15 (7-C), 103.06 (4-C), 76.30, 76.15, 76.00 (m, 1''-C), 55.17 (OCH_3). ^{19}F NMR (565 MHz, DMSO-d_6) δ 116.71 (m, CF_2), -76.11 (s, CF_3). LC–MS (m/z) positive mode 422 $[M + H]^{1+}$. Purity by HPLC-UV (254 nm)-ESI-MS 97%. HRMS (ESI-QTOF) calculated for $C_{19}H_{14}F_7NO_2$ $[M + H]^+$: 422.0991 found: 422.0997.

2,2,2-Trifluoro-1-(1*H*-indol-3-yl)-1-phenylethan-1-ol (**3p**)

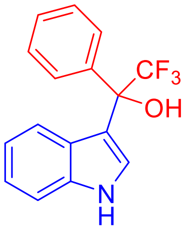

1*H*-Indole (**1b**, 3.4 mmol), 2,2,2-trifluoro-1-phenylethan-1-one (**2a**, 3.70 mmol), K_2CO_3 (15 mol %) and *n*-Bu_4PBr (15 mol %) were used for this reaction in water (5 mL). Orange solid; yield: 96% (1.20 g); mp = 122-124 °C. ^{1}H NMR (600 MHz, DMSO-d_6) δ 11.21 (s, 1H, NH), 7.49 (d, *J* = 7.3 Hz, 2H, 2'-H, 6'-H), 7.41 (t, *J* = 2.2 Hz, 1H, 4'-H), 7.37 (dd, *J* = 8.2, 1.1 Hz, 1H, 7-H), 7.34 – 7.27 (m, 3H, , 3'-H, 5'-H, 4-H), 7.05 – 6.97 (m, 2H, 5-H, 2-H), 6.95 (d, *J* = 0.7 Hz, 1H, OH), 6.78 (td, *J* = 7.5, 7.1, 1.2 Hz, 1H, 6-H). ^{13}C NMR (151 MHz, DMSO-d_6) δ 139.73 (1'-C), 136.54 (9-C), 128.07 (8-C), 127.80 (3'-C), 127.47 (5'-C), 127.2 (q, *J* = 282.7 Hz, 2''-C), 125.57 (2'-C), 123.59 (6'-C), 121.44 (2-C), 120.80 (4'-C), 118.85 (5-C, 6-C), 113.34 (3-C), 111.69 (4-C), 75.97, (q, *J* = 28.9 Hz, 1''-C)). ^{19}F NMR (565 MHz, DMSO-d_6) δ -75.12 (CF_3). LC–MS (m/z) positive mode 292 $[M + H]^{1+}$. Purity by HPLC-UV (254 nm)-ESI-MS 98%. HRMS (ESI-QTOF) calculated for $C_{16}H_{12}F_3NO$ $[M + H]^+$: 292.0942 found: 292.0950.

2,2,2-Trifluoro-1-(4-methoxy-1*H*-indol-3-yl)-1-phenylethan-1-ol (**3q**)

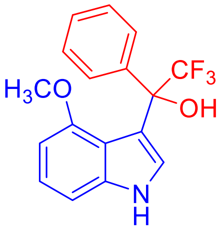

4-Methoxyindole (**1c**, 3.4 mmol), 2,2,2-trifluoro-1-phenylethan-1-one (**2a**, 3.70 mmol), K_2CO_3 (15 mol %) and *n*Bu_4PBr (15 mol %) were used for this reaction in water (5 mL). White solid; yield: 79% (0.86 g); mp = 160-161 °C. ^{1}H NMR (600 MHz, DMSO-d_6) δ 11.44 (s, 1H, NH), 7.45 – 7.35 (m, 2H, 2'-H, 6'-H), 7.34 – 7.22 (m, 4H, 3'-H, 4'-H, 5'-H, 5-H), 7.09 – 6.97 (m, 2H, 2-H, 6-H), 6.43 (dd, *J* = 7.5, 1.1 Hz, 1H, 7-H), 6.21 (s, 1H, OH), 3.46 (s, 3H, OCH_3). ^{13}C NMR (151 MHz, DMSO-d_6) δ 151.73 (4-C), 141.01 (1'-C), 138.31 (4'-C), 127.78 (8-C), 127.54 (9-C), 127.37 (2'-C, 6'-C), 125.03 (q, *J* = 284.5 Hz, (2"-C), 122.84 (3'-C, 5'-C), 115.38 (5-C), 113.12 (3-C), 105.72 (6-C), 100.78 (7-C), 76.47, 76.29, 76.10 (q, *J* = 28.3 Hz, 1"-C), 55.28 (OCH_3). ^{19}F NMR (565 MHz, DMSO-d_6) δ -72.17 (CF_3). LC−MS (m/z) positive mode 322 $[M + H]^{1+}$. Purity by HPLC-UV (254 nm)-ESI-MS 99%. HRMS (ESI-QTOF) calculated for $C_{17}H_{14}F_3NO_2$ $[M + H]^+$: 322.1055 found: 322.1054.

2,2,2-Trifluoro-1-(6-methoxy-1*H*-indol-3-yl)-1-phenylethan-1-ol (**3r**)

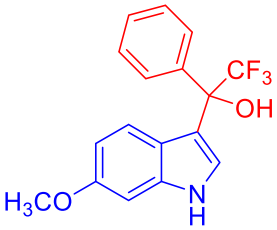

6-Methoxyindole (**1d**, 3.4 mmol), 2,2,2-trifluoro-1-phenylethan-1-one (**2a**, 3.70 mmol), K_2CO_3 (15 mol %) and *n*-Bu_4PBr (15 mol %) were used for this reaction in water (5 mL). Brown solid; yield: 90% (0.98 g); mp = 192-193°C. ^{1}H NMR (600 MHz, DMSO-d_6) δ 11.20 (s, 1H), 7.55 – 7.39 (m, 2H, 2'-H, 6'-H), 7.36 – 7.23 (m, 4H, 3'-H, 4'-H, 5'-H, 2-H), 6.92 (s, 1H, OH), 6.89 – 6.79 (m, 2H, 5-H, 7-H), 6.46 (dd, *J* = 8.9, 2.3 Hz, 1H, 4-H), 3.70 (s, 3H, OCH_3). ^{13}C NMR (151 MHz, DMSO-d_6) δ 155.73 (6-C), 139.76 (1'-C), 128.06 (8-C. 9-C), 127.60 (q, *J* = 286.0 Hz, (2"-C), 122.30 (2'-C, 6'-C), 121.33 (3'-C, 5'-C), 119.84 (2-C), 113.33 (3-C), 109.25 (5-C), 94.59 (4-C), 76.13 (q, *J* = 29.2 Hz, 1"-C), 55.29 (OCH_3). ^{19}F NMR (565 MHz, DMSO) δ -75.13 (CF_3). LC−MS (m/z) positive mode 322 $[M + H]^{1+}$. Purity by HPLC-UV (254 nm)-ESI-MS 97%. HRMS (ESI-QTOF) calculated for $C_{17}H_{14}F_3NO_2$ $[M + H]^+$: 322.1055 found: 322.1061.

2,2,2-Trifluoro-1-(5-fluoro-1*H*-indol-3-yl)-1-phenylethan-1-ol (3s)[1]

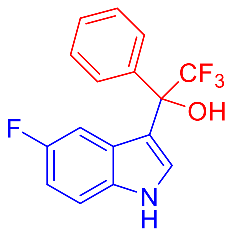

5-Fluoroindole (**1e**, 3.4 mmol), 2,2,2-trifluoro-1-phenylethan-1-one (**2a**, 3.70 mmol), K_2CO_3 (15 mol %) and *n*-Bu_4PBr (15 mol %) were used for this reaction in water (5 mL). White solid; yield: 94% (1.07 g); mp = 112-113°C. ^{1}H NMR (600 MHz, DMSO-d_6) δ 11.51 (s, 1H, NH), 7.59 – 7.43 (m, 3H, 4-H, 2'-H, 6'-H), 7.43 – 7.18 (m, 4H, 3'-H, 5'-H, 2H), 7.01 (s, 1H, OH), 6.88 (td, *J* = 9.1, 2.6 Hz, 1H, 7-H), 6.62 (dd, *J* = 10.4, 2.6 Hz, 1H, 6-H). ^{13}C NMR (151 MHz, DMSO-d_6) δ 157.27 (d, *J* = 231.4 Hz, 5-C), 139.37 (1'-C), 133.20 (4'-C), 128.25 (8-C, 9-C), 127.60 (3'-C, 5'-C), 125.66 (q, *J* = 286.1 Hz, 2"-C), 113.65 (2-C), 112.79 (3-C), 109.86 (7-C), 109.68 (6-C), 105.23 (4-C), 75.60 (q, *J* = 30.1 Hz, 1"-C). ^{19}F NMR (565 MHz, DMSO-d_6) δ -75.132 (s, CF_3), -124.85 (m, 5-F). LC−MS (m/z) positive mode 310 $[M + H]^{1+}$. Purity by HPLC-UV (254 nm)-ESI-MS 99%. HRMS (ESI-QTOF) calculated for $C_{16}H_{11}F_4NO$ $[M + H]^+$: 310.0855 found: 310.0857.

2,2,2-Trifluoro-1-(6-fluoro-1*H*-indol-3-yl)-1-phenylethan-1-ol (**3t**)

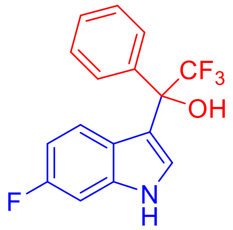

6-Fluoroindole (**1f**, 3.4 mmol), 2,2,2-trifluoro-1-phenylethan-1-one (**2a**, 3.70 mmol), K_2CO_3 (15 mol %) and *n*-Bu_4PBr (15 mol %) were used for this reaction in water (5 mL). White solid; yield: 96% (1.10 g); mp = 90-91 °C. ^{1}H NMR (600 MHz, DMSO-d_6) δ 11.27 (s, 1H, NH), 7.48 (dd, *J* = 7.6, 1.7 Hz, 2H, 2'-H, 6'-H), 7.41 (d, *J* = 2.5 Hz, 1H, 2-H), 7.37 – 7.28 (m, 3H, 3'-H, 4-H, 5'-H), 7.14 (dd, *J* = 10.0, 2.4 Hz, 1H, 7-H), 7.02 (s, 1H, OH), 6.97 (dd, *J* = 8.8, 5.5 Hz, 1H, 4-H), 6.67 (td, *J* = 9.4, 2.4 Hz, 1H, 5-H). ^{13}C NMR (151 MHz, DMSO-d_6) δ 159.55 (d, *J* = 235.2 Hz, 6-C), 139.48 (1'-C), 136.37 (4'-C), 128.17 (8-C, 9-C), 127.56 (q, *J* = 286.6 Hz, 2"-C), 124.97 (2'-C. 6'), 124.27 (3'-C, 5'-C), 122.35 (4'-C), 121.72 (2-C), 113.55 (3-C), 107.59 (5-C), 97.50 (7-C, 4-C), 75.73 (q, *J* = 30.1 Hz, (1"-C)). ^{19}F NMR (565 MHz, DMSO-d_6) δ -75.10 (s, CF_3), -

121.71 (m, 6-F). LC−MS (m/z) positive mode 310 [M + H]$^{1+}$. Purity by HPLC-UV (254 nm)-ESI-MS 99%. HRMS (ESI-QTOF) calculated for $C_{16}H_{11}F_4NO$ [M + H]$^{+}$: 310.0855 found: 310.0859.

2,2,2-Trifluoro-1-phenyl-1-(1*H*-pyrrolo[3,2-*b*]pyridin-3-yl)ethan-1-ol (3u)

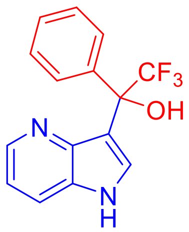

4-Azaindole (**1g**, 3.4 mmol), 2,2,2-trifluoro-1-phenylethan-1-one (**2a**, 3.70 mmol), K_2CO_3 (15 mol %) and *n*-Bu_4PBr (15 mol %) were used for this reaction in water (5 mL). Light brown solid; yield: 91% (1.12 g); mp = 165-166 °C. ^{1}H NMR (600 MHz, DMSO-d_6) δ 11.62 (s, 1H, NH), 8.26 (dd, *J* = 4.6, 1.4 Hz, 1H, 5-H), 7.95 (s, 1H, 2-H), 7.84 (dd, *J* = 8.3, 1.4 Hz, 1H, 7-H), 7.74 (s, 1H, 2-H), 7.69 – 7.61 (m, 2H, 2'-H, 6'-H), 7.35 (dd, *J* = 8.3, 6.6 Hz, 2H, 3'-H, 5'-H), 7.33 – 7.27 (m, 1H, 4'-H), 7.15 (dd, *J* = 8.2, 4.6 Hz, 1H, 6-H). ^{13}C NMR (151 MHz, DMSO-d_6) δ 144.24 (5-C), 142.24 (1'-C), 140.15 (7-C), 128.94 (8-C), 128.47 (9-C), 128.32 (3'-C, 5'-C), 127.23 (2'-C, 4'-C)', 125.56 (q, *J* = 286.6 Hz, 2''-C), 120.10 (2-C), 117.29 (6-C), 111.77 (3-C), 77.41 (q, *J* = 29.3 Hz, 1''-C). ^{19}F NMR (565 MHz, DMSO-d_6) δ -76.49 (CF_3). LC−MS (m/z) positive mode 293 [M + H]$^{1+}$. Purity by HPLC-UV (254 nm)-ESI-MS 98%. HRMS (ESI-QTOF) calculated for $C_{15}H_{11}F_3N_2O$ [M + H]$^{+}$: 293.0902 found: 293.0907.

2,2,2-Trifluoro-1-phenyl-1-(1*H*-pyrrolo[3,2-*c*]pyridin-3-yl)ethan-1-ol (3v)

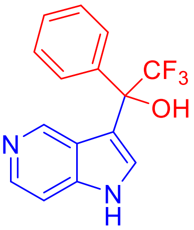

5-Azaindole (**1h**, 3.4 mmol), 2,2,2-trifluoro-1-phenylethan-1-one (**2a**, 3.70 mmol), K_2CO_3 (15 mol %) and *n*-Bu_4PBr (15 mol %) were used for this reaction in water (5 mL). Light brown solid; yield: 92% (1.17 g); mp = 227-228 °C. ^{1}H NMR (600 MHz, DMSO-d_6) δ 11.73 (s, 1H, NH), 8.73 (d, *J* = 1.1 Hz, 1H, 4-H), 7.89 (d, *J* = 5.5 Hz, 1H, 6-H), 7.49 (dd, *J* = 7.6, 2.0 Hz, 2H, 7-H, 4'-H), 7.40 – 7.18 (m, 3H, 2'-H, 3'-H, 6'-H), 7.11 (s, 1H, 2-H), 6.90 (dd, *J* = 5.5, 1.1 Hz, 1H, OH). ^{13}C NMR (151 MHz, DMSO-d_6) δ 139.33 (4-C), 137.73 (6-C), 134.95 (1'-C), 133.77 (4'-C), 133.76 (3'-C), 130.02 (5'-C), 128.33 (2'-C), 128.00 (6'-C), 127.51 (7-C), 127.36 (9-C), 126.87 (q, *J* = 287.7

Hz, 2"-C), 124.97 (2-C), 115.05 (3-C), 75.70 (q, J = 28.7 Hz, 1"-C). ^{19}F NMR (565 MHz, DMSO-d_6) δ -75.20 (CF_3). LC−MS (m/z) positive mode 293 $[M + H]^{1+}$. Purity by HPLC-UV (254 nm)-ESI-MS 97%.

22,2,2-Trifluoro-1-phenyl-1-(1*H*-pyrrolo[2,3-*c*]pyridin-3-yl)ethan-1-ol (3w)

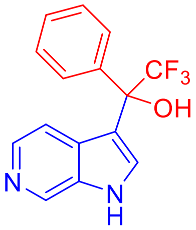

6-Azaindole (**1i**, 3.4 mmol), 2,2,2-trifluoro-1-phenylethan-1-one (**2a**, 3.70 mmol), K_2CO_3 (15 mol %) and *n*-Bu_4PBr (15 mol %) were used for this reaction in water (5 mL). White solid; yield: 97% (1.20 g); mp = 239-240 °C. ^{1}H NMR (600 MHz, DMSO-d_6) δ 11.73 (s, 1H, NH), 8.73 (d, J = 1.1 Hz, 1H, 7-H), 7.89 (d, J = 5.5 Hz, 1H, 5-H), 7.65 (dd, J = 2.0, 1.3 Hz, 1H, 4-H), 7.56 – 7.42 (m, 2H, 2'-H, 6'-H), 7.41 – 7.29 (m, 3H, 3'-H, 5'-H, 2H), 7.11 (s, 1H, OH), 6.90 (dd, J = 5.6, 1.1 Hz, 1H, 4'-H). ^{13}C NMR (151 MHz, DMSO-d_6) δ 139.33 (7-C), 137.73 (5-C), 134.95 (4-C), 133.77 (1'-C), 130.02 (4'-C), 128.33 (8'-C, 9-C), 128.00 (3'-C), 127.51 (5'-C), 125.40 (q, J = 287.4 Hz, 2"-C), 115.05 (2-C), 113.43 (3-C), 75.45 (q, J = 28.6 Hz, 1"-C). ^{19}F NMR (565 MHz, DMSO-d_6) δ -75.20 (CF_3). LC−MS (m/z) positive mode 293 $[M + H]^{1+}$. Purity by HPLC-UV (254 nm)-ESI-MS 99%. HRMS (ESI-QTOF) calculated for $C_{15}H_{11}F_3N_2O$ $[M + H]^+$: 293.0902 found: 293.0909.

2,2,2-Trifluoro-1-phenyl-1-(1*H*-pyrrolo[2,3-*b*]pyridin-3-yl)ethan-1-ol (3x)[1]

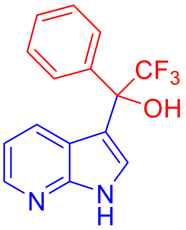

7-Azaindole (**1j**, 3.4 mmol), 2,2,2-trifluoro-1-phenylethan-1-one (**2a**, 3.70 mmol), K_2CO_3 (15 mol %) and *n*-Bu_4PBr (15 mol %) were used for this reaction in water (5 mL). White solid; yield: 90% (1.10 g); mp = 185-186 °C. ^{1}H NMR (600 MHz, DMSO-d_6) δ 11.80 (s, 1H, NH), 8.16 (dd, J = 4.6, 1.6 Hz, 1H, 6-H), 7.81 – 7.42 (m, 4H, 2'-H, 3'-H, 5'-H, 6'-H), 7.42 – 7.22 (m, 3H, 2-H, 4-H, 5-H), 7.11 (s, 1H, OH), 6.89 (dd, J = 8.0, 4.6 Hz, 1H, 4'-H). ^{13}C NMR (151 MHz, DMSO-d_6) δ 148.70 (9-C), 143.15 (6-C), 139.33 (1'-C), 128.79 (2'-C, 6'-C), 128.32 (3'-C, 5'-C), 127.99 (4'-C), 127.34 (q, J = 287.7 Hz, 2"-C), 124.09 (2-C), 117.93 (5-C), 117.93 (4-C), 115.56 (3-C), 75.80 (q, J =

28.8 Hz, 1''-C). ^{19}F NMR (565 MHz, DMSO-d_6) δ -75.08 (CF_3). LC−MS (m/z) positive mode 293 $[M + H]^{1+}$. Purity by HPLC-UV (254 nm)-ESI-MS 97%. HRMS (ESI-QTOF) calculated for $C_{15}H_{11}F_3N_2O$ $[M + H]^+$: 293.0902 found: 293.0910.

5-Methoxy-3-(2,2,2-trifluoro-1-(1*H*-indol-3-yl)-1-phenylethyl)-1*H*-indole (9)

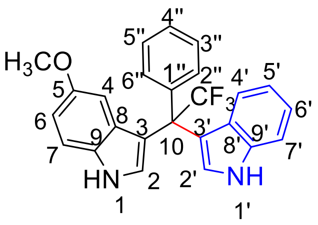

Light brown solid. yield: 81% (211 mg); ^{1}H NMR (600 MHz, DMSO-d_6) δ 11.16 (d, *J* = 2.8 Hz, 1H, NH), 11.13 (s, 1H, NH), 7.43 (dd, *J* = 6.7, 2.9 Hz, 2H, 4-H, 7-H), 7.40 (dt, *J* = 8.2, 1.0 Hz, 1H, 4'-H), 7.36 – 7.30 (m, 3H, 2''-H, 3''-H, 7'-H), 7.28 (d, *J* = 8.8 Hz, 1H, 4''-H), 7.08 – 6.93 (m, 2H, 3''-H, 5''-H), 6.92 (s, 1H, 6-H), 6.90 (q, *J* = 3.1, 2.0 Hz, 2H, 2-H, 2'-H), 6.77 (ddd, *J* = 8.2, 7.0, 1.1 Hz, 1H, 5'-H), 6.67 (dd, *J* = 8.8, 2.4 Hz, 1H, 7'-H), 6.18 (d, *J* = 2.4 Hz, 1H, 6'-H), 3.36 (s, 3H, OCH_3). ^{13}C NMR (151 MHz, DMSO) δ 152.76 (5-C), 139.26 (1''-C), 133.96 (9'-C), 131.98 (2''-C, 6''-C), 129.29 (3''-C, 5''-C), 128.11 (8-C), 128.10 (q, *J* = 286.6 Hz, CF_3), 126.95 (9-C), 126.10 (8'-C), 121.15 (4''-C), 118.88 (2-C, 2'-C), 113.24 (5'-C, 6'-C), 112.37 (7'-C), 110.95 (6-C, 7-C), 105.93 (3-C, 3'-C), 103.30 (4-C), 55.38 (q, *J* = 25.9 Hz, 10-C), 55.00 (OCH_3). ^{19}F NMR (565 MHz, DMSO) δ -62.36. LC−MS (m/z) positive mode 421 $[M + H]^{1+}$. Purity by HPLC-UV (254 nm)-ESI-MS 96%. HRMS (ESI-QTOF) calculated for $C_{25}H_{19}F_3N_2O$ $[M + H]^+$: 421.1528 found: 421.1532.

5-Methoxy-3-(2,2,2-trifluoro-1-phenyl-1-(2-phenyl-1*H*-indol-6-yl)ethyl)-1*H*-indole (10)

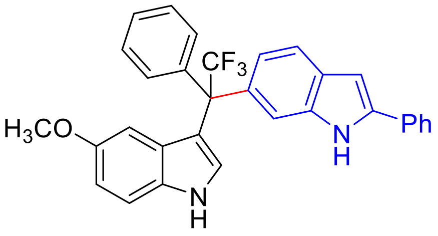

Colorless solid in 77% yield (237 mg), m.p. 234-235 ºC, ^{1}H NMR (600 MHz, DMSO-d_6) δ 11.81 (s, 1H), 11.10 (s, 1H, NH), 7.92 – 7.77 (m, 2H, Ar-H), 7.51 (d, *J* = 8.5 Hz, 1H, Ar-H), 7.44 – 7.40 (m, 2H, Ar-H), 7.38 (dd, *J* = 4.9, 1.8 Hz, 3H, 4-H, 7-H, 4'-H), 7.33 – 7.25 (m, 3H, 3''-H, 2-H, 5'-H, 6''-H), 7.23 – 7.18 (m, 1H, 4''-H), 7.03 – 6.94 (m, 1H, 5'-H), 6.90 (dd, *J* = 2.2, 0.9 Hz, 1H, 7'-H), 6.78 (d, *J* = 2.5 Hz, 1H, 3'-H), 6.71 (dd, *J* = 8.8, 2.4 Hz, 1H, 7-H), 6.07 (d, *J* = 2.3 Hz, 1H, 6-H), 3.34 (s, 3H, OCH_3). 13C NMR (151 MHz, DMSO) δ 152.92 (5-C), 140.18 (1''-C), 138.86 (2'-C), 136.90 (6'-C),

132.56 (Ar-C), 132.11 (2'-C, 6'-C), 129.45 (3'-C), 129.05 (5'-C), 128.24 (Ar-C), 127.76 (q, J = 286.1 Hz, CF_3), 127.50 (Ar-C), 126.61 (Ar-C), 125.09 (8-C, 9-C), 120.99 (8'-C, 9'-C), 119.58 (2-C), 113.65 (3-C), 112.92 (6-C, 7-C), 112.53 (5'-C, 7'-C) 111.05 (4-C), 60.18 (q, J = 25.9 Hz, 10-C), 55.04 (OCH_3). ^{19}F NMR (565 MHz, DMSO) δ -59.47 (CF_3). LC−MS (m/z) positive mode 497 $[M + H]^{1+}$. Purity by HPLC-UV (254 nm)-ESI-MS 96%. HRMS (ESI-QTOF) calculated for $C_{31}H_{23}F_3N_2O$ $[M + H]^+$: 497.1841 found: 497.1843.

5-Fluoro-3-(2,2,2-trifluoro-1-(5-methoxy-1*H*-indol-3-yl)-1-phenylethyl)-1*H*-indole (11)

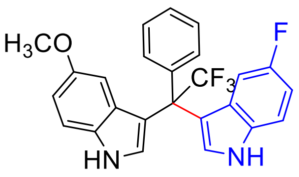

Brown solid, yield: 80% (217 mg), ^1H NMR (600 MHz, DMSO-d_6) δ 11.31 (s, 1H, NH), 11.08 (s, 1H, NH), 7.41 (p, J = 4.8, 4.2 Hz, 3H, 4-H, 7-H, 7'-H), 7.38 – 7.32 (m, 3H, 4"-H, 2"-H, 6"-H), 7.29 (d, J = 8.8 Hz, 1H, 4"-H), 7.04 (d, J = 2.7 Hz, 1H, 4'-H), 6.95 (d, J = 2.6 Hz, 1H, 6-H), 6.88 (td, J = 9.0, 2.5 Hz, 1H, 6'-H), 6.68 (dd, J = 8.8, 2.4 Hz, 1H, 7'-C), 6.47 – 6.36 (m, 1H, 2-H), 6.15 (d, J = 2.5 Hz, 1H, 2'-C), 3.36 (s, 3H OCH_3). ^{13}C NMR (151 MHz, DMSO) δ 157.28 (5'-C), 152.83 (5-C), 138.91 (1"-C), 133.63 91 (4"-C), 132.01 91 (2"-C, 6"-C), 129.24 (3'-C, 5"-C), 128.78 (8-C, 128.26 (q, J = 285.9 Hz, CF_3), 127.87 (8'-C, 9-C), 126.94 (9'-C), 126.47 (2-C, 2'-C), 112.47 (3-C, 3'-C), 111.05 (6-C, 7-C, 7'-C), 105.66 (4'-C, 6'-C), 103.16 (4-C), 55.1 (q, J = 25.9 Hz, Ar-F), 55.01 (OCH_3). ^{19}F NMR (565 MHz, DMSO) δ -62.58 (CF_3), -124.59, -124.60, -124.61, -124.61, -124.63 (5'-C). LC−MS (m/z) positive mode 439 $[M + H]^{1+}$. Purity by HPLC-UV (254 nm)-ESI-MS 97%. HRMS (ESI-QTOF) calculated for $C_{25}H_{18}F_4N_2O$ $[M + H]^+$: 439.1434 found: 439.1437.

X-ray crystal structure determination

The crystallographic studies have been performed on a Bruker X8-KappaApexII diffractometer (area detector Apex II) using graphite monochromated Mo Kα (λ = 0.71073 Å) irradiaton. The diffractometer was equipped with a low-temperature device (Bruker Kryoflex I (Bruker AXS); 100(2) K). Intensities were measured by fine-slicing φ and ω-scans and corrected for background, polarization and Lorentz effects. An empirical absorption correction was applied for all data sets. The structures were solved by the intrinsic phasing procedure implemented in ShelxT[2] and refined anisotropically by the least-squares procedure implemented in ShelxL.[3] Hydrogen atoms were included isotropically using the riding model on the bound carbon atoms. CCDC-1973322 contains the supplementary crystallographic data for this paper, which can be obtained free of charge from The Cambridge Crystallographic Data Centre via www.ccdc.cam.ac.uk/data_request/cif.

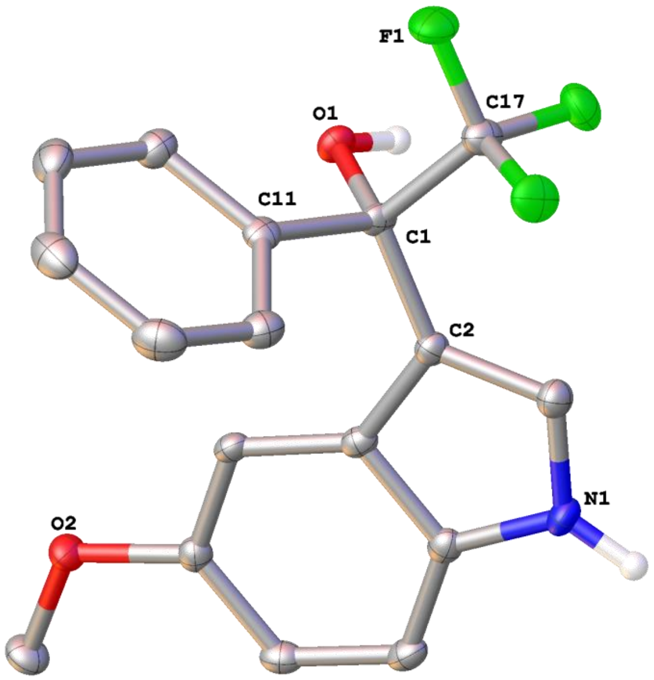

Figure S1. ORTEP-type plot of the molecular structure of **3a** (asymmetric unit) in the single crystal lattice at 100(2) K. The thermal ellipsoids are set at 50% probability.

Table S1 Crystal data and structure refinement for 6141f **3a**

Identification code	GPHARM89, YAZH-K921 // GXray6141f
Crystal Habitus	clear colourless plate
Device Type	Bruker X8-KappaApexII
Empirical formula	$C_{17}H_{14}NO_2F_3$
Moiety formula	C17 H14 F3 N O2
Formula weight	321.29
Temperature/K	100
Crystal system	monoclinic
Space group	$P2_1/c$
a/Å	9.6862(13)
b/Å	12.4199(16)
c/Å	12.3024(17)
α/°	90
β/°	97.169(4)
γ/°	90
Volume/Å^3	1468.4(3)
Z	4
ρ_{calc}g/cm^3	1.453
μ/mm^{-1}	0.121
F(000)	664.0
Crystal size/mm^3	0.35 × 0.25 × 0.16
Absorption correction	empirical
Tmin; Tmax	0.5856; 0.7462
Radiation	MoKα (λ = 0.71073)
2Θ range for data collection/°	5.36 to 55.998°
Completeness to theta	0.999
Index ranges	-12 ≤ h ≤ 12, -16 ≤ k ≤ 16, -16 ≤ l ≤ 16
Reflections collected	22299
Independent reflections	3545 [R_{int} = 0.0814, R_{sigma} = 0.0516]
Data/restraints/parameters	3545/0/210
Goodness-of-fit on F^2	1.016
Final R indexes [I>=2σ (I)]	R_1 = 0.0418, wR_2 = 0.0959

Final R indexes [all data]	R_1 = 0.0610, wR_2 = 0.1071
Largest diff. peak/hole / e Å^{-3}	0.35/-0.34

Table S2 Bond Lengths for 6141f **3a**

**Atom**	**Atom**	**Length/Å**		**Atom**	**Atom**	**Length/Å**
F1	C17	1.3305(18)		C3	C4	1.415(2)
F2	C17	1.3443(19)		C3	C6	1.397(2)
F3	C17	1.3461(18)		C4	C9	1.388(2)
O1	C1	1.4320(17)		C6	C7	1.383(2)
O2	C7	1.3923(18)		C7	C8	1.407(2)
O2	C10	1.4354(18)		C8	C9	1.381(2)
N1	C4	1.3802(19)		C11	C12	1.390(2)
N1	C5	1.369(2)		C11	C16	1.394(2)
C1	C2	1.507(2)		C12	C13	1.391(2)
C1	C11	1.527(2)		C13	C14	1.386(2)
C1	C17	1.540(2)		C14	C15	1.384(2)
C2	C3	1.442(2)		C15	C16	1.387(2)
C2	C5	1.373(2)				

Table S3 Bond Angles for 6141f **3a**

**Atom**	**Atom**	**Atom**	**Angle/˚**		**Atom**	**Atom**	**Atom**	**Angle/˚**
C7	O2	C10	117.63(12)		O2	C7	C8	123.09(13)
C5	N1	C4	108.96(12)		C6	C7	O2	115.10(13)
O1	C1	C2	110.74(12)		C6	C7	C8	121.79(14)
O1	C1	C11	107.33(12)		C9	C8	C7	120.27(14)
O1	C1	C17	105.65(12)		C8	C9	C4	118.18(14)
C2	C1	C11	112.85(12)		C12	C11	C1	119.82(13)
C2	C1	C17	111.55(12)		C12	C11	C16	118.93(15)
C11	C1	C17	108.38(12)		C16	C11	C1	121.20(13)
C3	C2	C1	123.76(13)		C13	C12	C11	120.58(15)
C5	C2	C1	129.42(14)		C14	C13	C12	120.15(16)
C5	C2	C3	106.74(13)		C15	C14	C13	119.46(16)
C4	C3	C2	106.41(13)		C14	C15	C16	120.60(16)
C6	C3	C2	134.44(14)		C15	C16	C11	120.25(15)
C6	C3	C4	119.15(13)		F1	C17	F2	106.94(12)
N1	C4	C3	107.87(13)		F1	C17	F3	107.14(12)
N1	C4	C9	130.07(14)		F1	C17	C1	111.63(13)
C9	C4	C3	122.06(14)		F2	C17	F3	107.00(12)
N1	C5	C2	110.02(13)		F2	C17	C1	111.46(13)
C7	C6	C3	118.51(13)		F3	C17	C1	112.36(12)

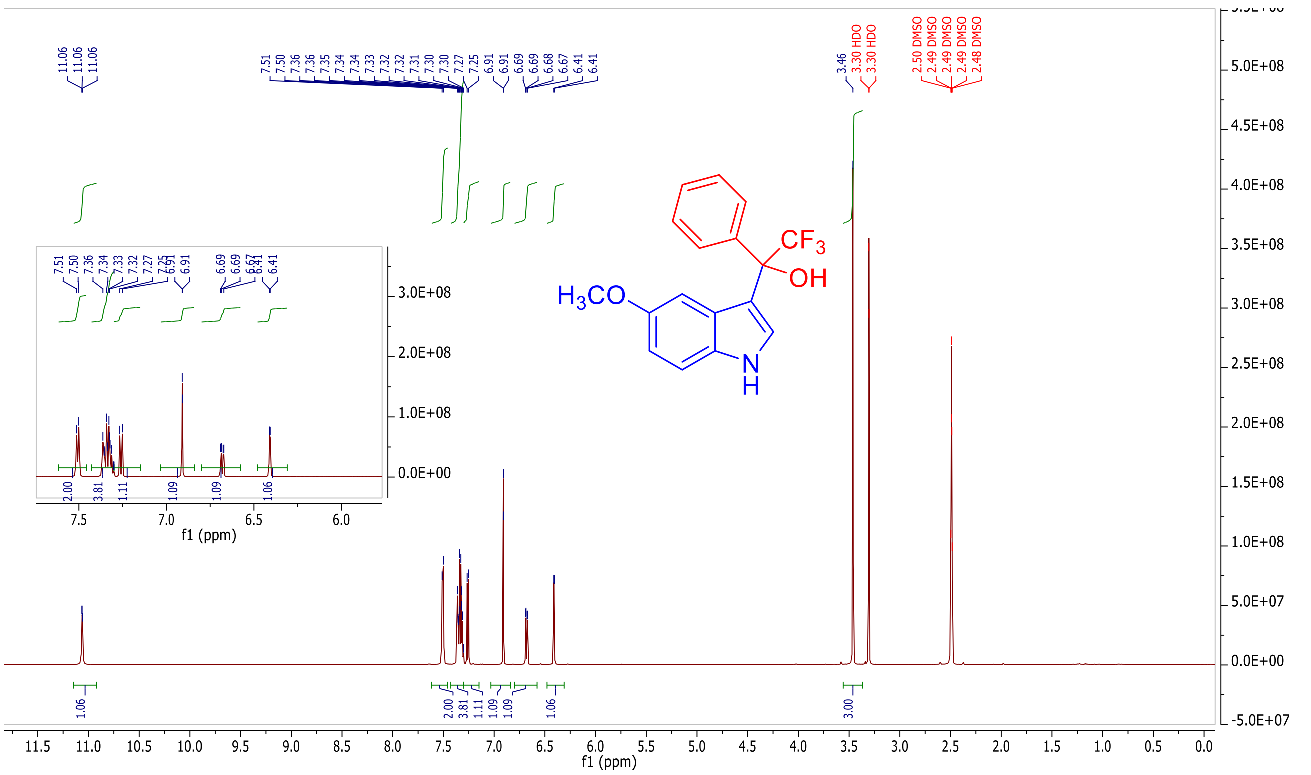

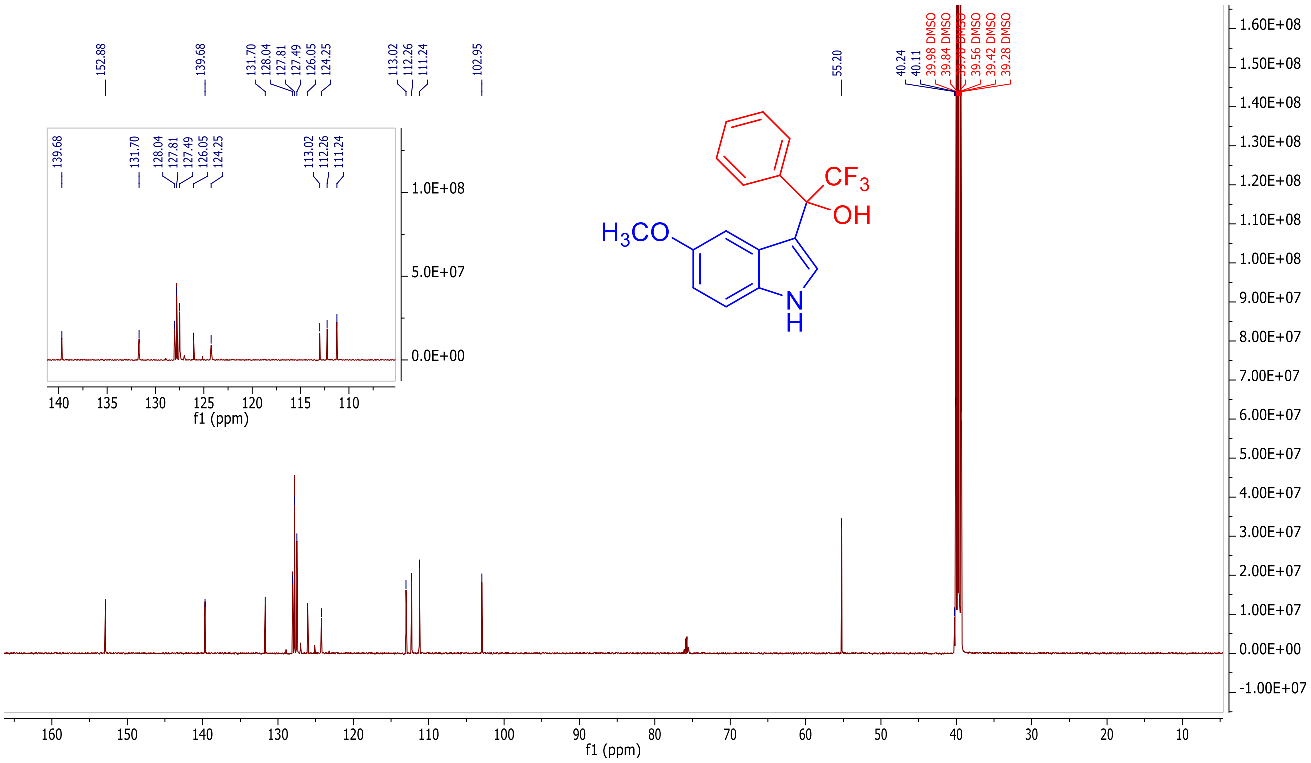

Figure S2. ^{1}H (600 MHz) and ^{13}C (151 MHz) Spectra of 2,2,2-trifluoro-1-(5-methoxy-1*H*-indol-3-yl)-1-phenylethan-1 (**3a**)

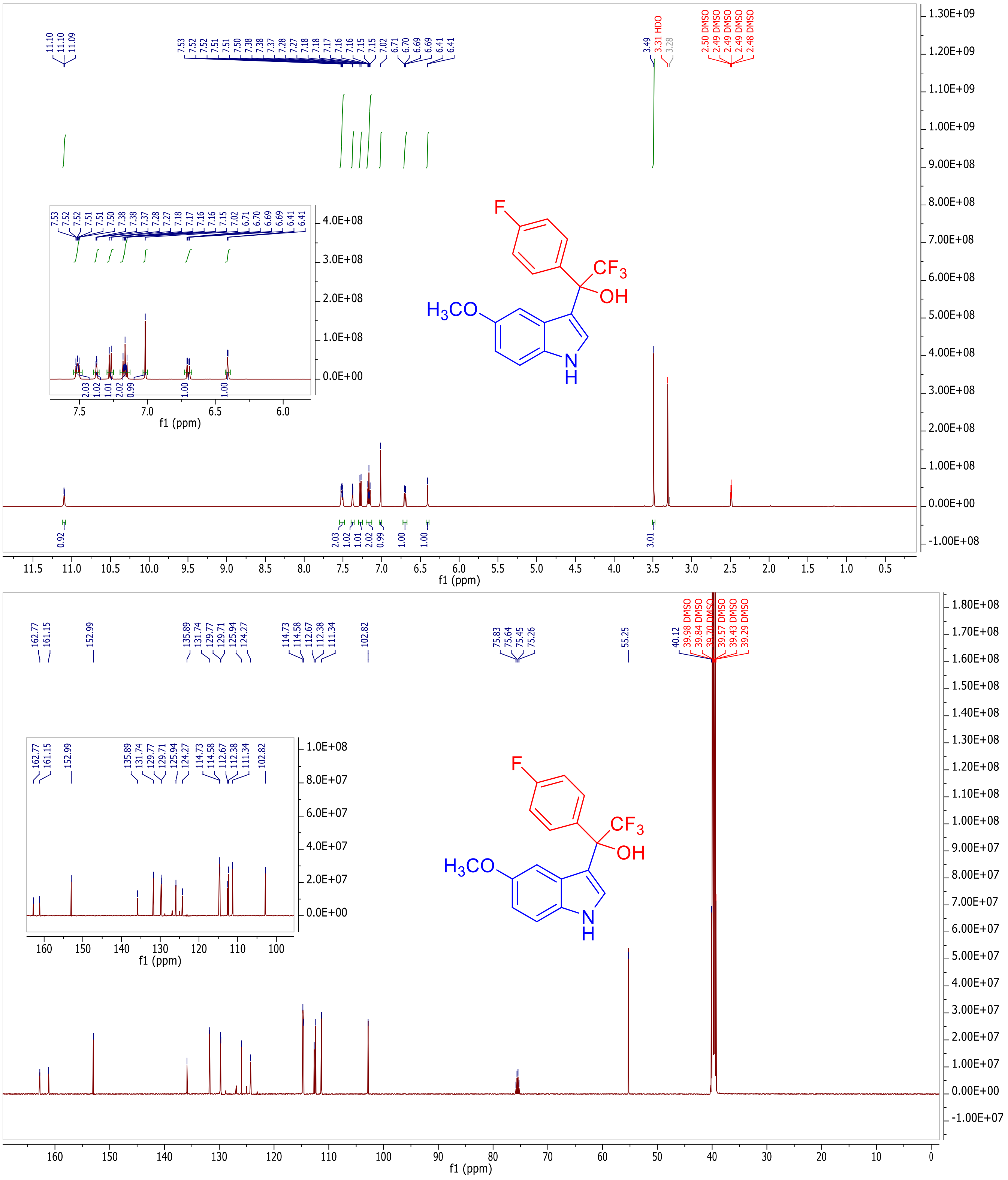

Figure S3. ^{1}H (600 MHz) and ^{13}C (151 MHz) Spectra of 2,2,2-trifluoro-1-(4-fluorophenyl)-1-(5-methoxy-1*H*-indol-3-yl)ethan-1-ol (**3b**)

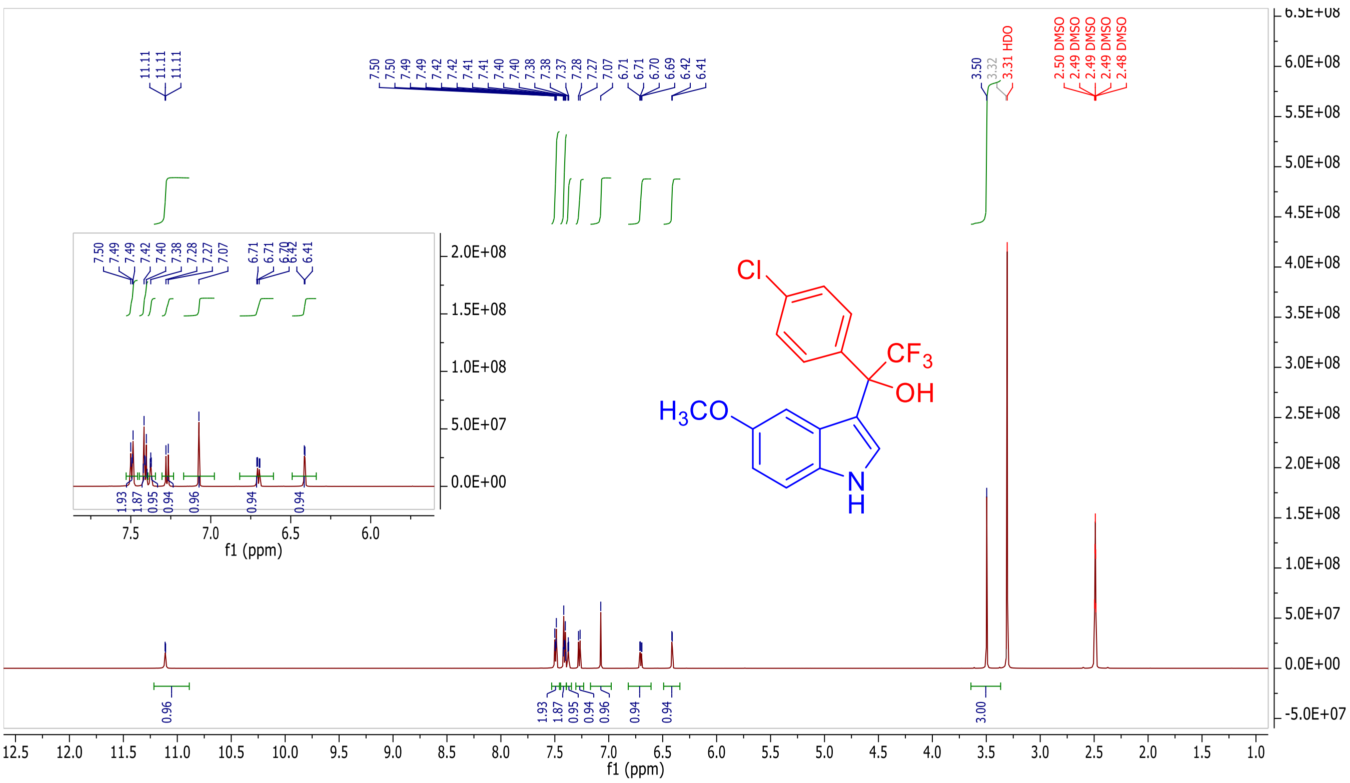

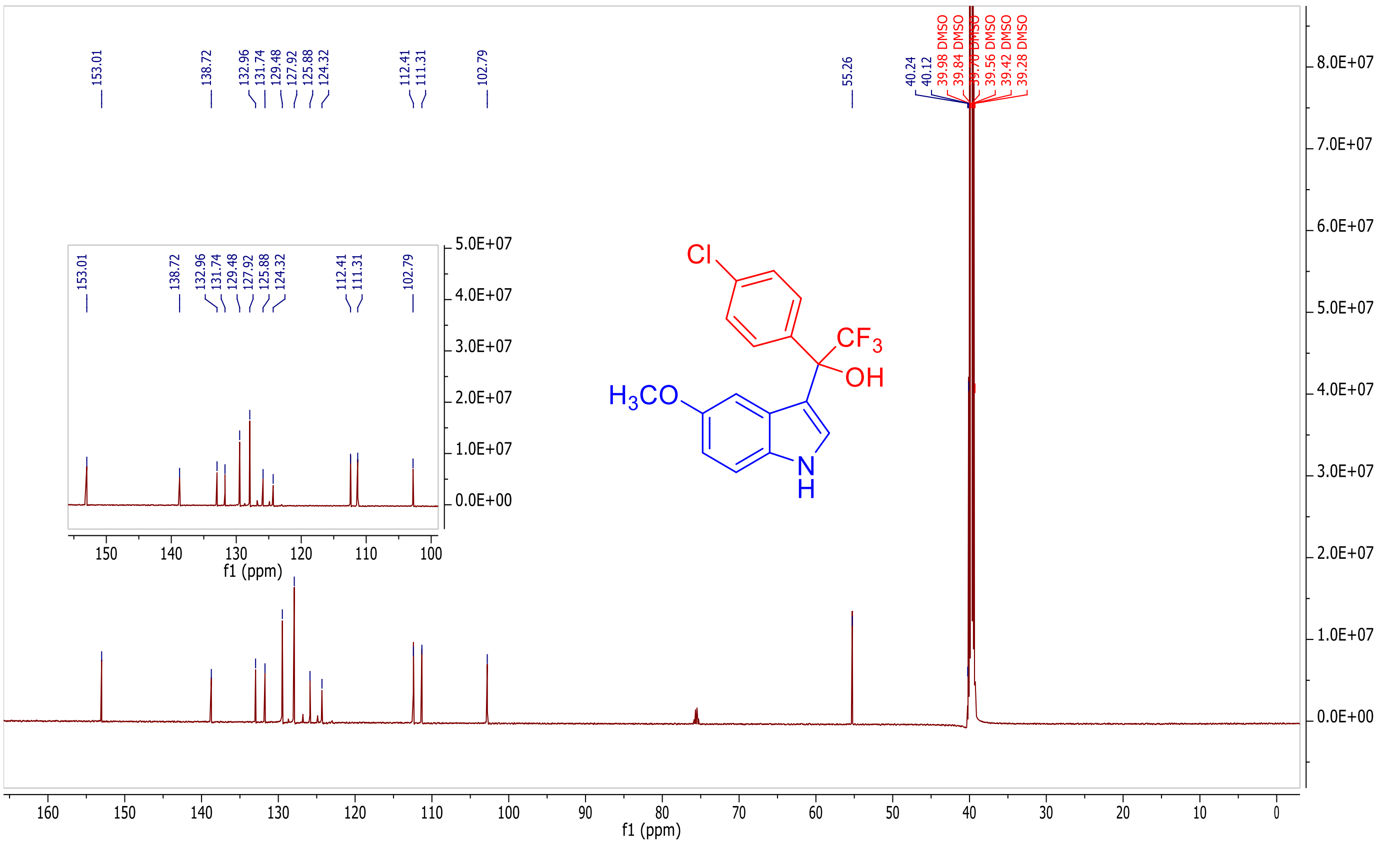

Figure S4. ^{1}H (600 MHz) and ^{13}C (151 MHz) Spectra of 1-(4-chlorophenyl)-2,2,2-trifluoro-1-(5-methoxy-1*H*-indol-3-yl)ethan-1-ol (**3c**)

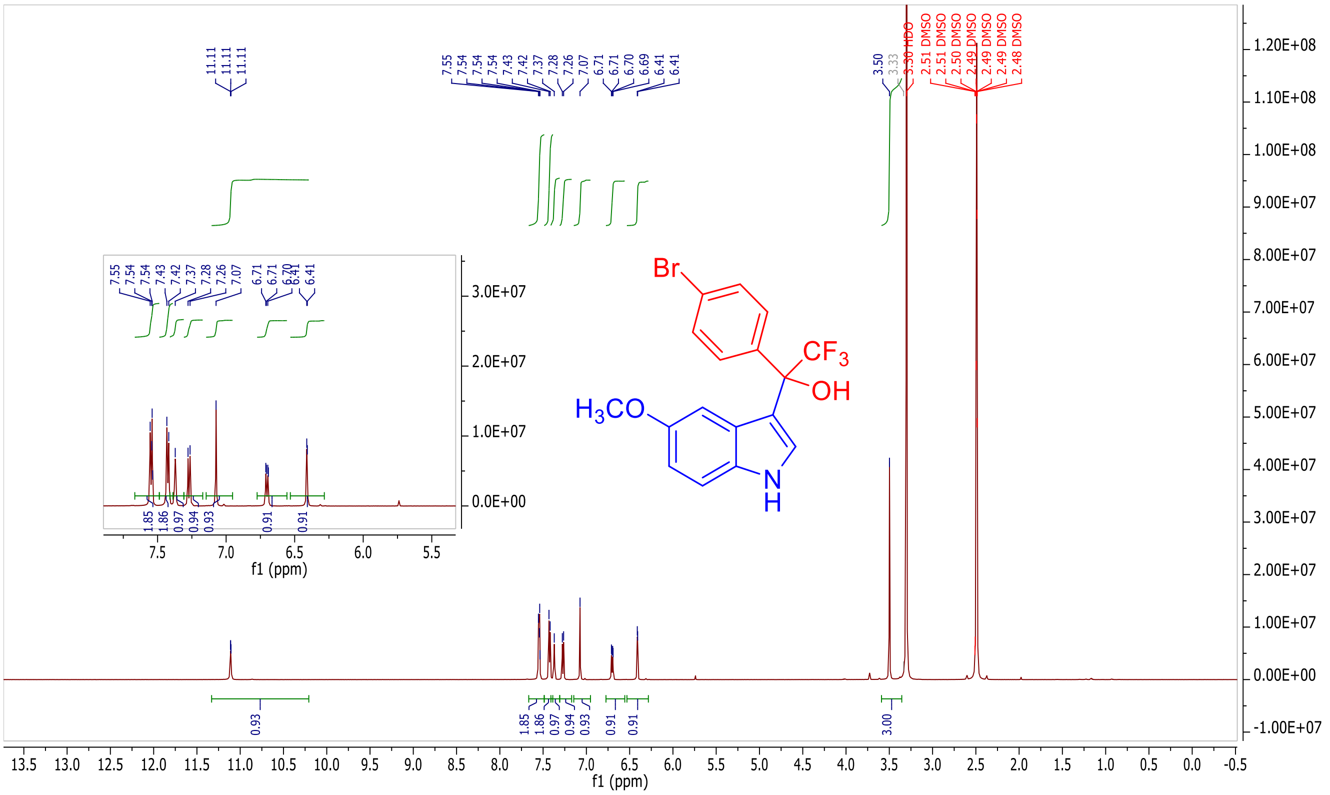

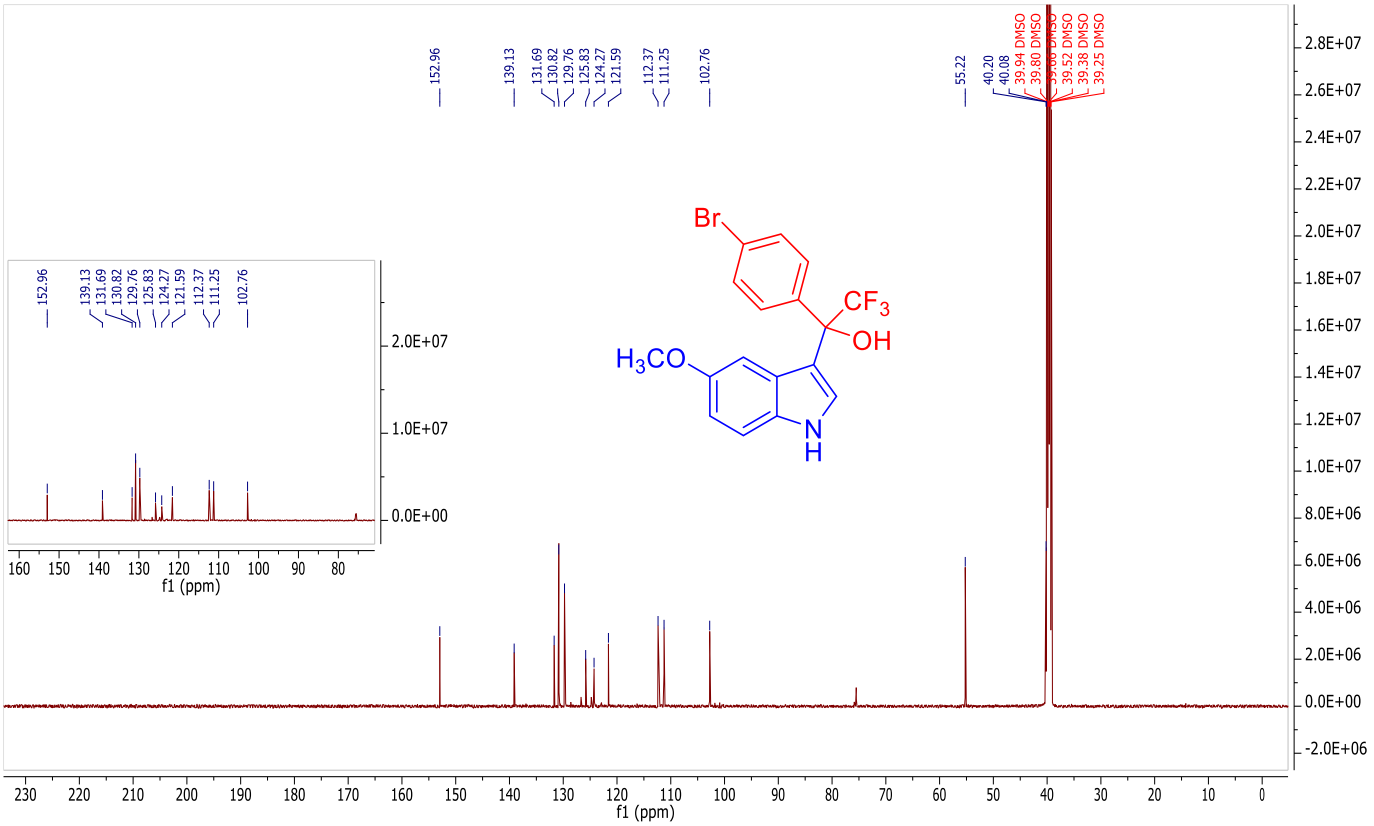

Figure S5. ^{1}H (600 MHz) and ^{13}C (151 MHz) Spectra of 1-(4-bromophenyl)-2,2,2-trifluoro-1-(5-methoxy-1*H*-indol-3-yl)ethan-1-ol (**3d**)

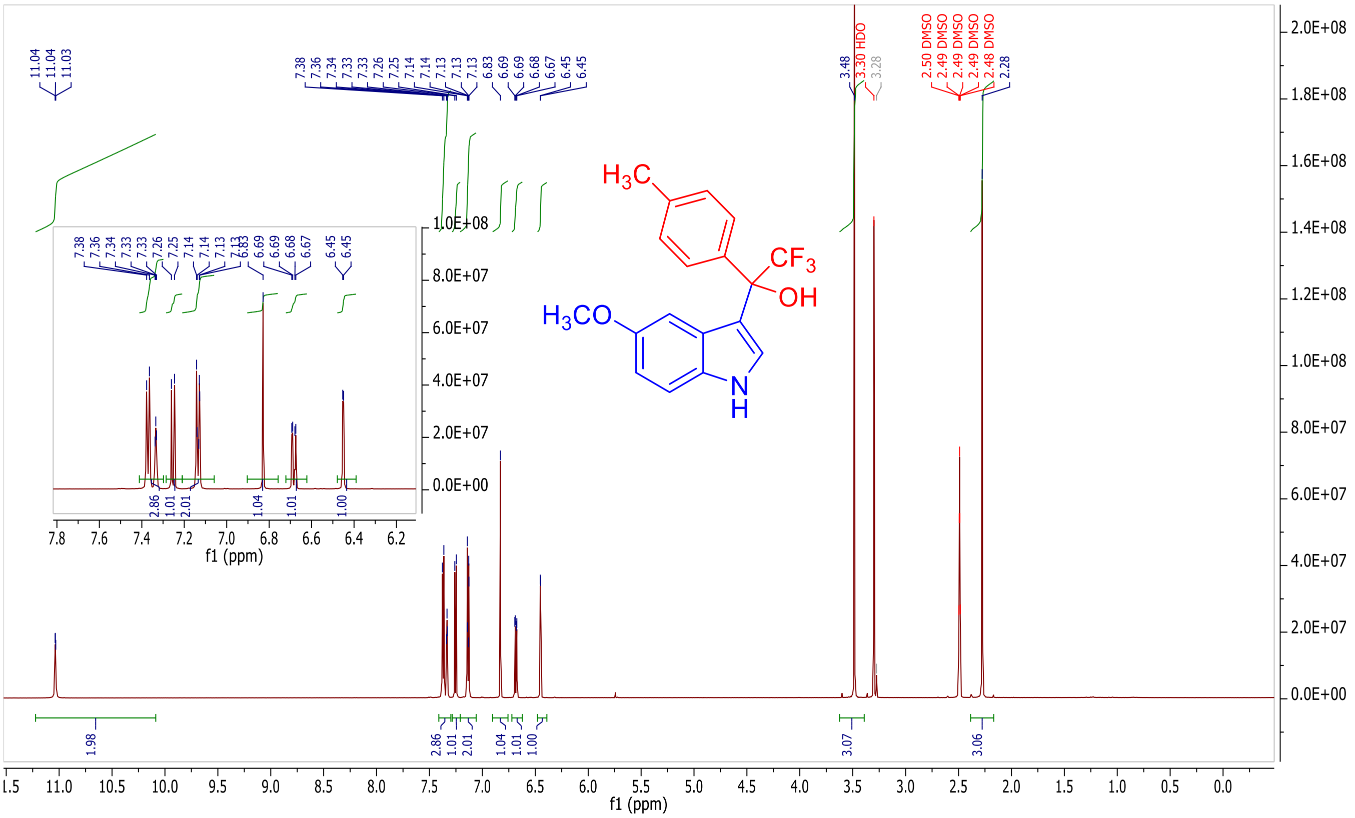

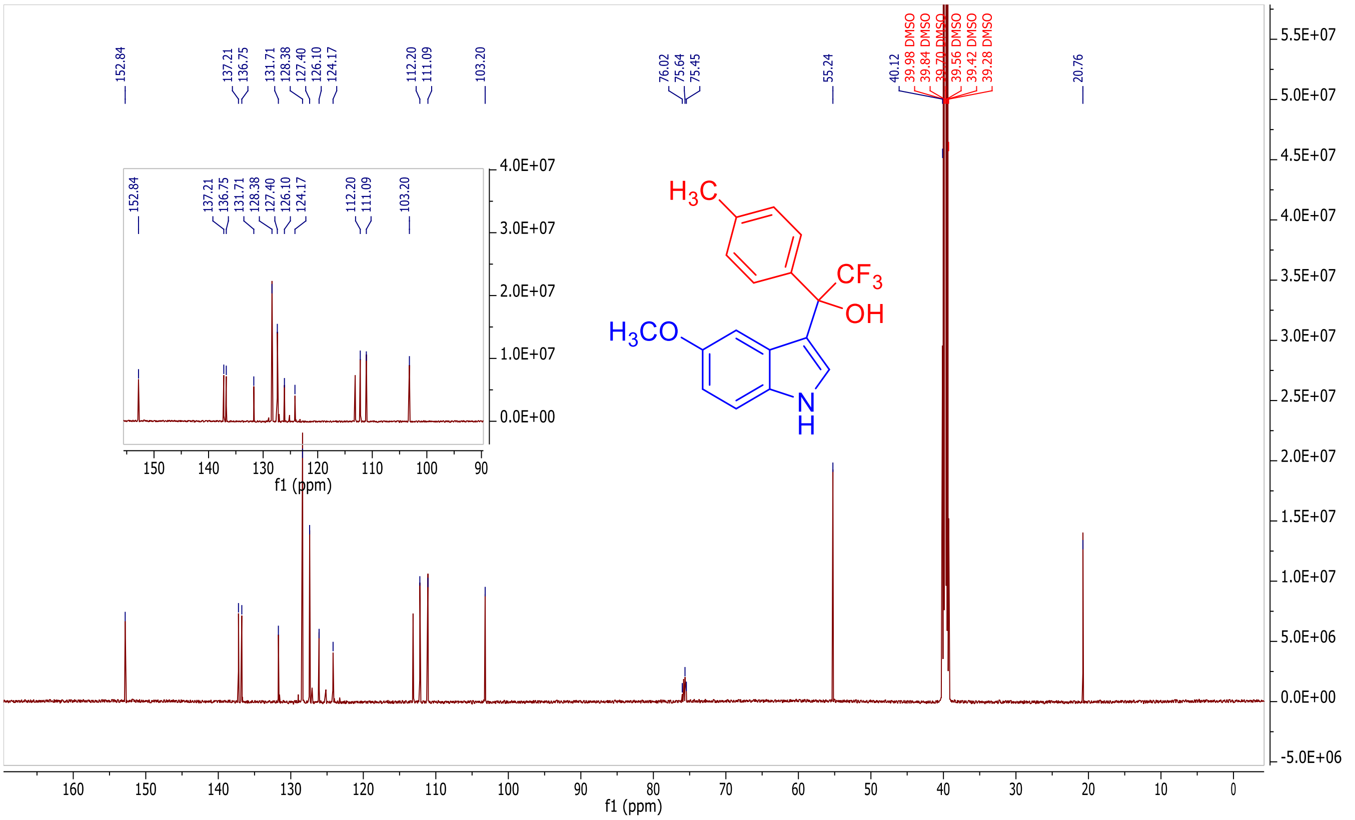

Figure S6. ^{1}H (600 MHz) and ^{13}C (151 MHz) Spectra of 2,2,2-trifluoro-1-(5-methoxy-1*H*-indol-3-yl)-1-(*p*-tolyl)ethan-1-ol (**3e**)

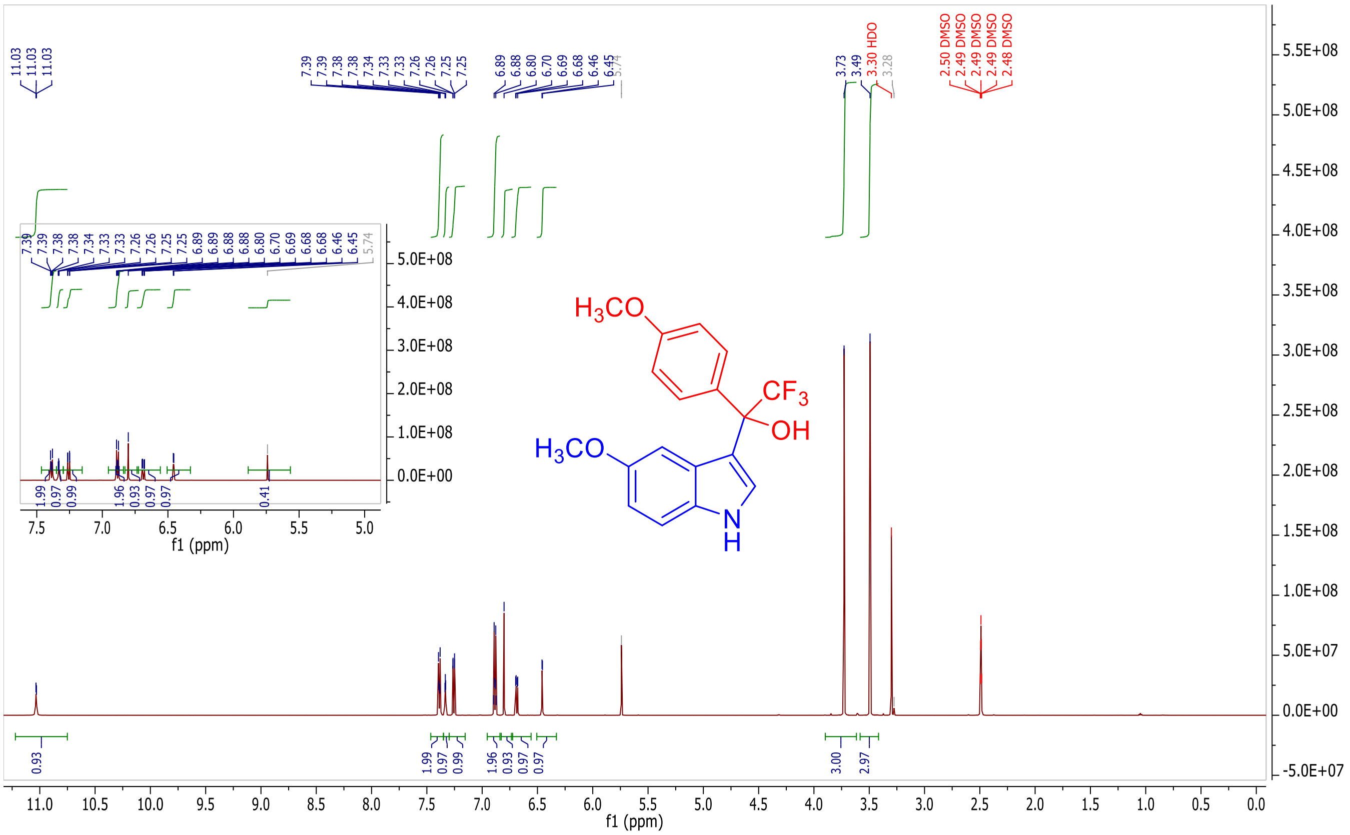

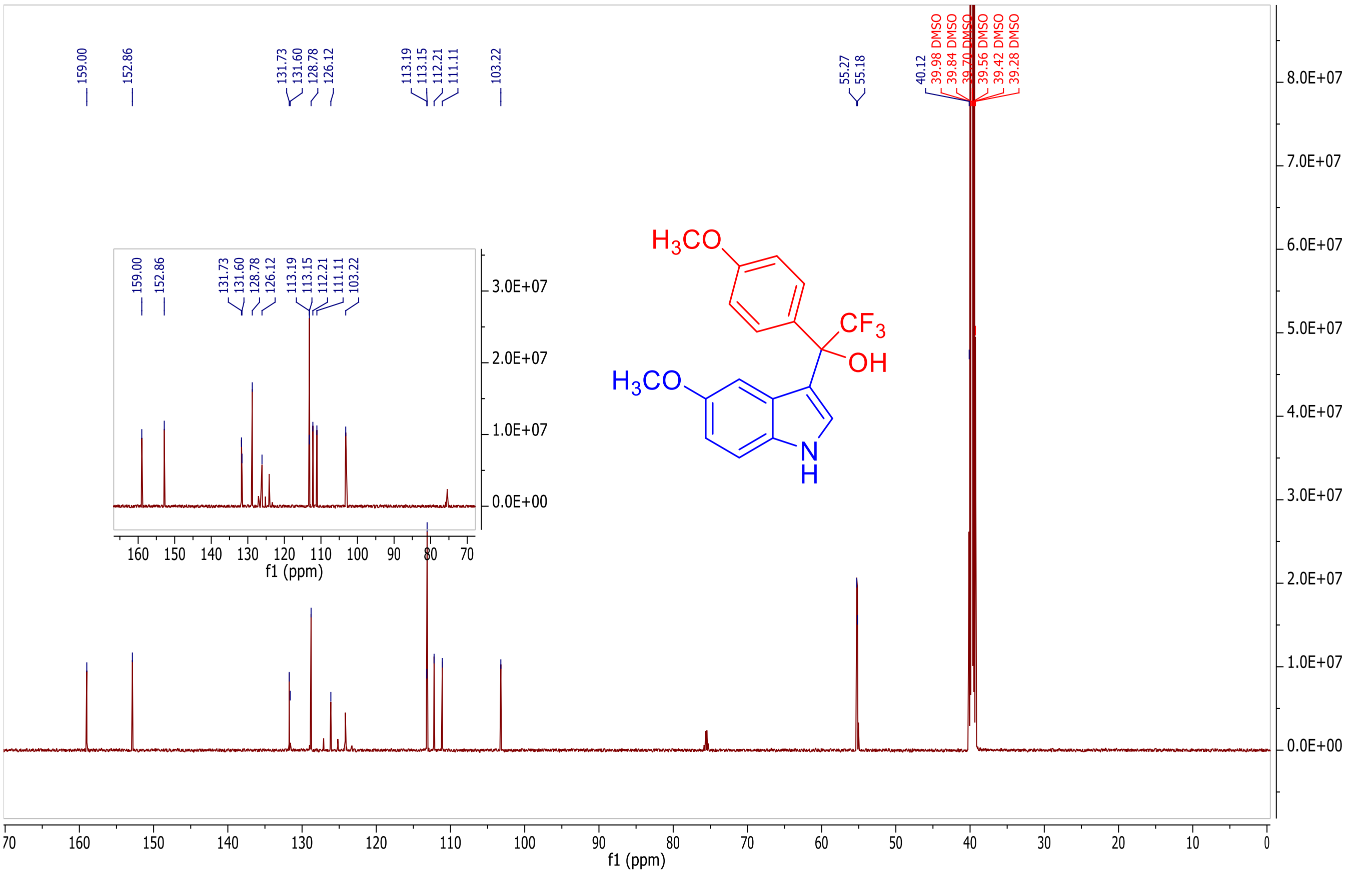

Figure S7. ^{1}H (600 MHz) and ^{13}C (151 MHz) Spectra of 2,2,2-trifluoro-1-(5-methoxy-1*H*-indol-3-yl)-1-(4-methoxyphenyl)ethan-1-ol (**3f**)

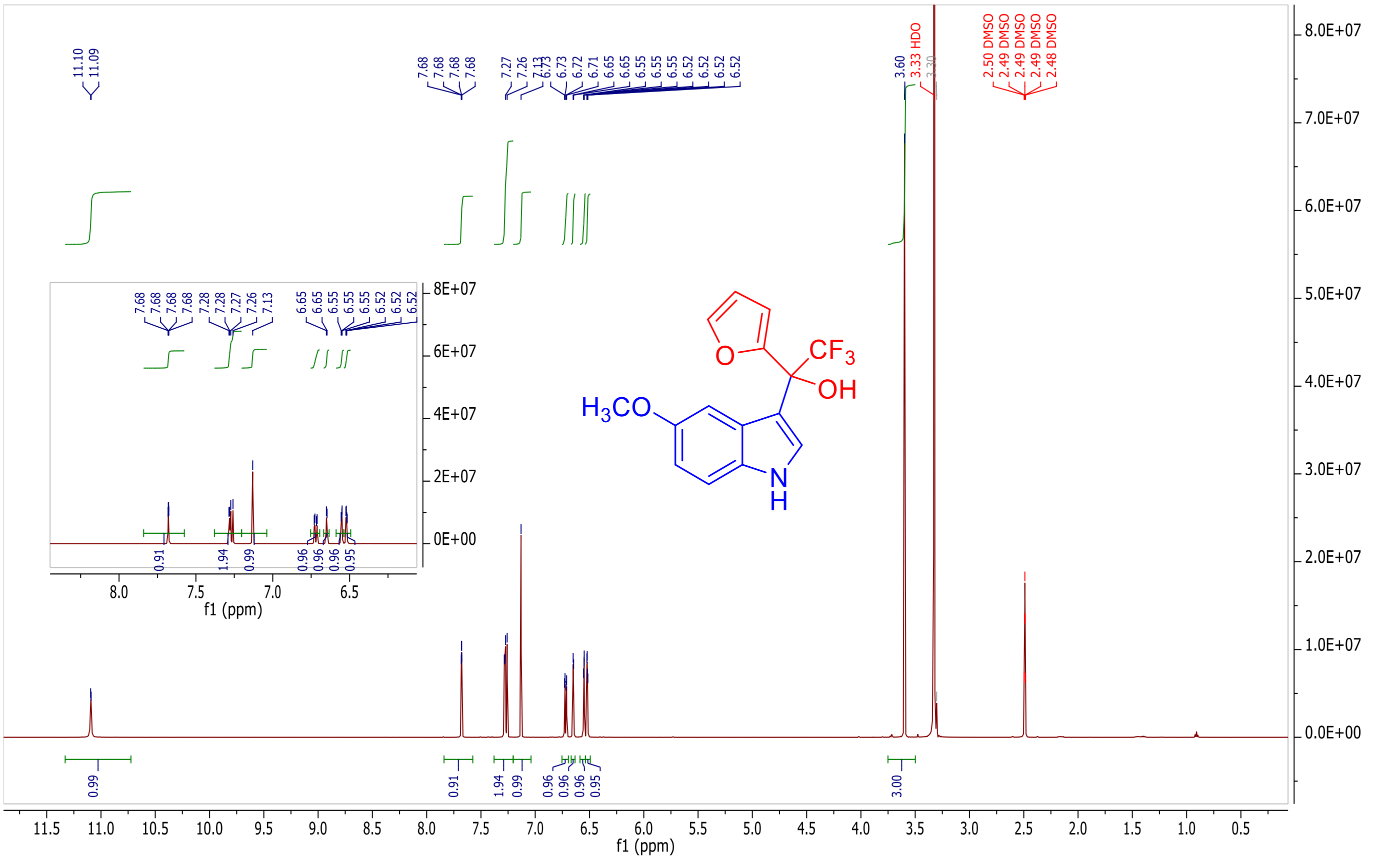

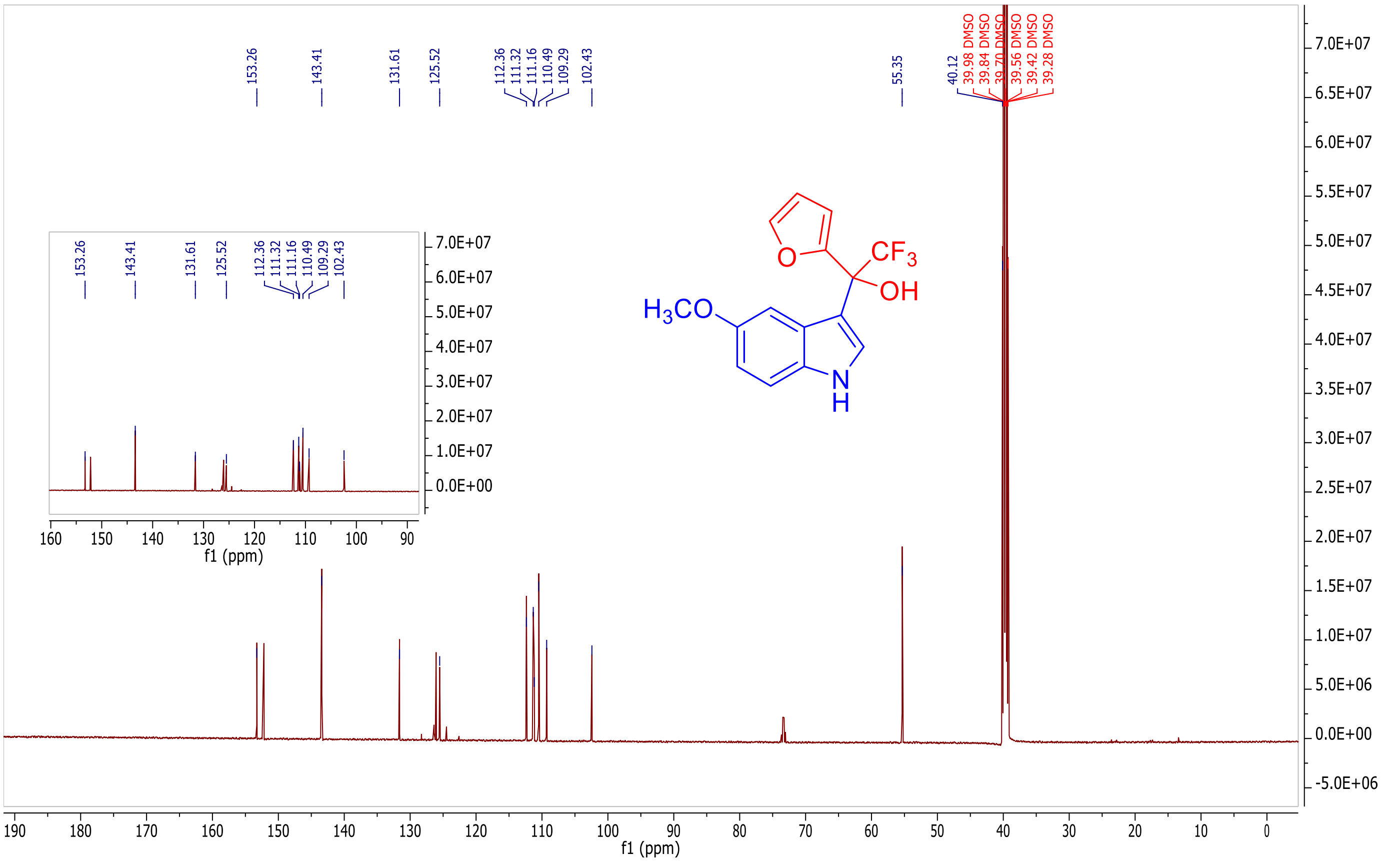

Figure S8. ^{1}H (600 MHz) and ^{13}C (151 MHz) Spectra of 2,2,2-trifluoro-1-(furan-2-yl)-1-(5-methoxy-1*H*-indol-3-yl)ethan-1-ol (**3g**)

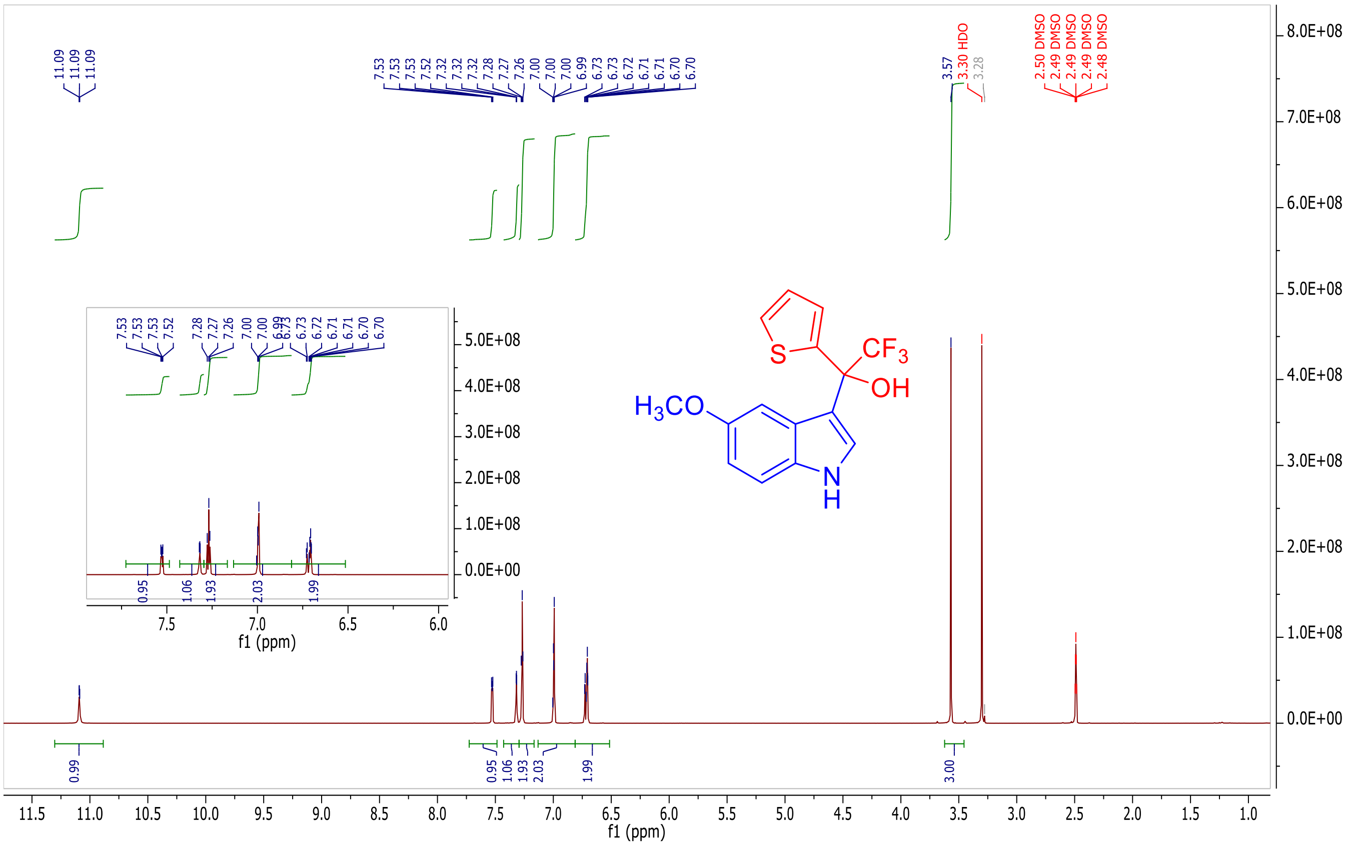

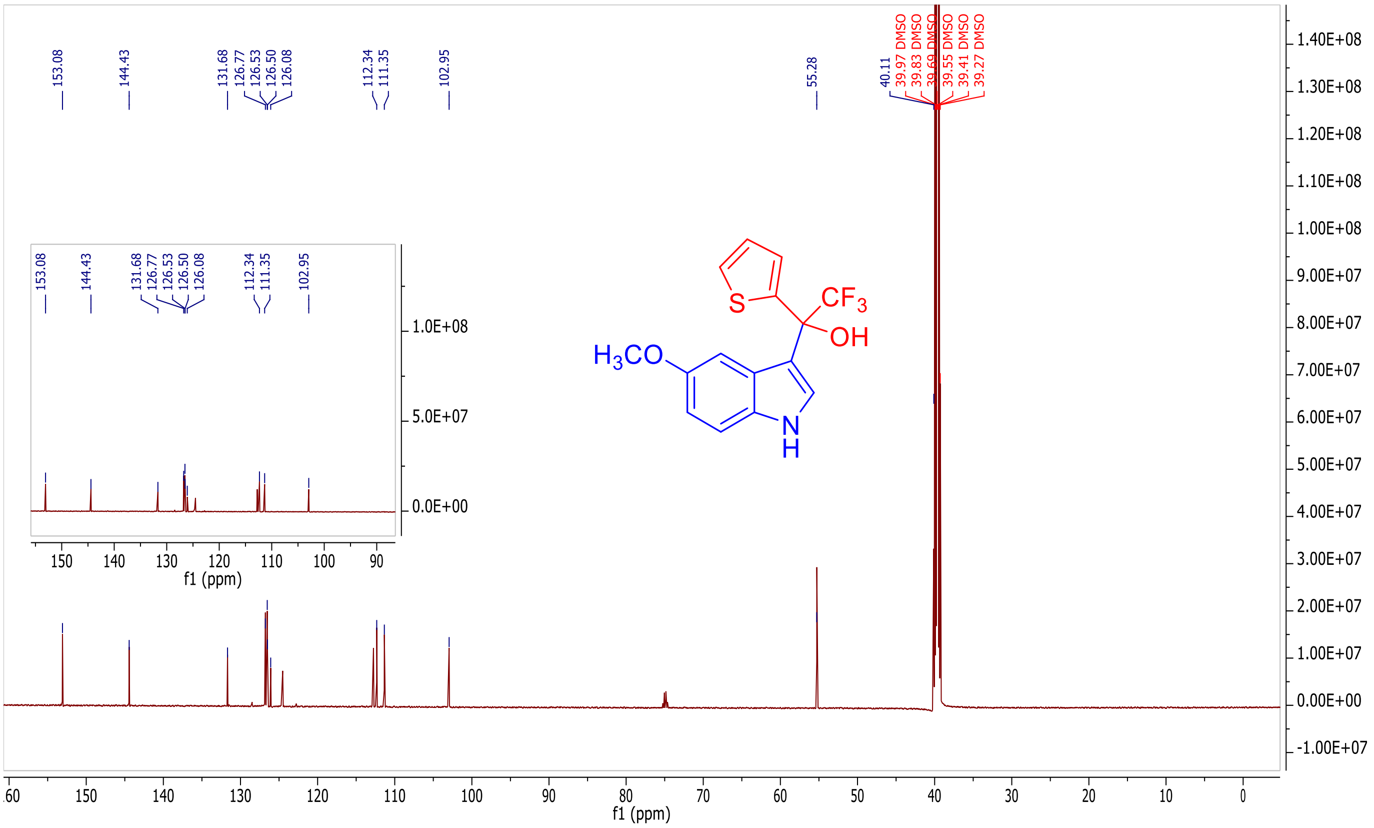

Figure S9. ^{1}H (600 MHz) and ^{13}C (151 MHz) Spectra of 2,2,2-trifluoro-1-(5-methoxy-1*H*-indol-3-yl)-1-(thiophen-2-yl)ethan-1-ol (**3h**)

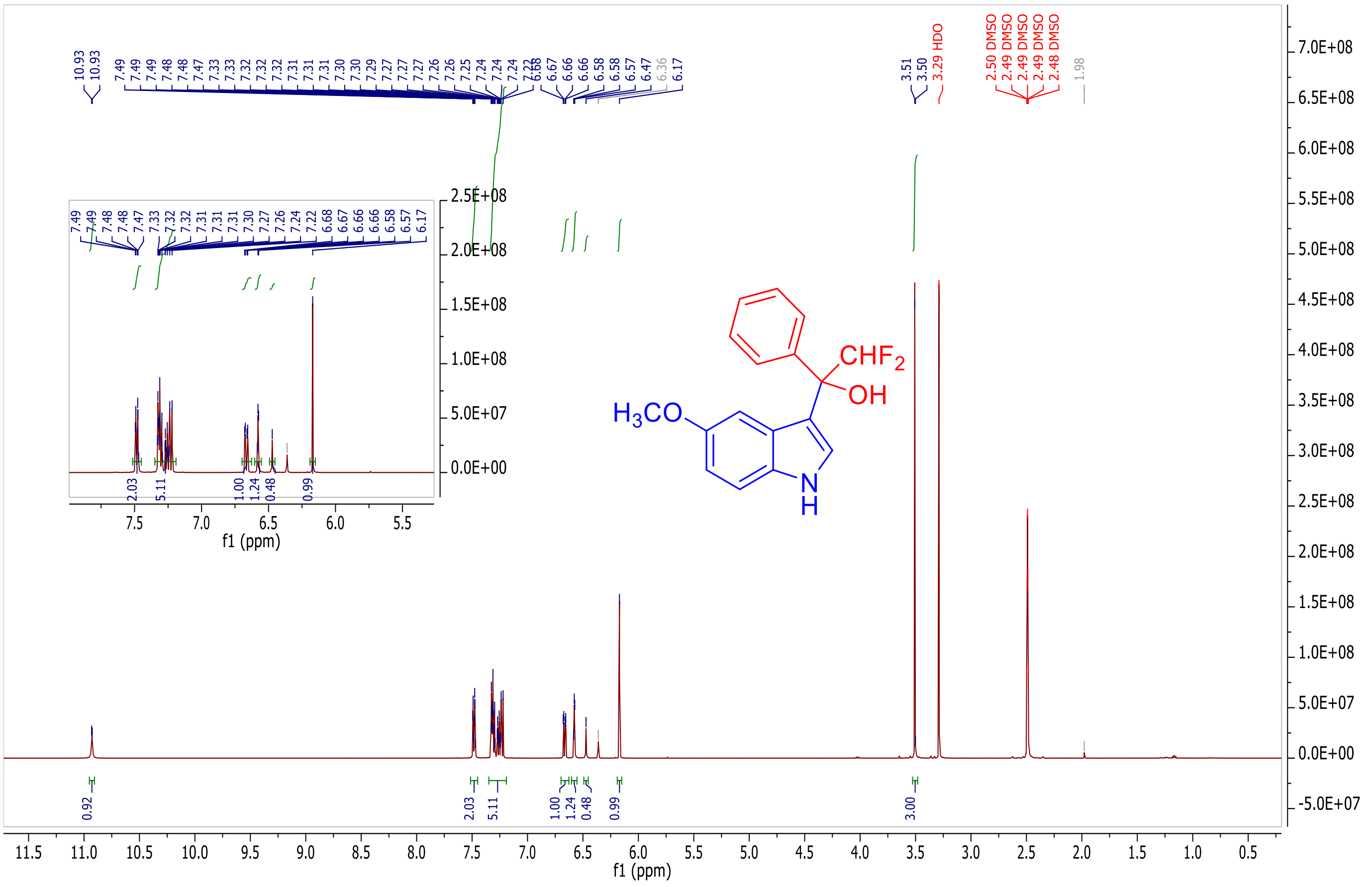

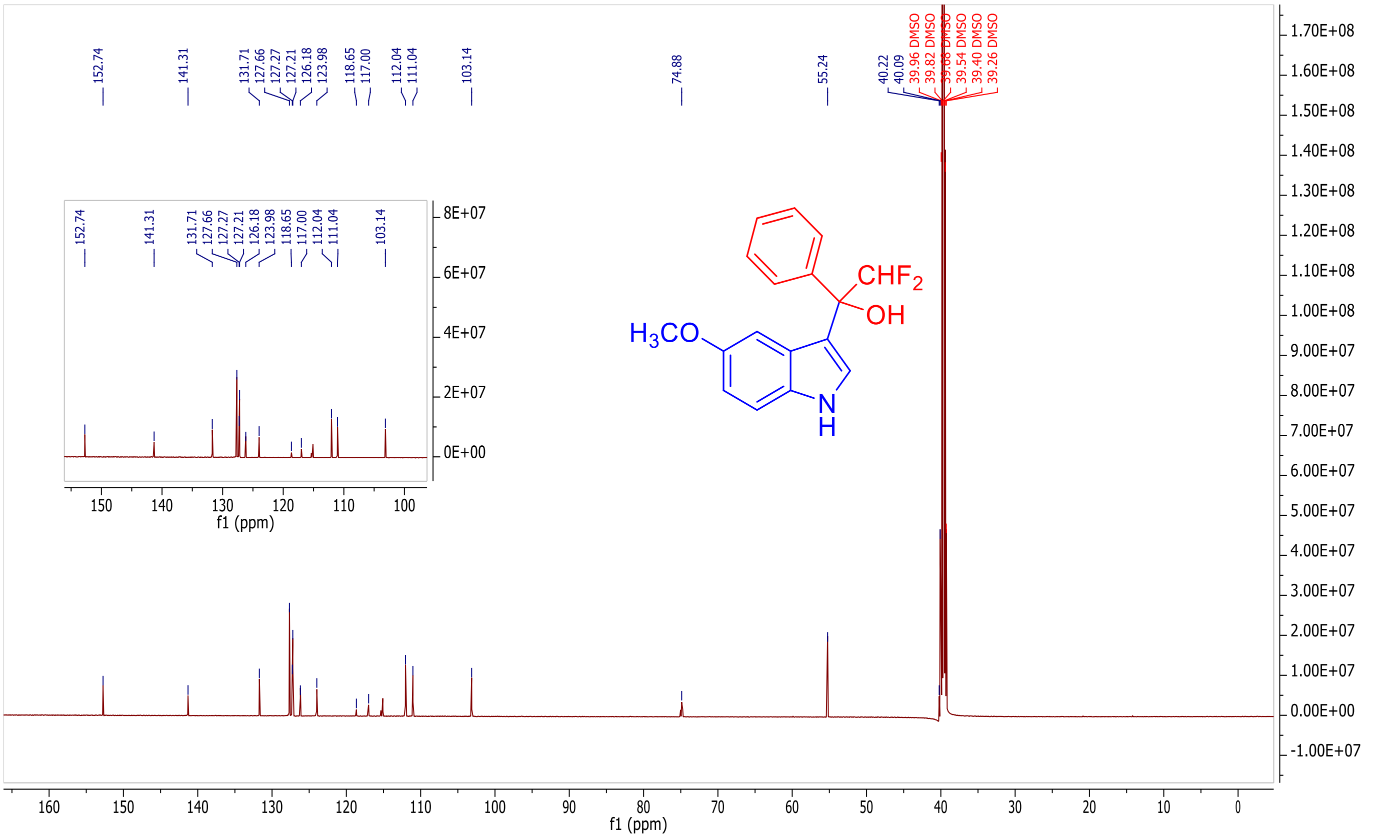

Figure S10. ^{1}H (600 MHz) and ^{13}C (151 MHz) Spectra of 2,2-tifluoro-1-(5-methoxy-1*H*-indol-3-yl)-1-phenylethan-1-ol (**3i**)

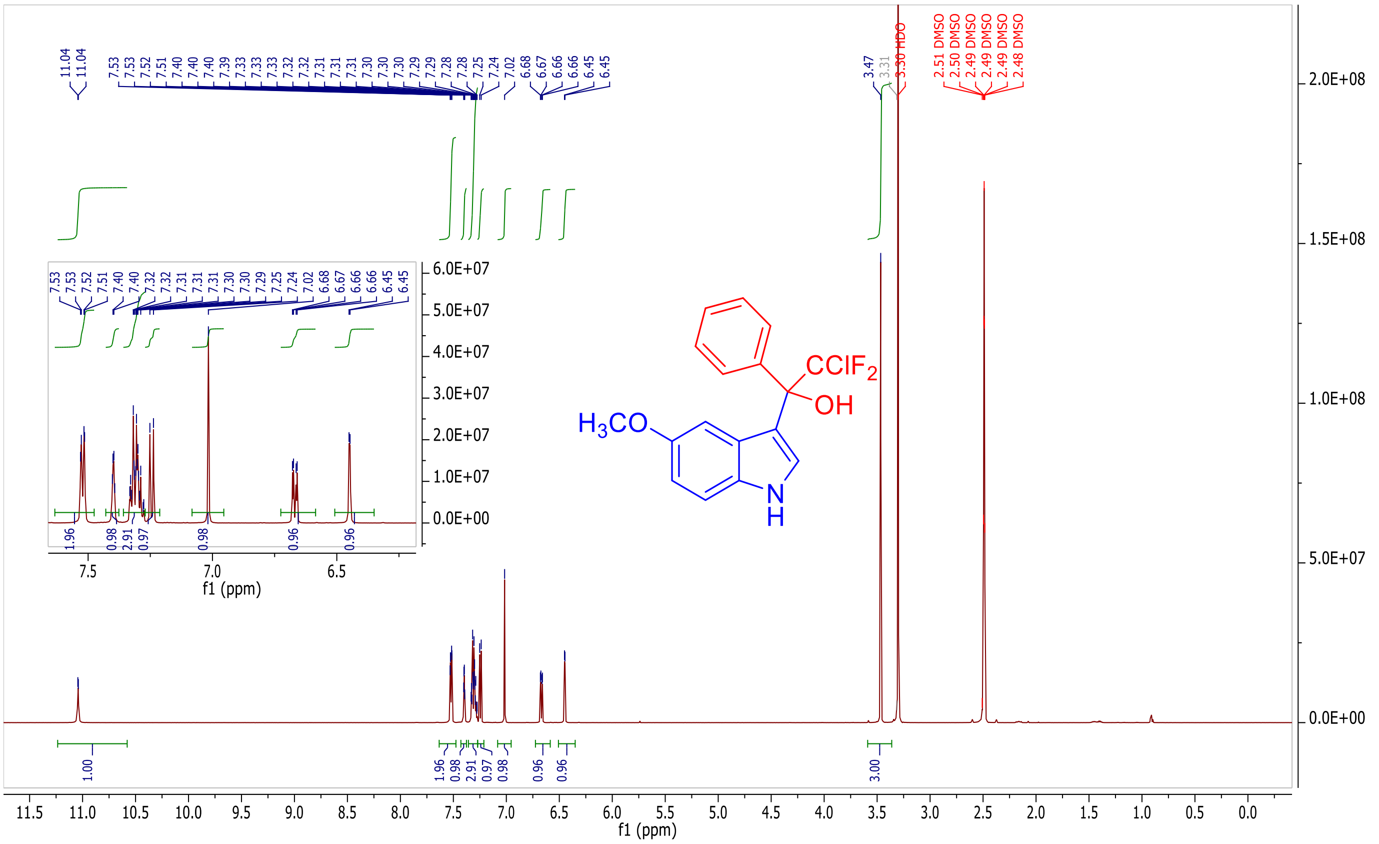

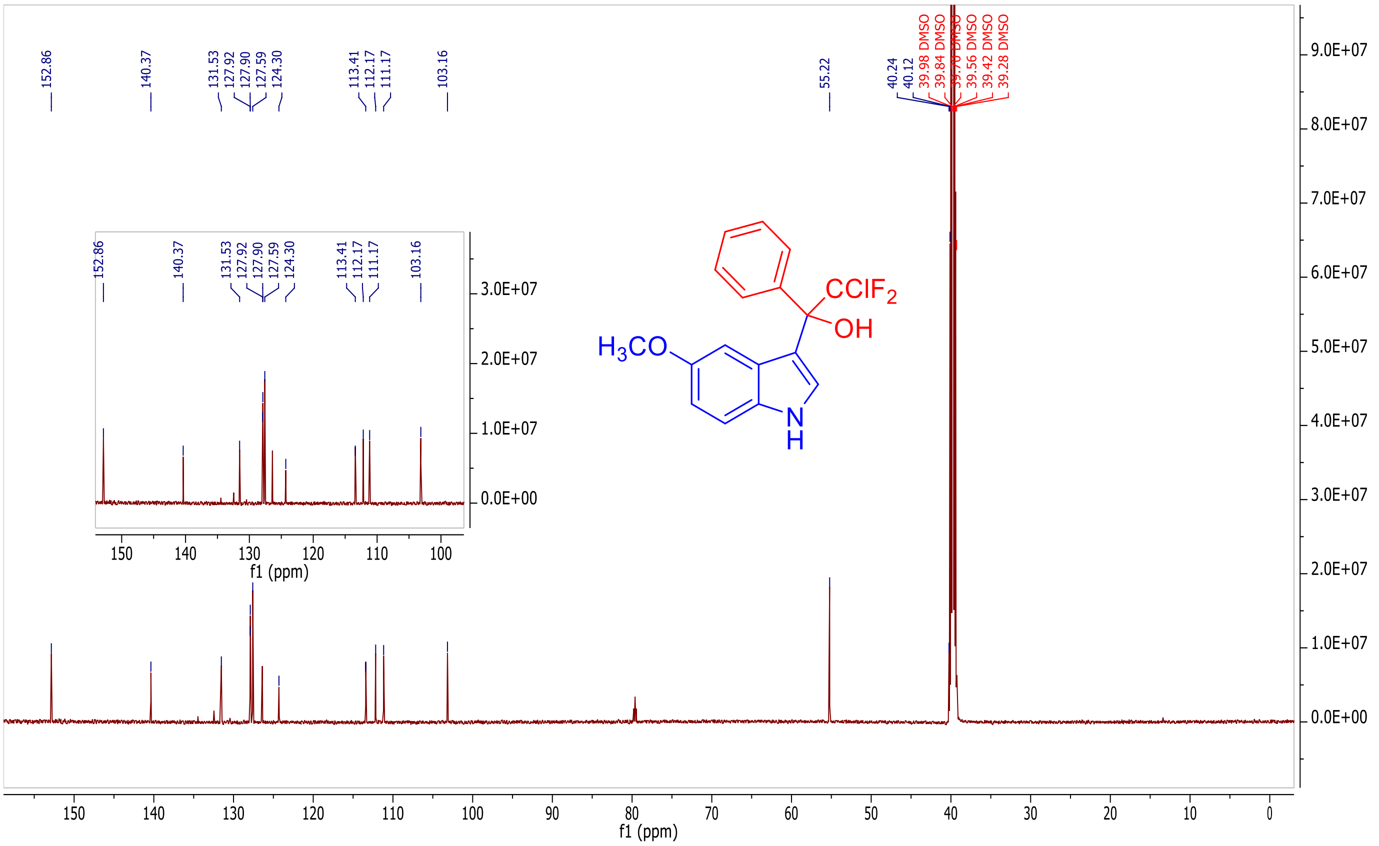

Figure S11. ^{1}H (600 MHz) and ^{13}C (151 MHz) Spectra of 2-chloro-2,2-difluoro-1-(5-methoxy-1*H*-indol-3-yl)-1-phenylethan-1-ol (**3m**)

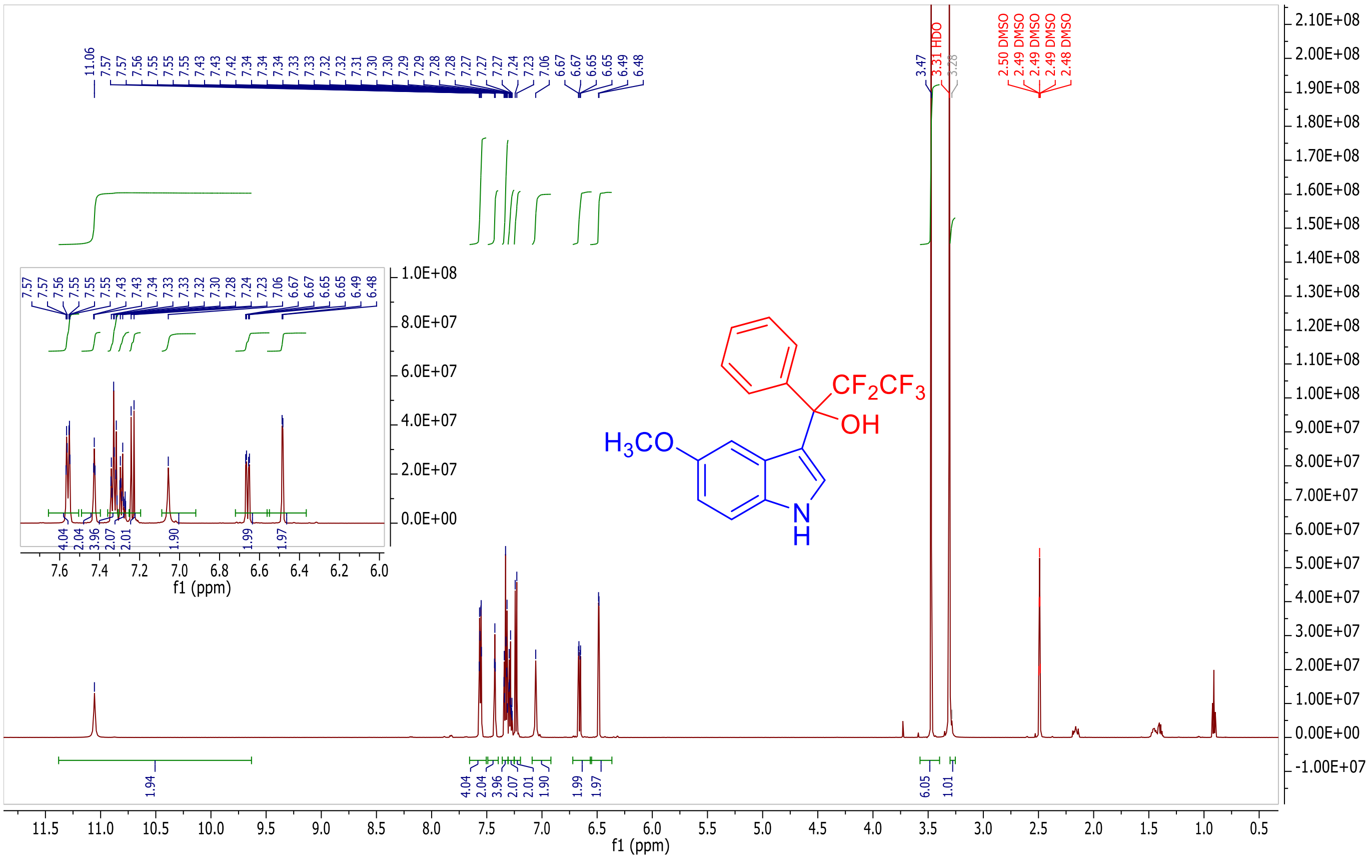

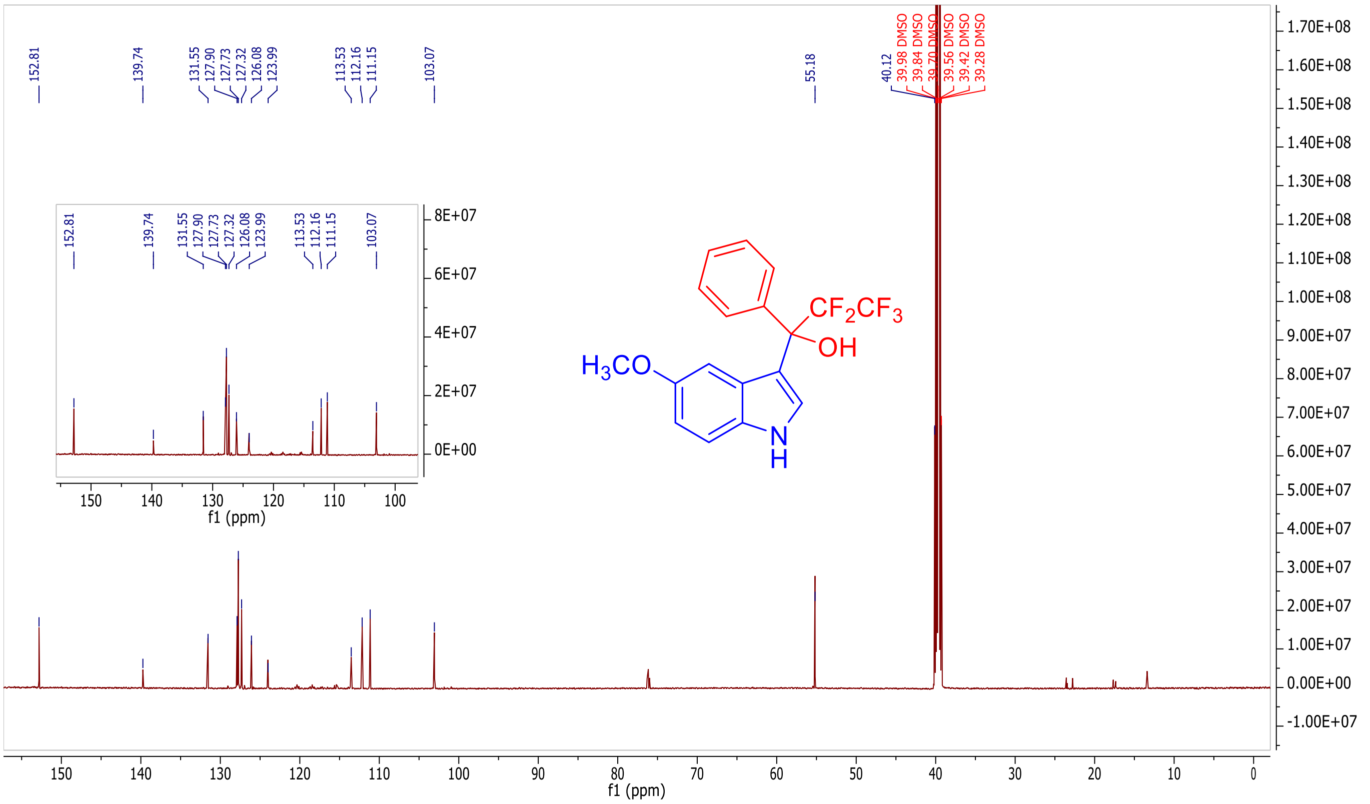

Figure S12. ^{1}H (600 MHz) and ^{13}C (151 MHz) Spectra of 2,2,3,3,3-pentafluoro-1-(5 methoxy-1*H*-indol-3-yl)-1-phenylpropan-1-ol (**3n**)

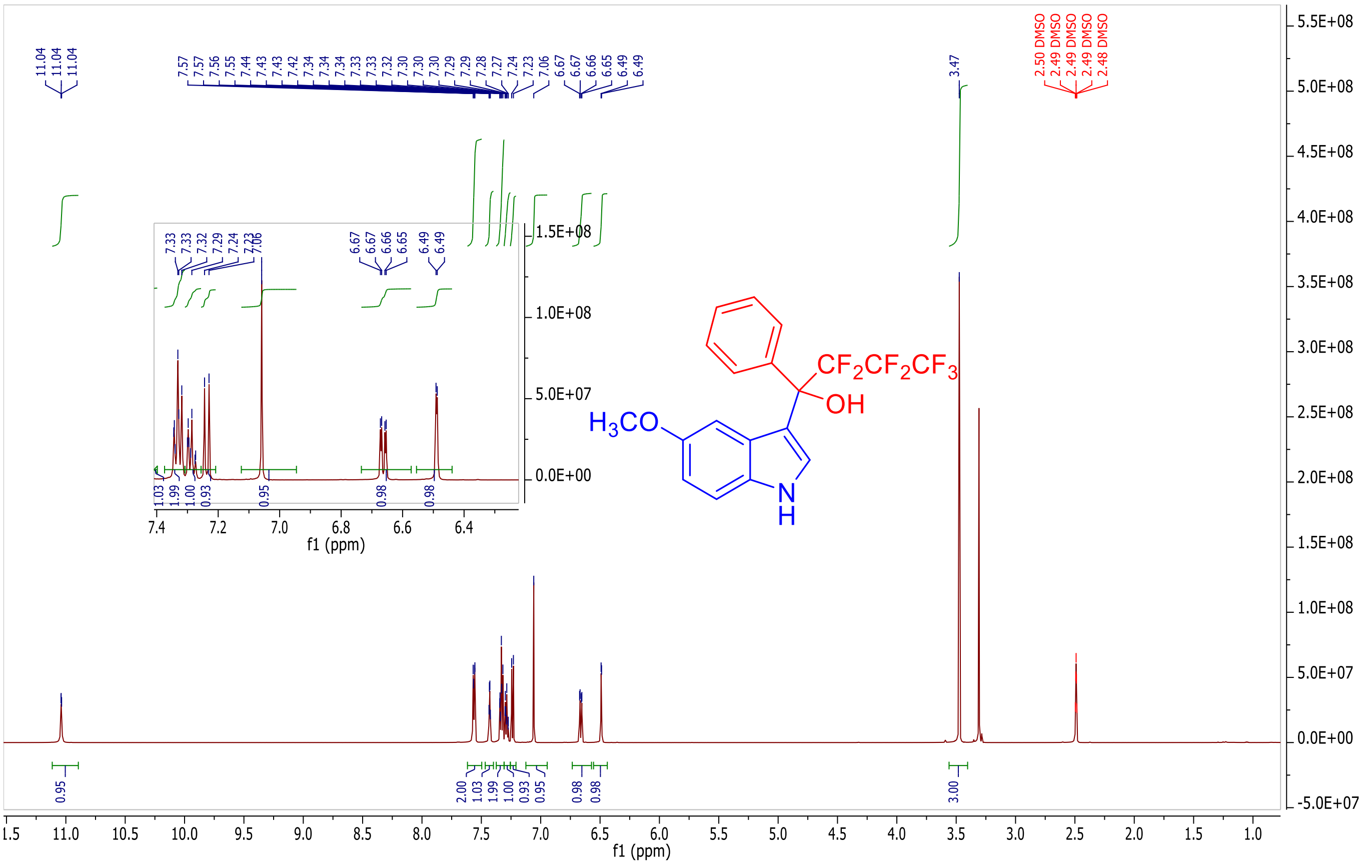

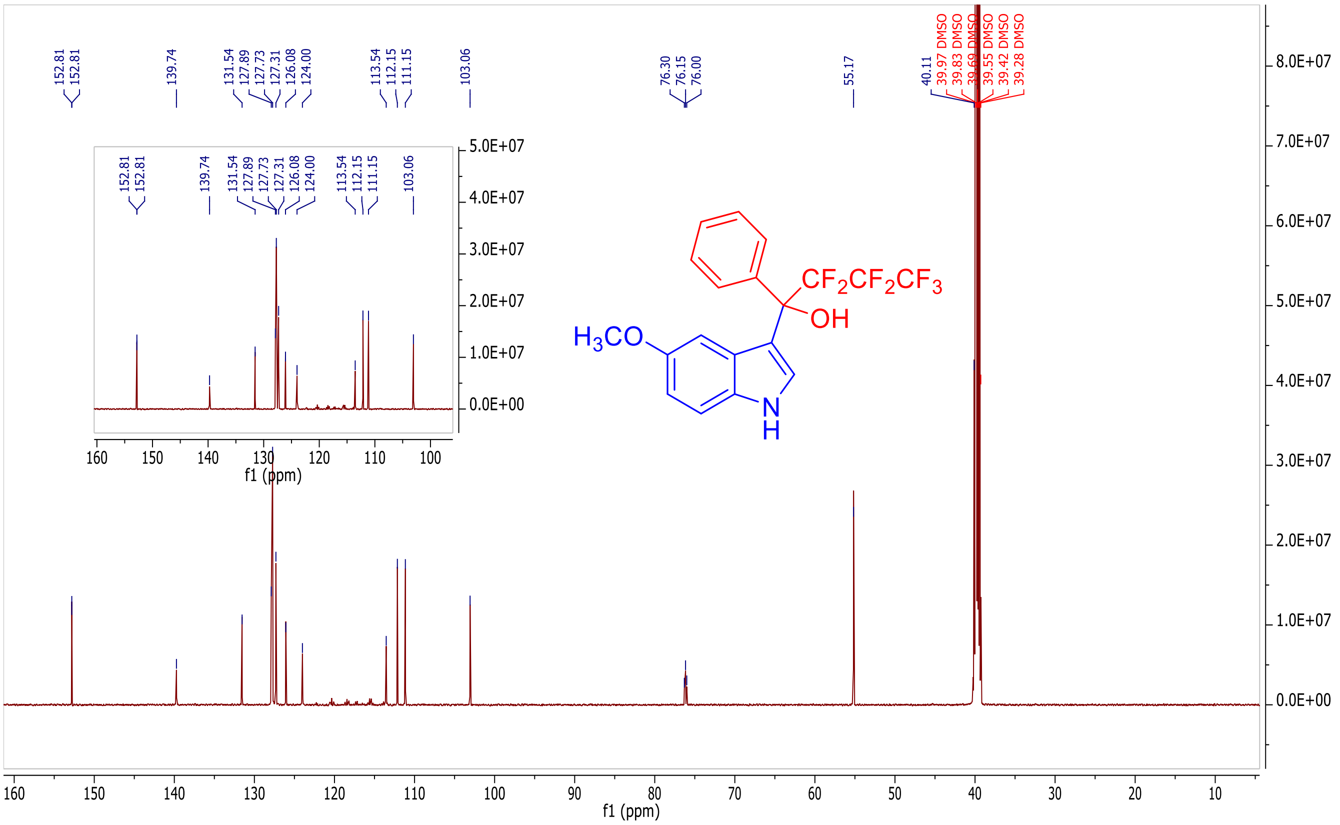

Figure S13. ^{1}H (600 MHz) and ^{13}C (151 MHz) Spectra of 2,2,3,3,4,4,4-heptafluoro-1-(5-methoxy-1*H*-indol-3-yl)-1-phenylbutan-1-ol (**3o**)

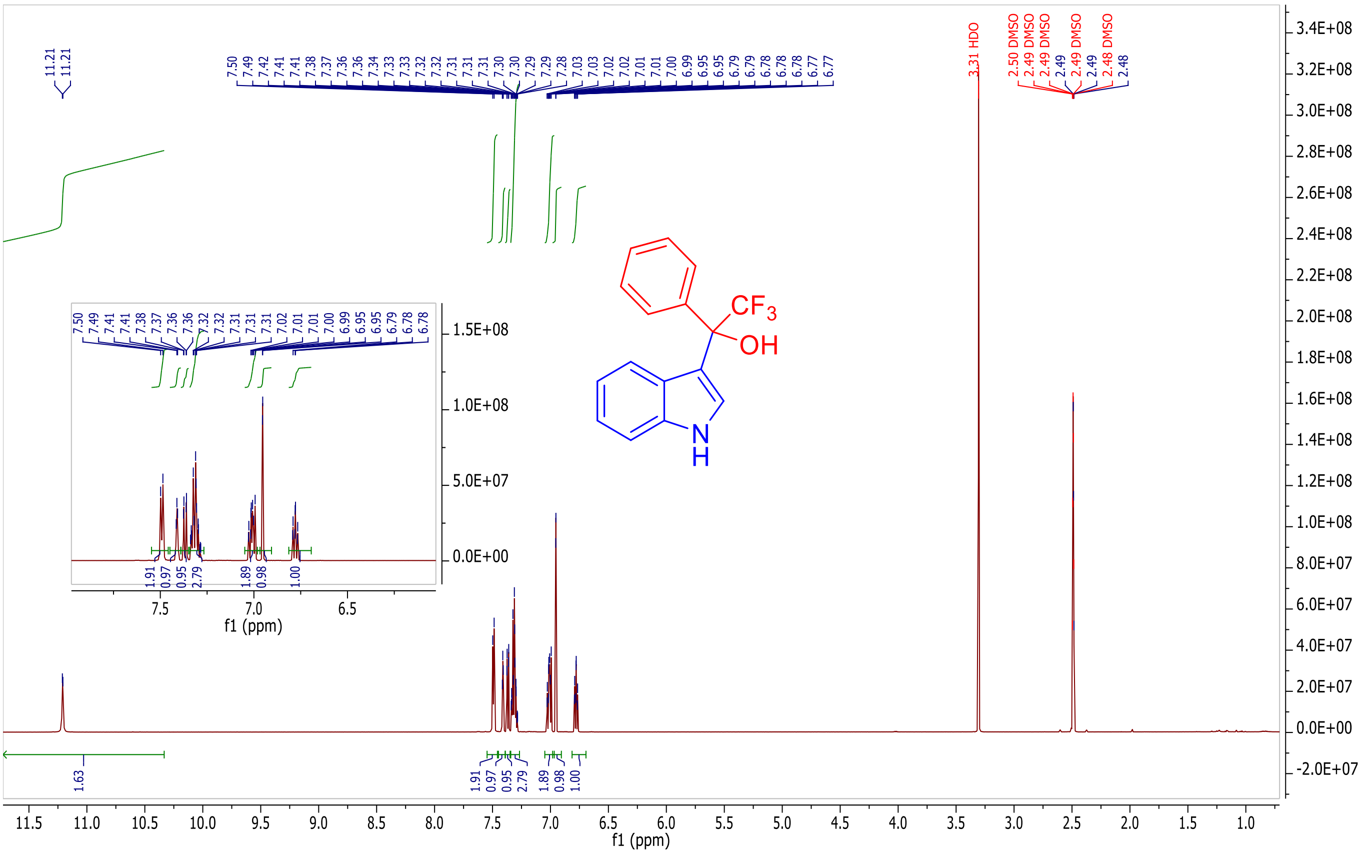

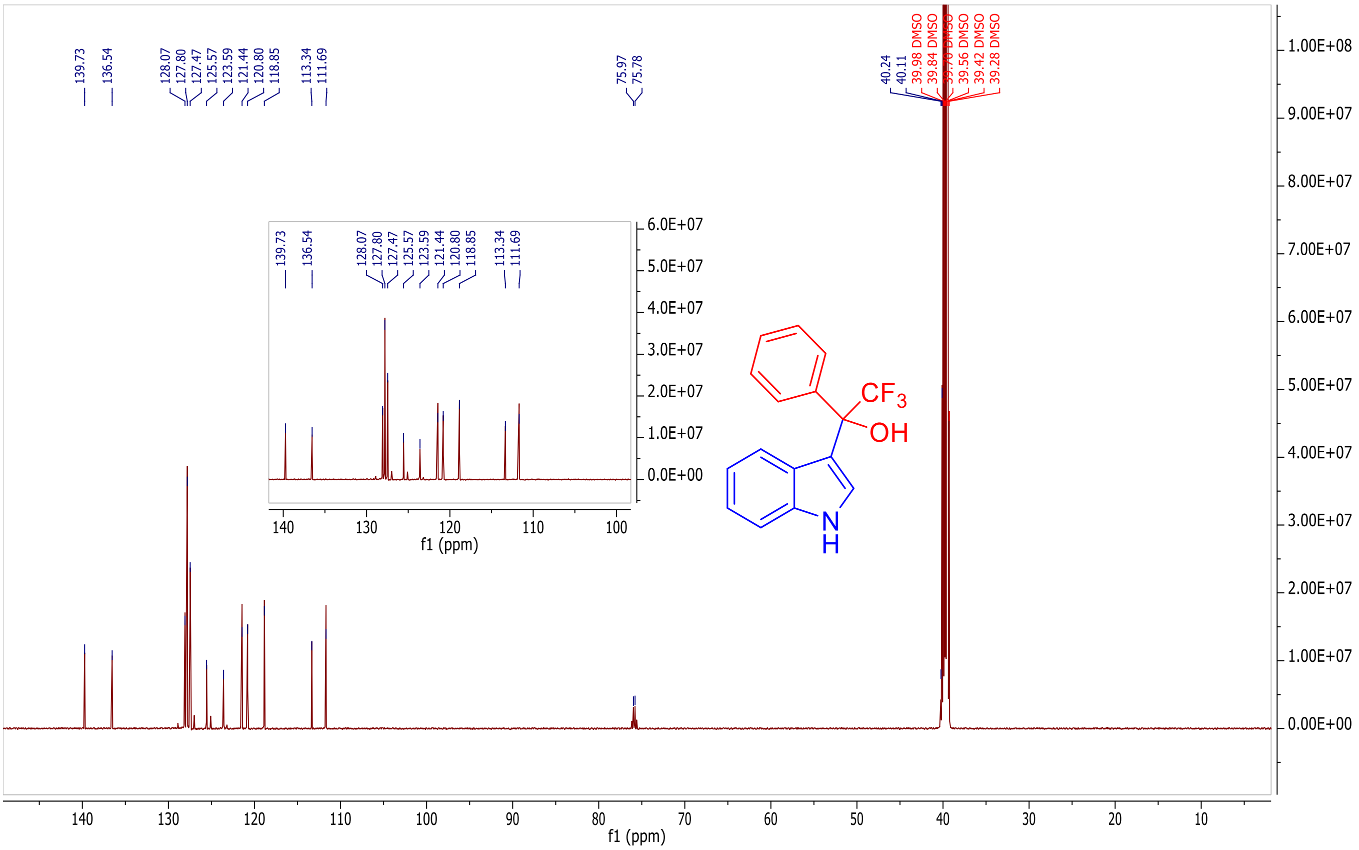

Figure S14. ^{1}H (600 MHz) and ^{13}C (151 MHz) Spectra of 2,2,2-trifluoro-1-(1*H*-indol-3-yl)-1-phenylethan-1-ol (**3p**)

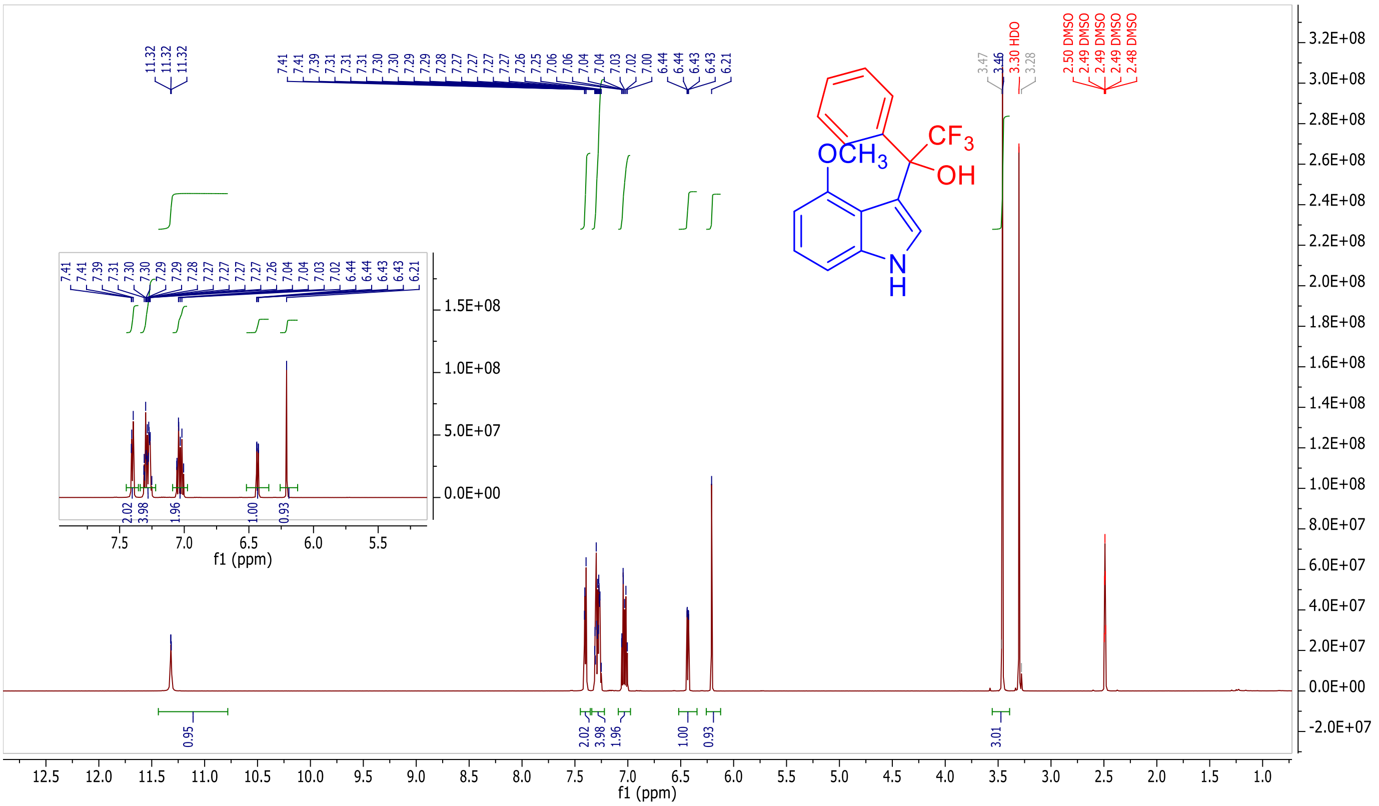

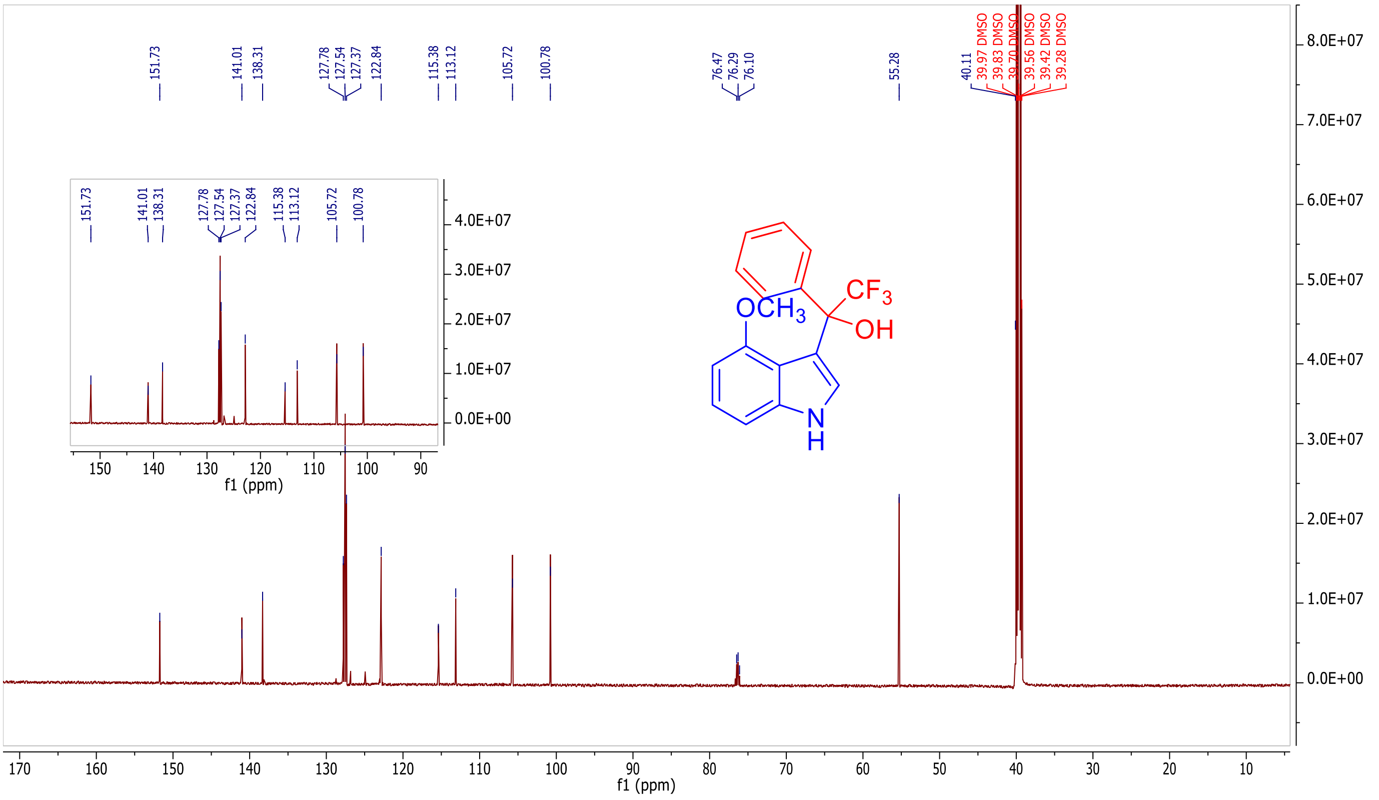

Figure S15. ^{1}H (600 MHz) and ^{13}C (151 MHz) Spectra of 2,2,2-trifluoro-1-(4-methoxy-1*H*-indol-3-yl)-1-phenylethan-1-ol (**3q**)

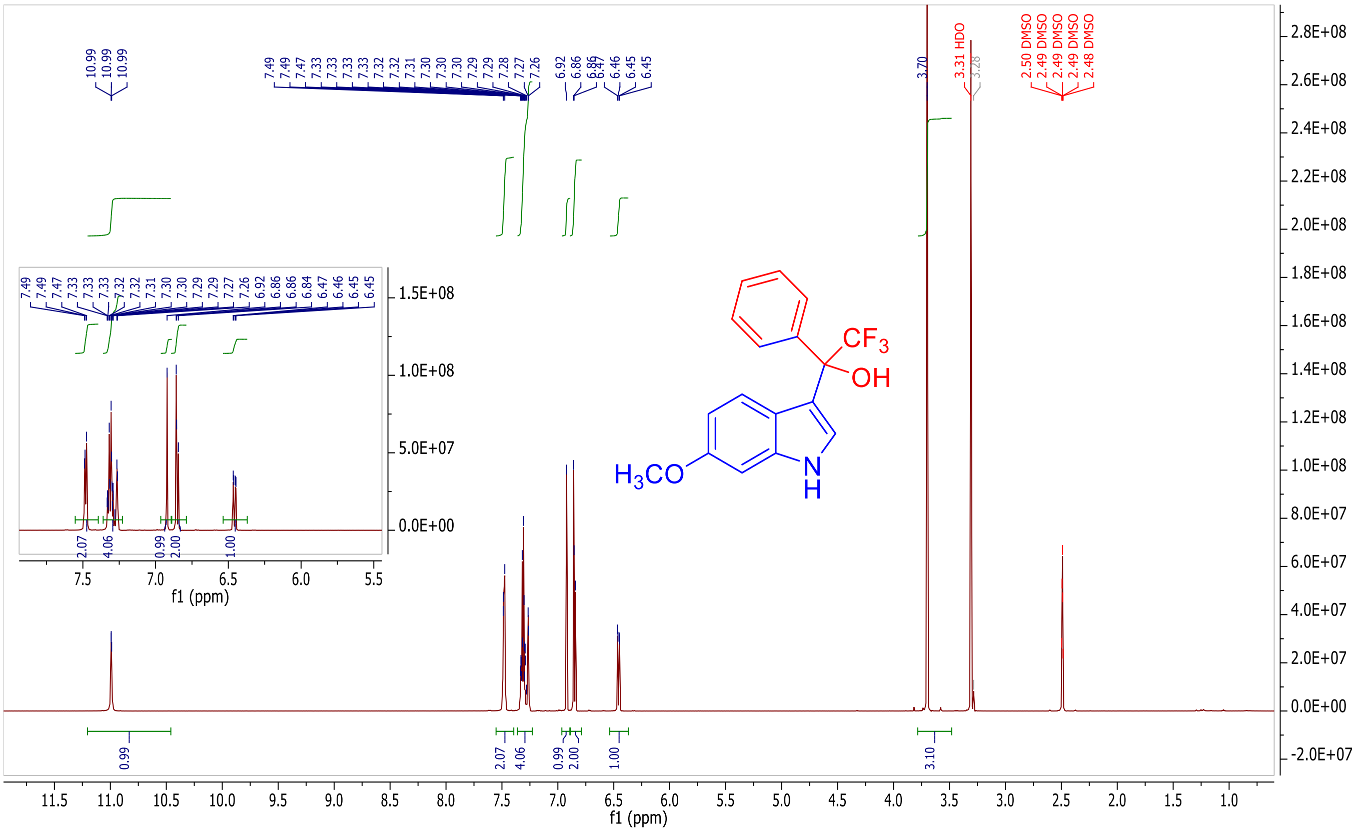

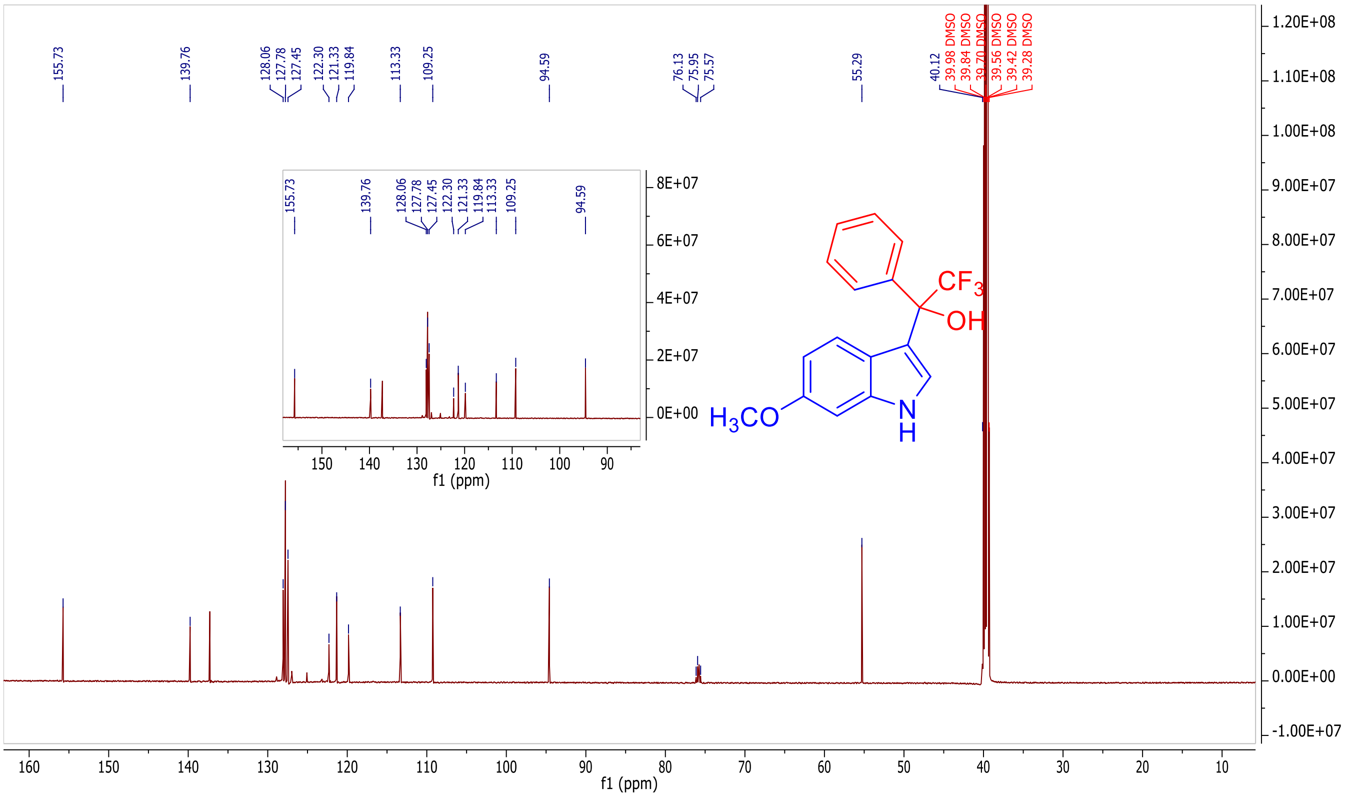

Figure S16. ^{1}H (600 MHz) and ^{13}C (151 MHz) Spectra of 2,2,2-trifluoro-1-(6-methoxy-1*H*-indol-3-yl)-1-phenylethan-1-ol (**3r**)

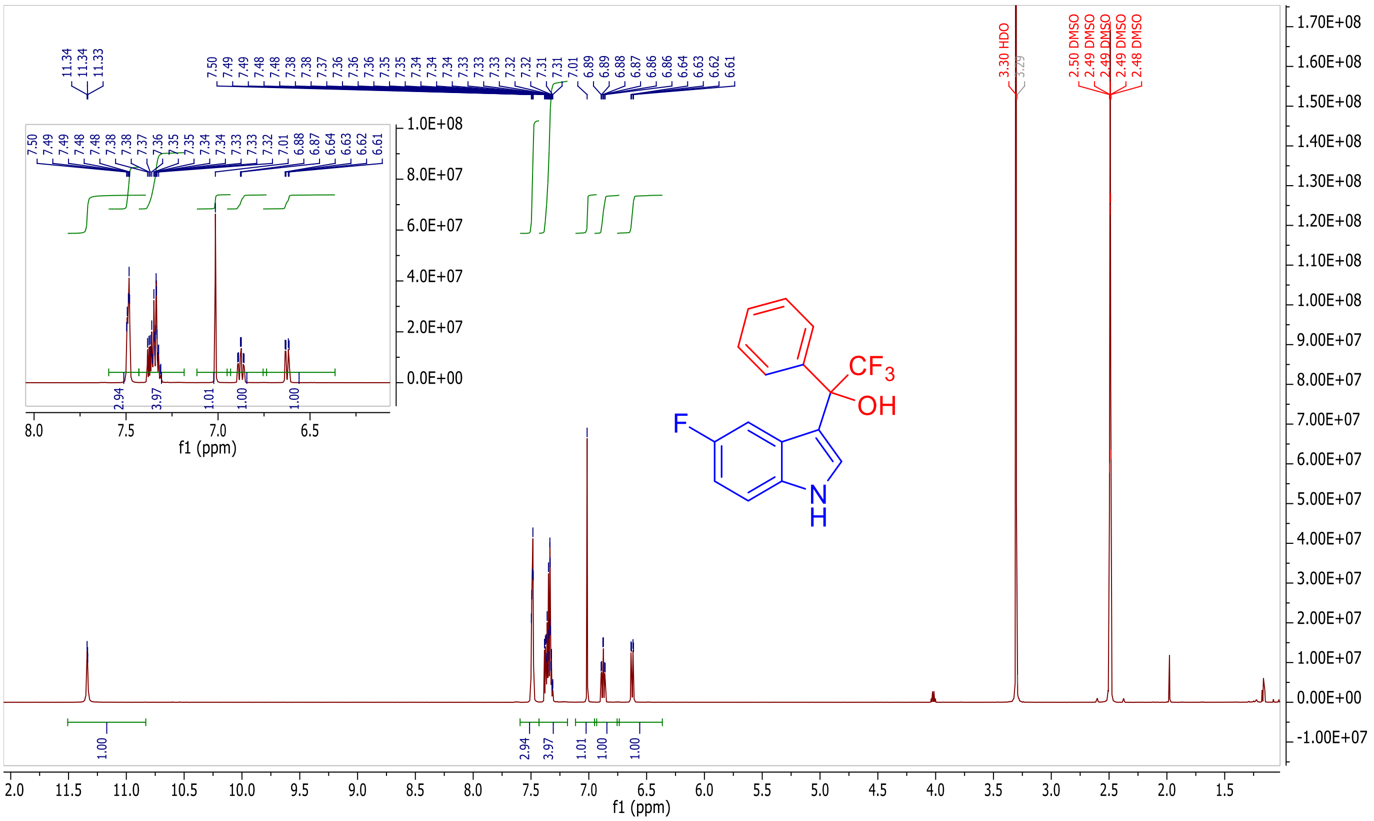

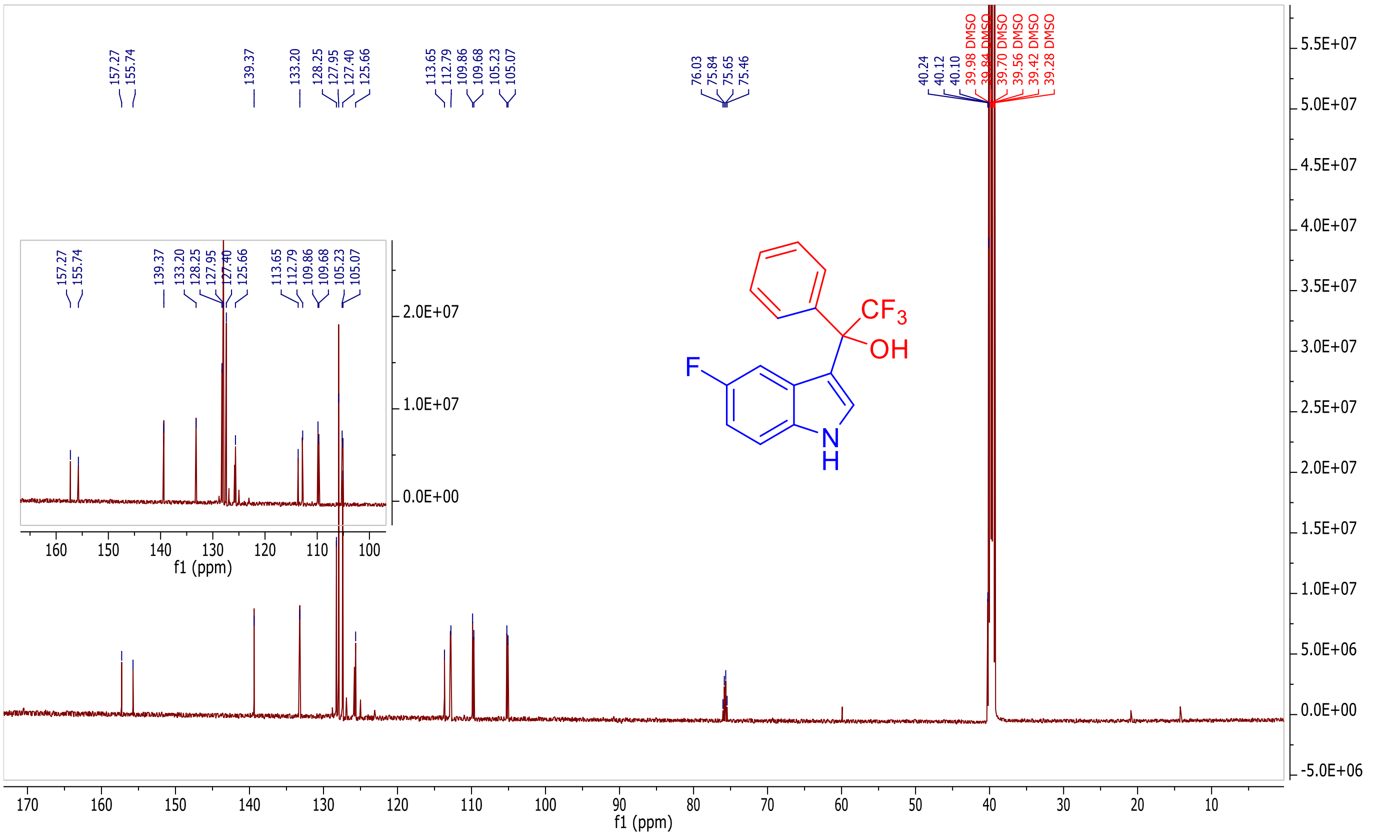

Figure S17. ^{1}H (600 MHz) and ^{13}C (151 MHz) Spectra of 2,2,2-trifluoro-1-(5-fluoro-1*H*-indol-3-yl)-1-phenylethan-1-ol (**3s**)

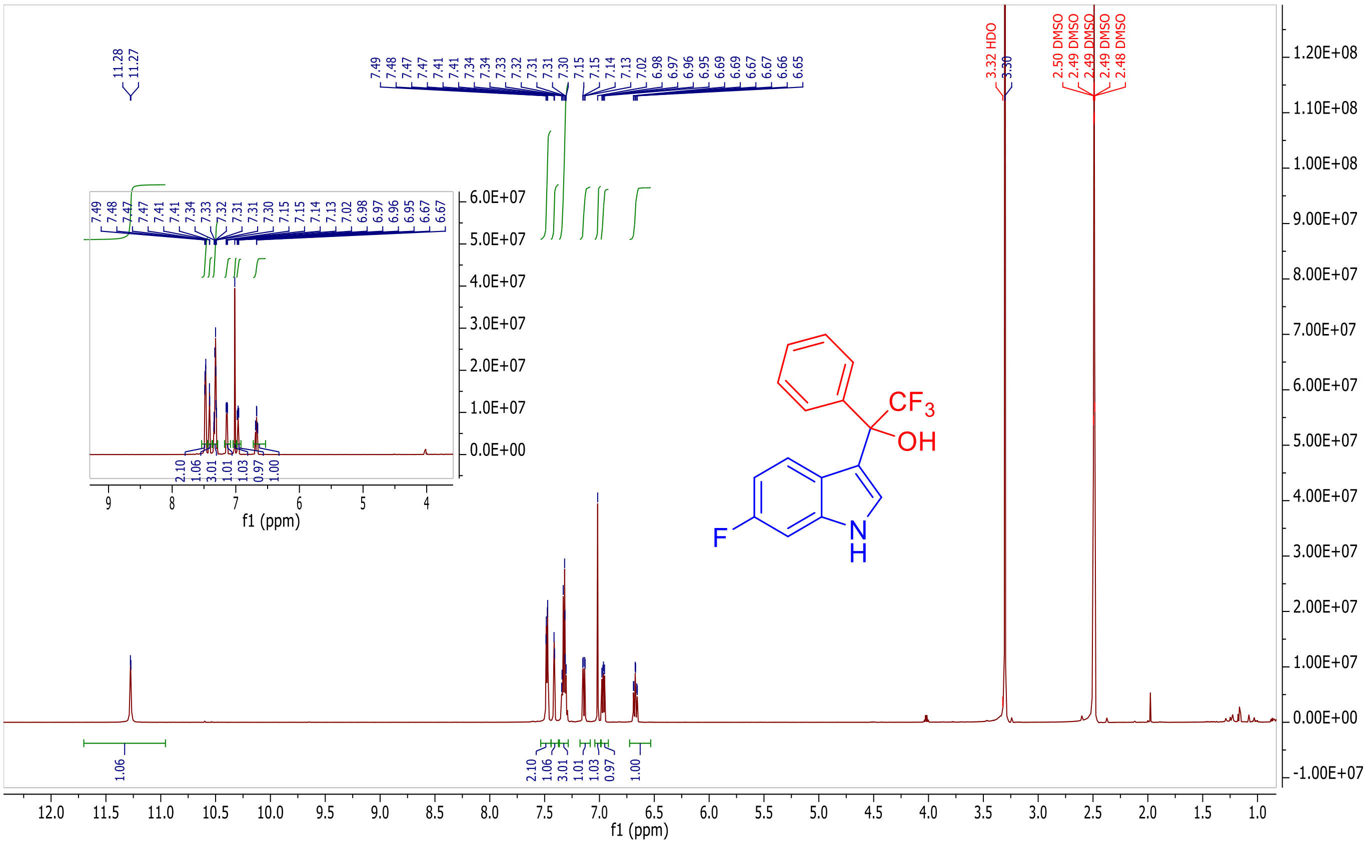

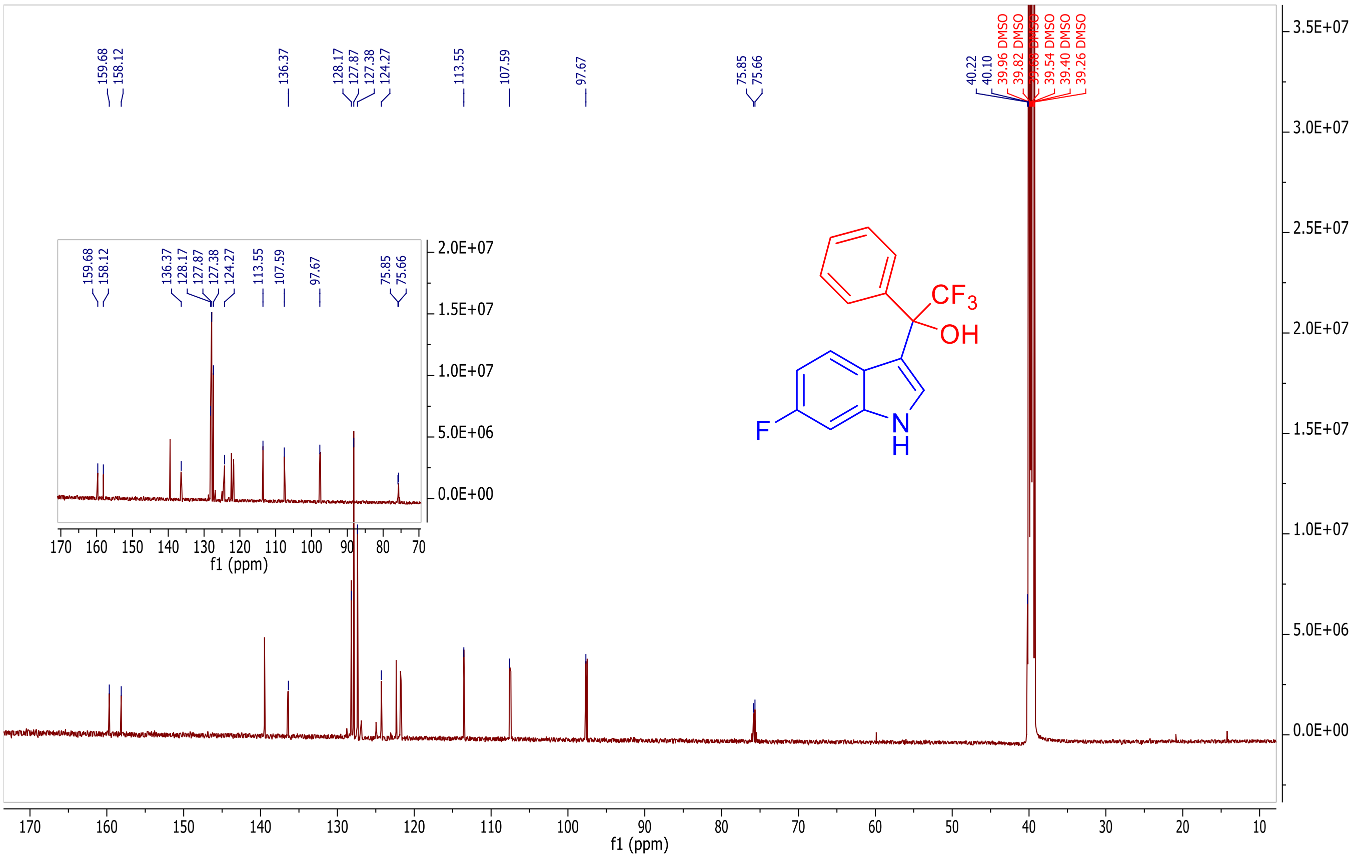

Figure S18. ^{1}H (600 MHz) and ^{13}C (151 MHz) Spectra of 2,2,2-trifluoro-1-(6-fluoro-1*H*-indol-3-yl)-1-phenylethan-1-ol (**3t**)

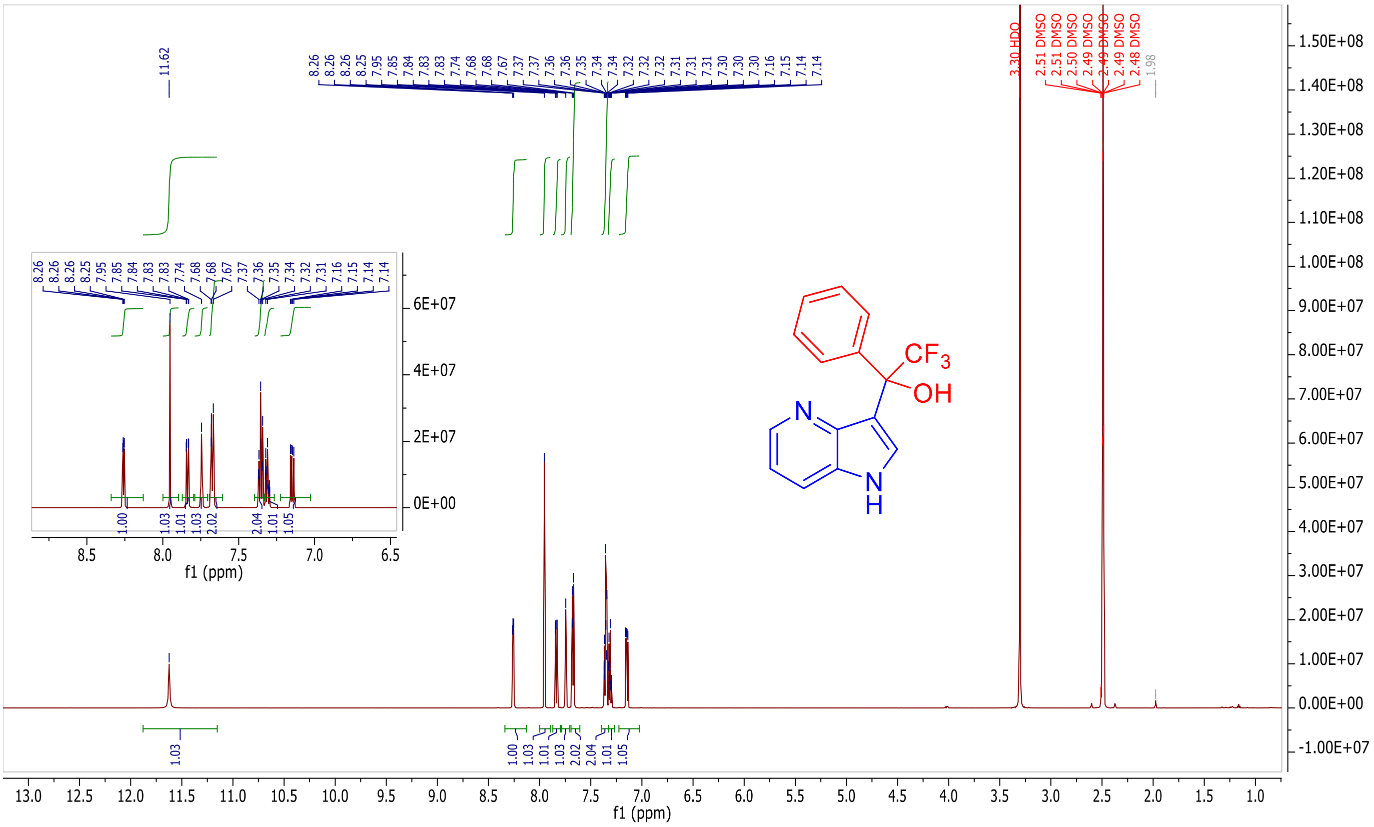

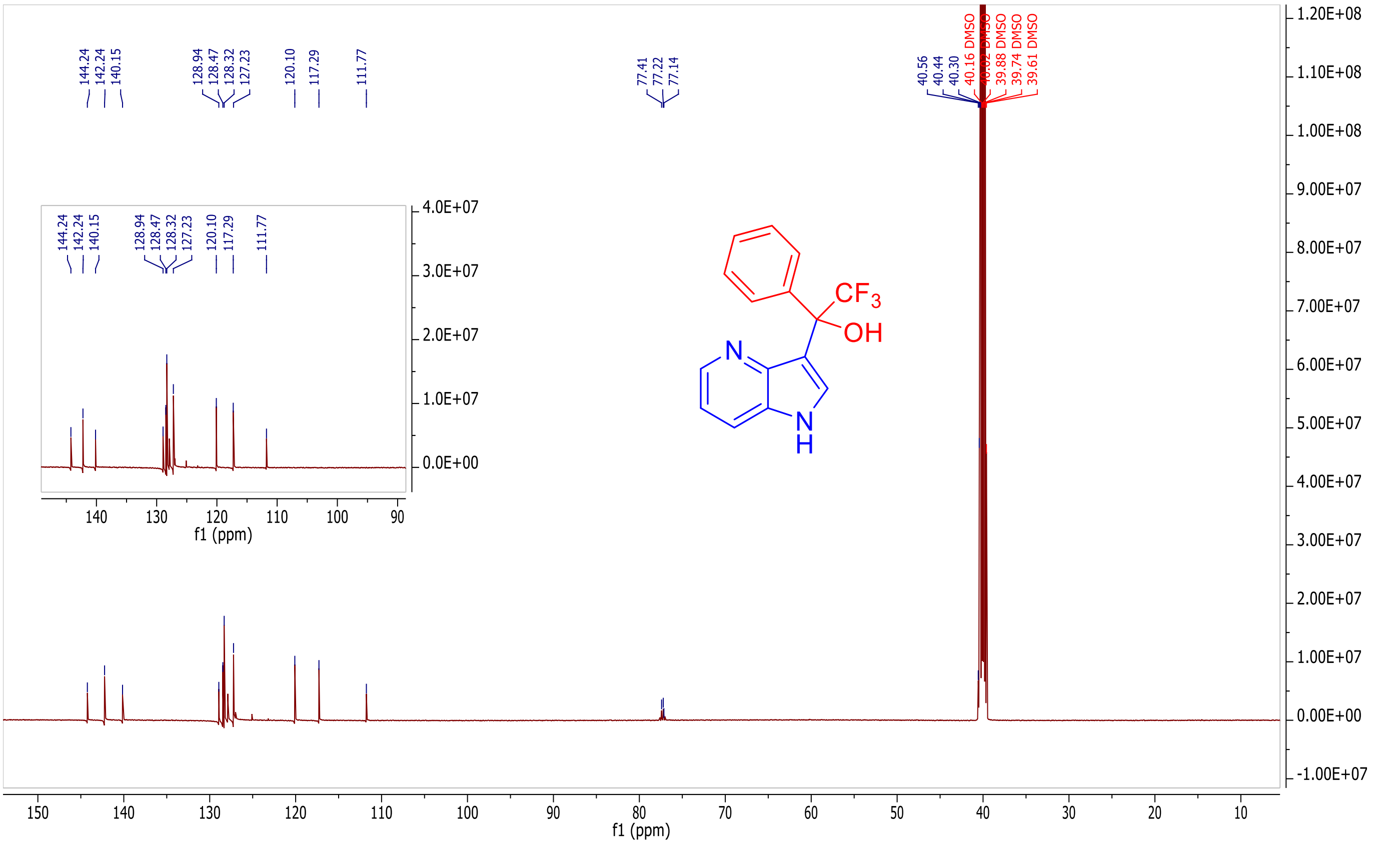

Figure S19. ^{1}H (600 MHz) and ^{13}C (151 MHz) Spectra of 2,2,2-trifluoro-1-phenyl-1-(1*H*-pyrrolo[3,2-*b*]pyridin-3-yl)ethan-1-ol (**3u**)

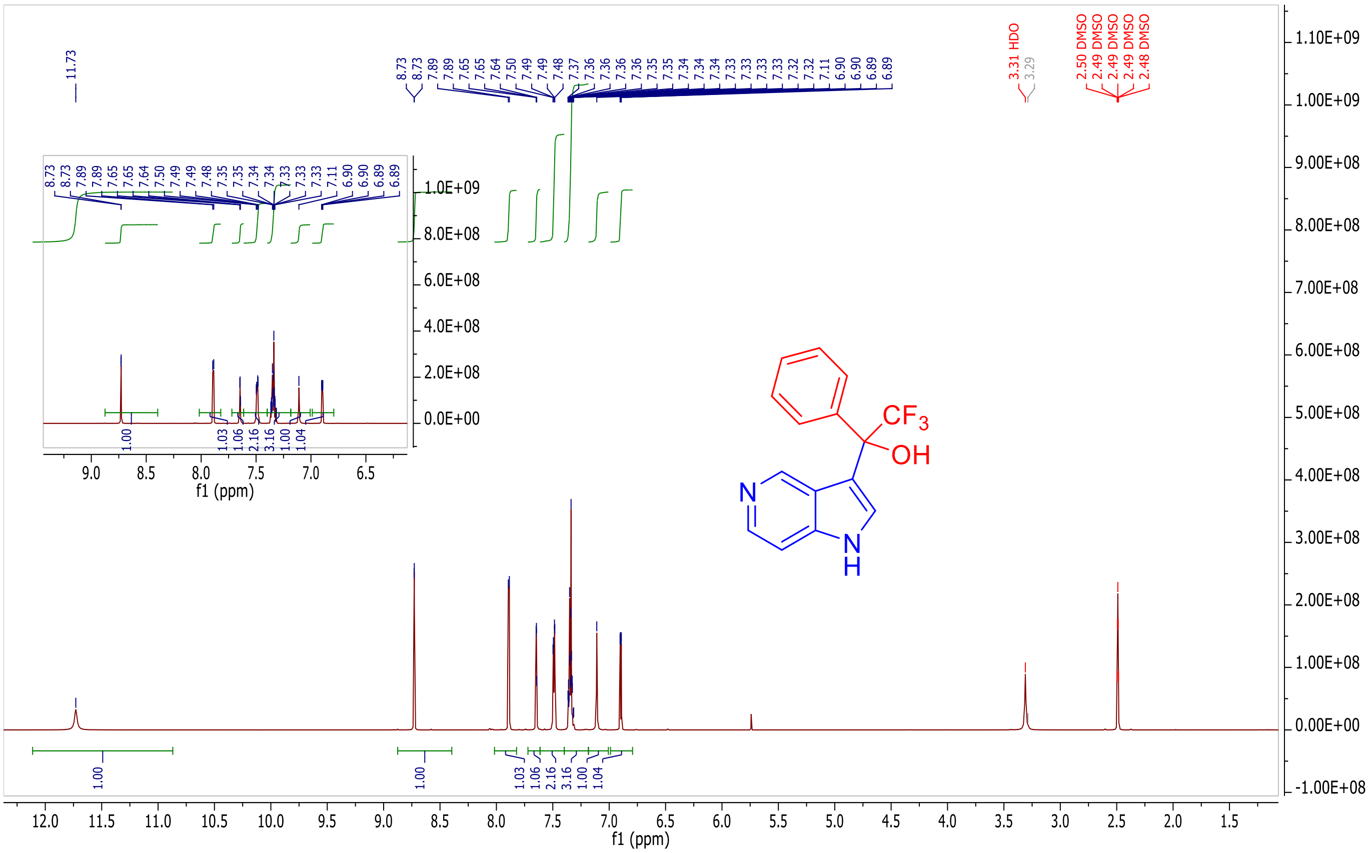

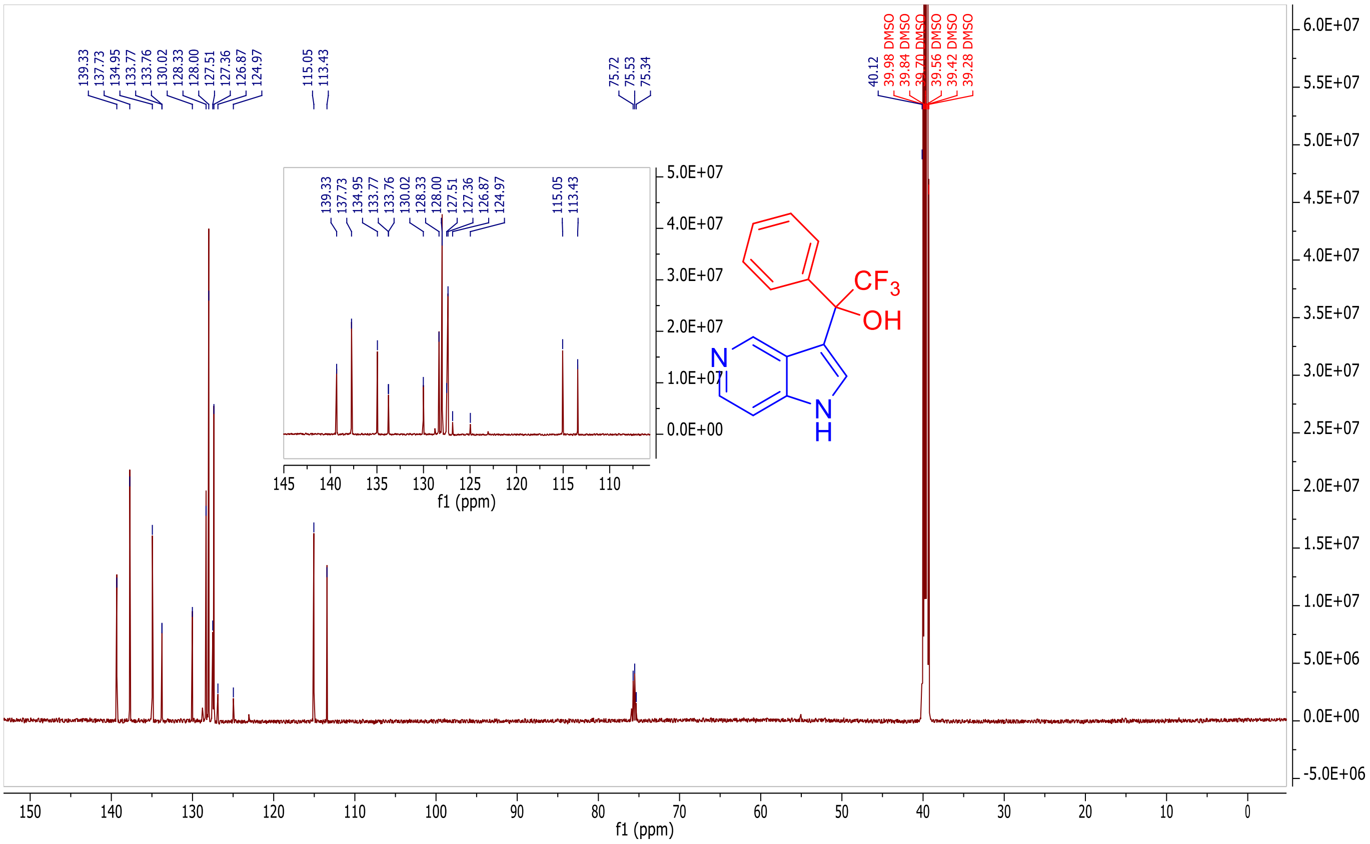

Figure S20. ^{1}H (600 MHz) and ^{13}C (151 MHz) Spectra of 2,2,2-Trifluoro-1-phenyl-1-(1*H*-pyrrolo[3,2-*c*]pyridin-3-yl)ethan-1-ol (**3v**)

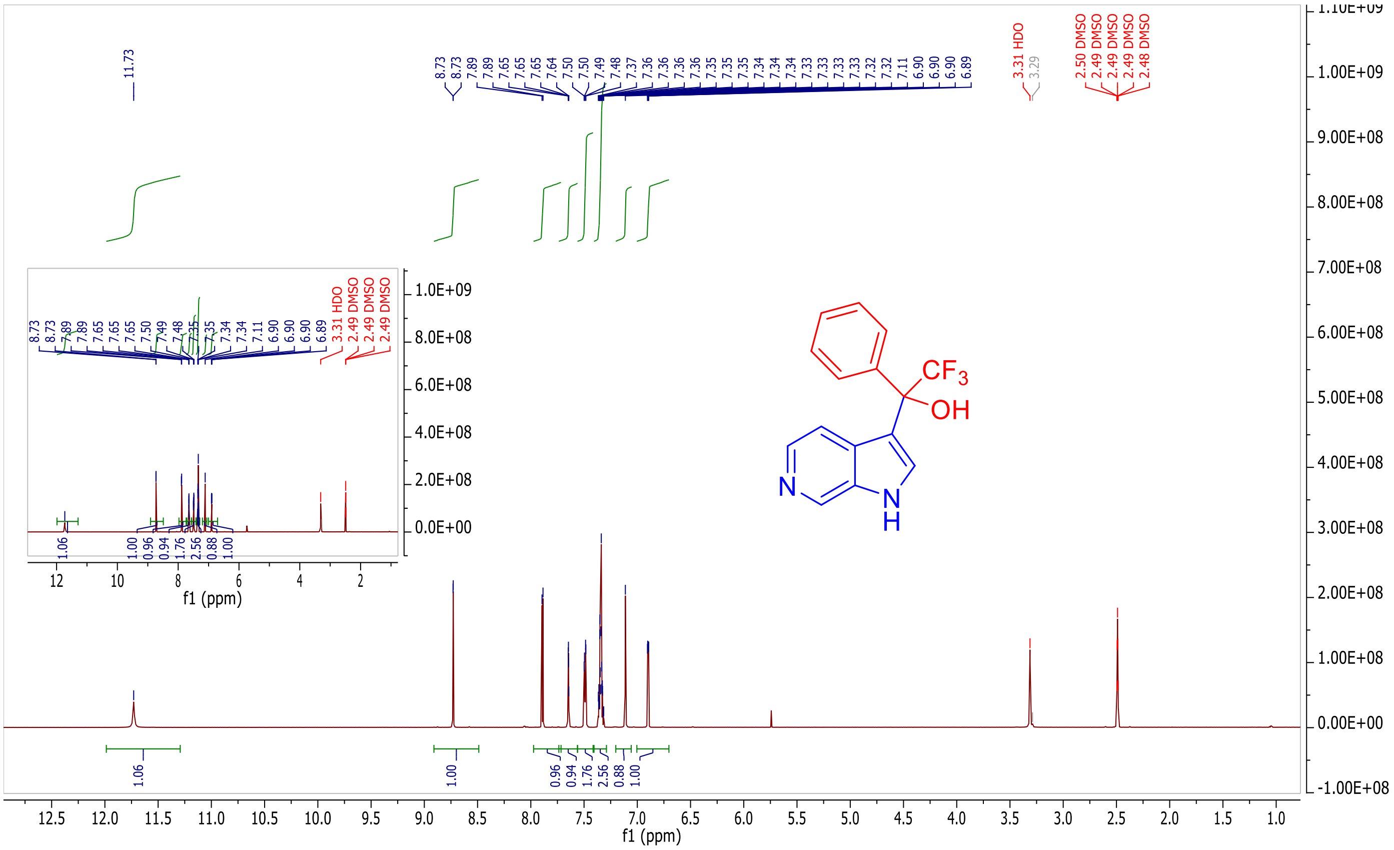

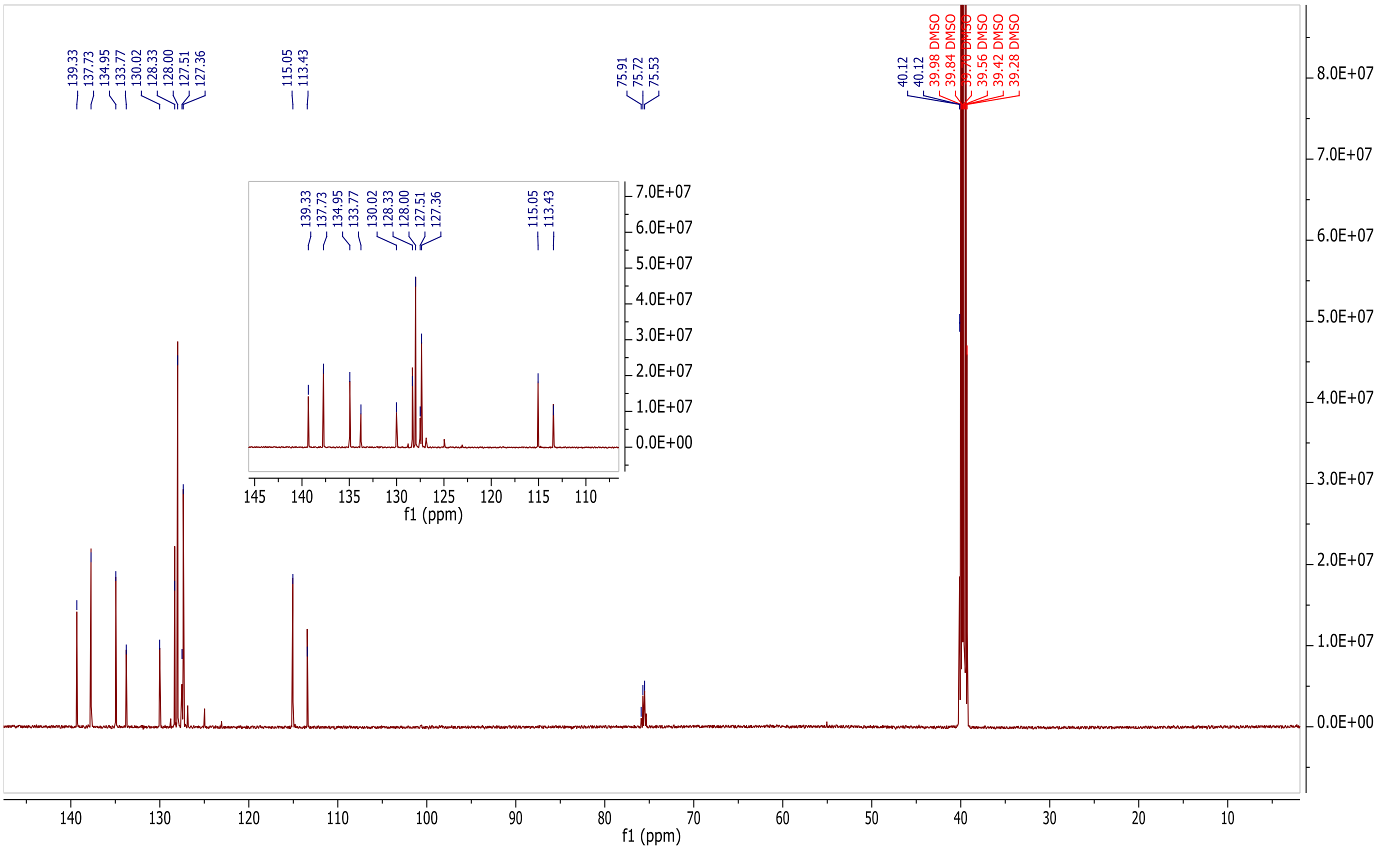

Figure S21. ^{1}H (600 MHz) and ^{13}C (151 MHz) Spectra of 2,2,2-trifluoro-1-phenyl-1-(1H-pyrrolo[2,3-c]pyridin-3-yl)ethan-1-ol (**3w**)

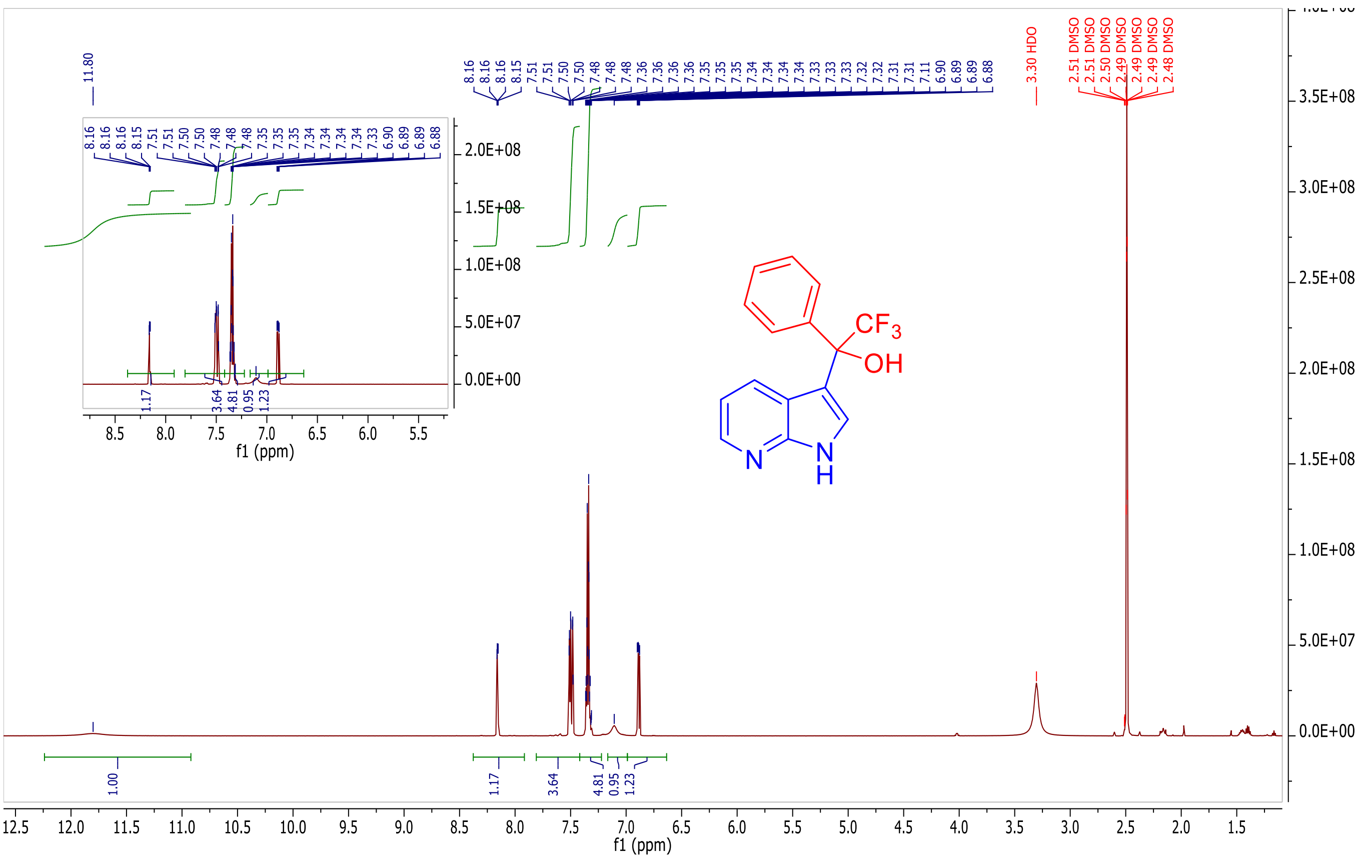

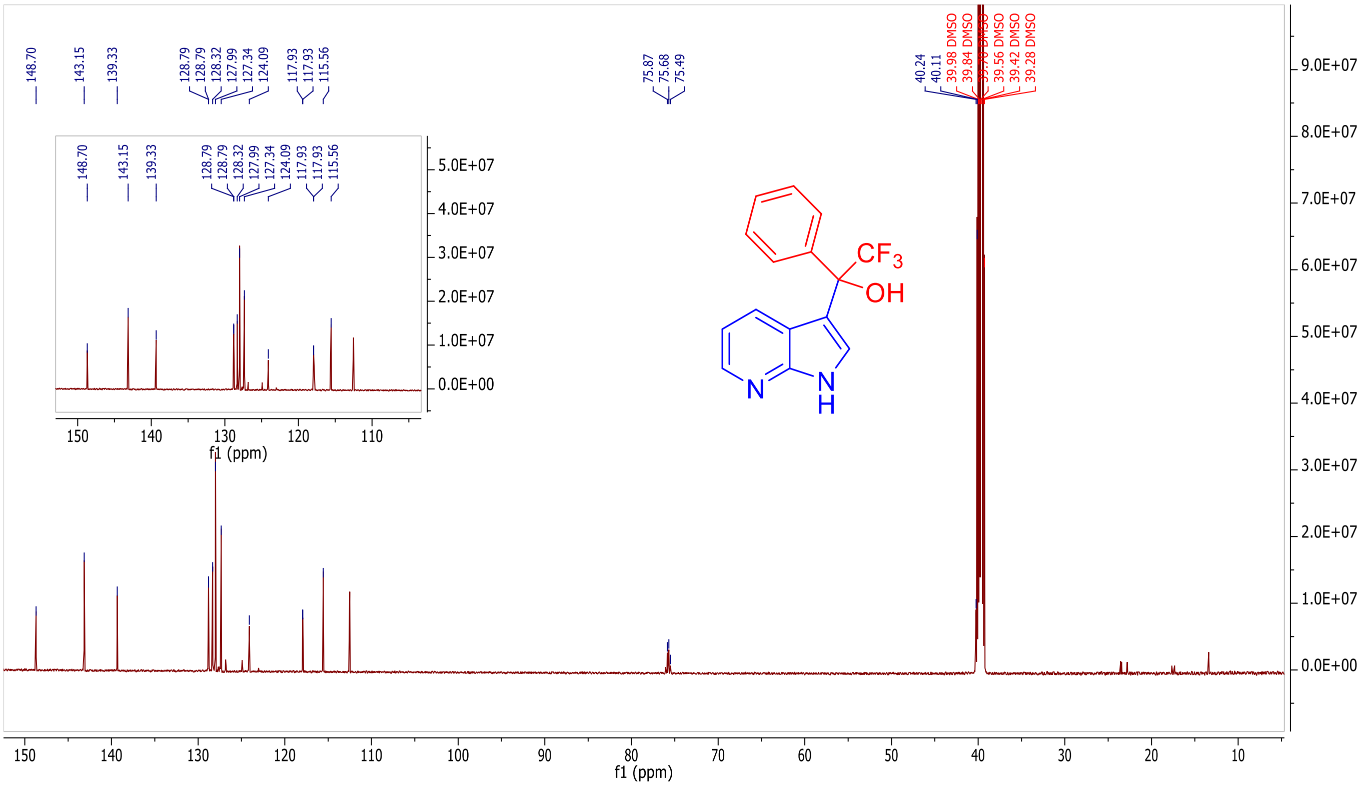

Figure S22. ^{1}H (600 MHz) and ^{13}C (151 MHz) Spectra of 2,2,2-trifluoro-1-phenyl-1-(1*H*-pyrrolo[2,3-*b*]pyridin-3-yl)ethan-1-ol (**3x**)

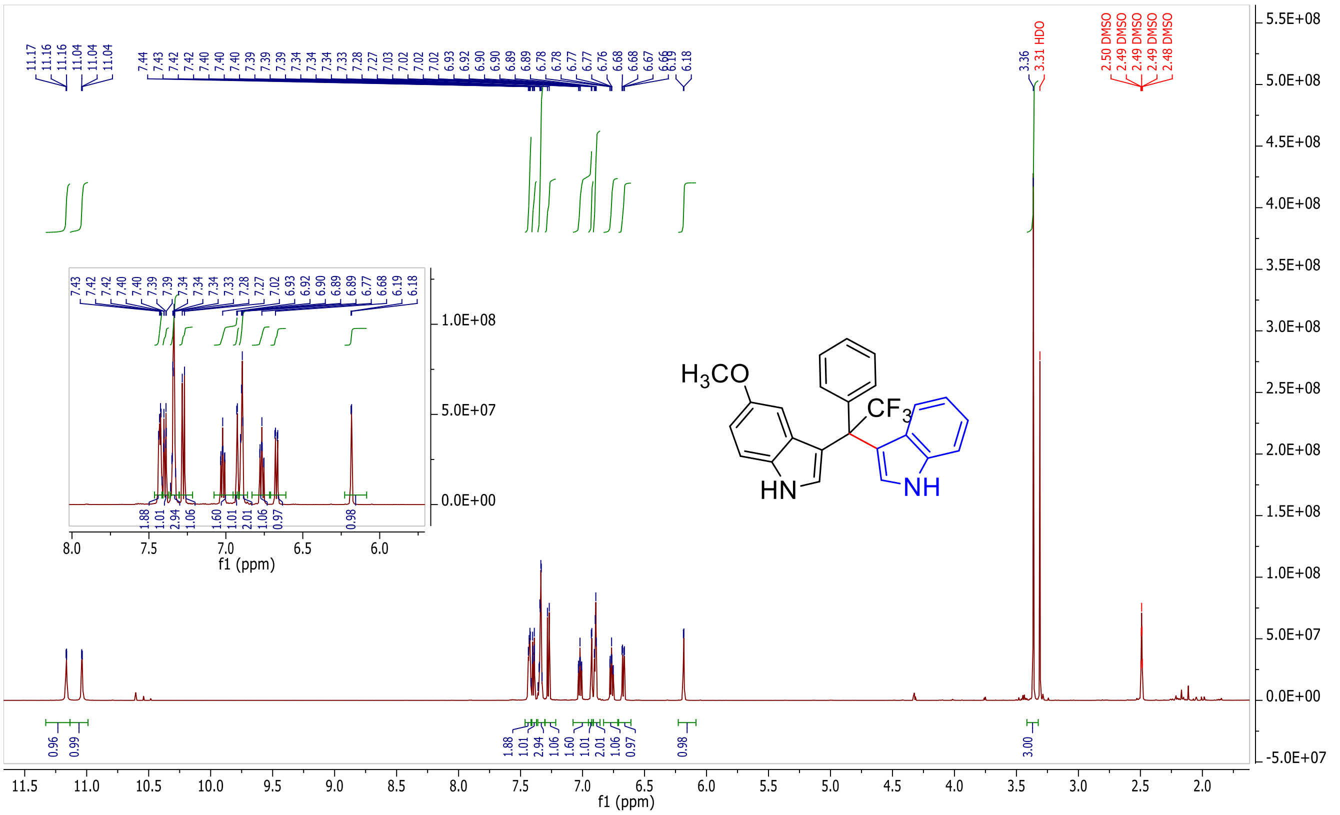

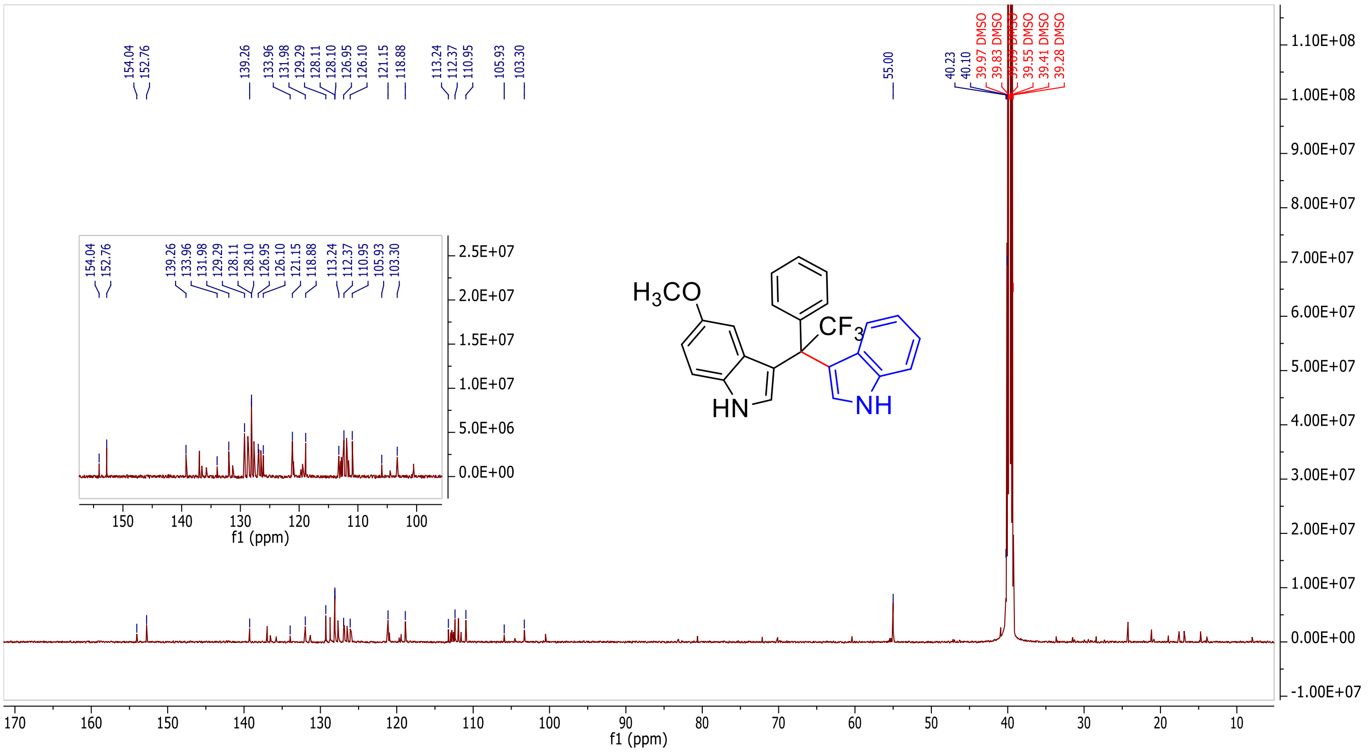

Figure S23. ^{1}H (600 MHz) and ^{13}C (151 MHz) Spectra of 5-methoxy-3-(2,2,2-trifluoro-1-(1*H*-indol-3-yl)-1-phenylethyl)-1*H*-indole (**9**)

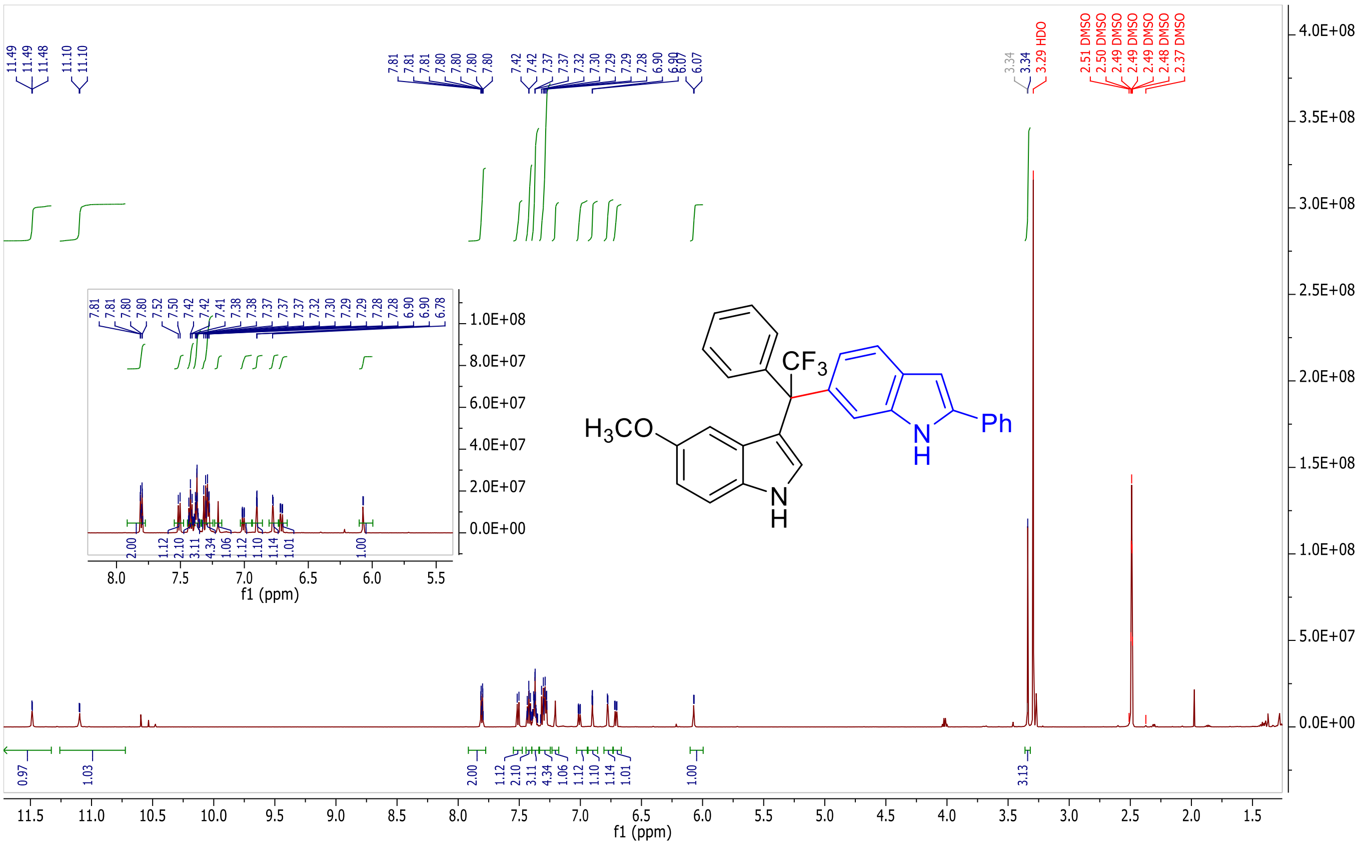

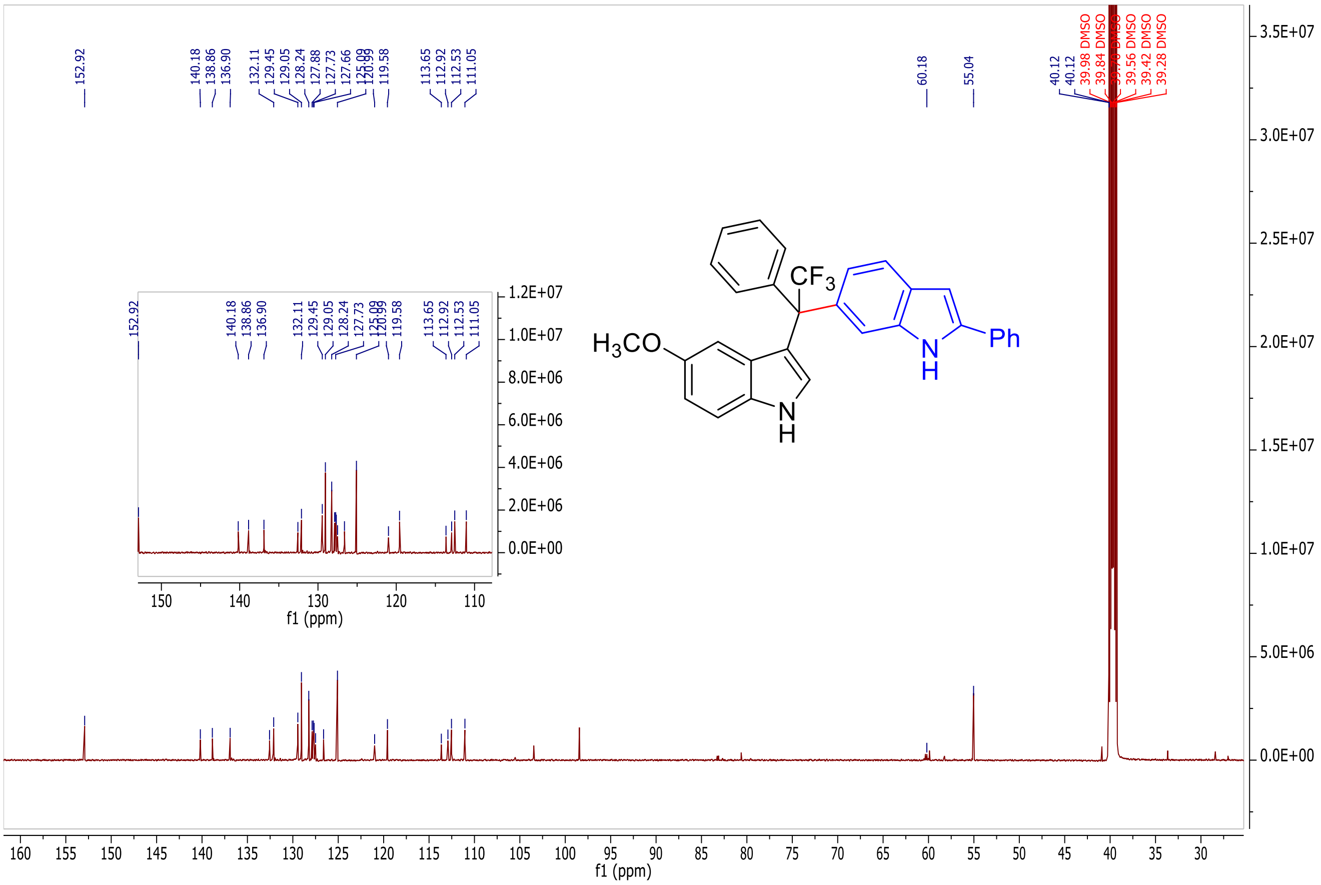

Figure S24. ^{1}H (600 MHz) and ^{13}C (151 MHz) Spectra of 5-methoxy-3-(2,2,2-trifluoro-1-phenyl-1-(2-phenyl-1*H*-indol-6-yl)ethyl)-1H-indole (**10**)

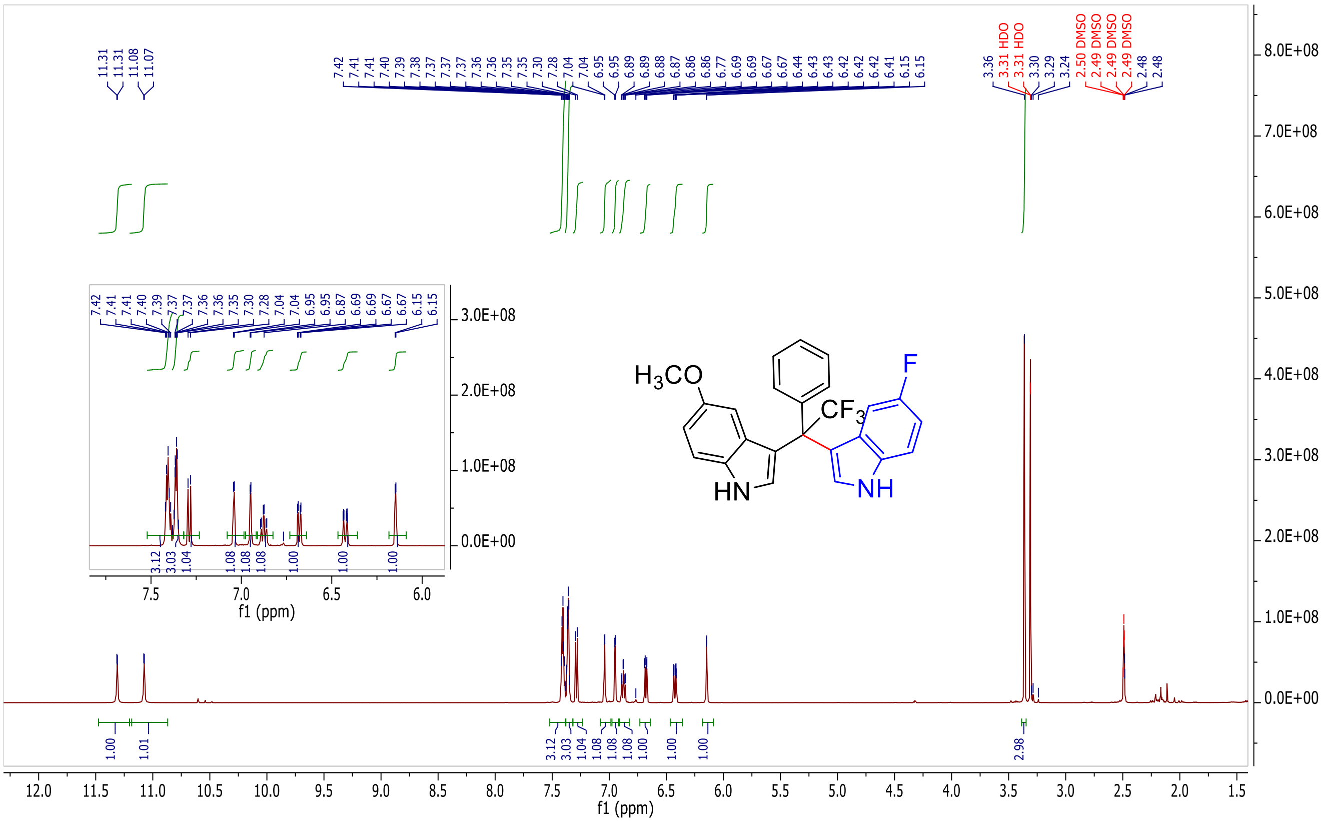

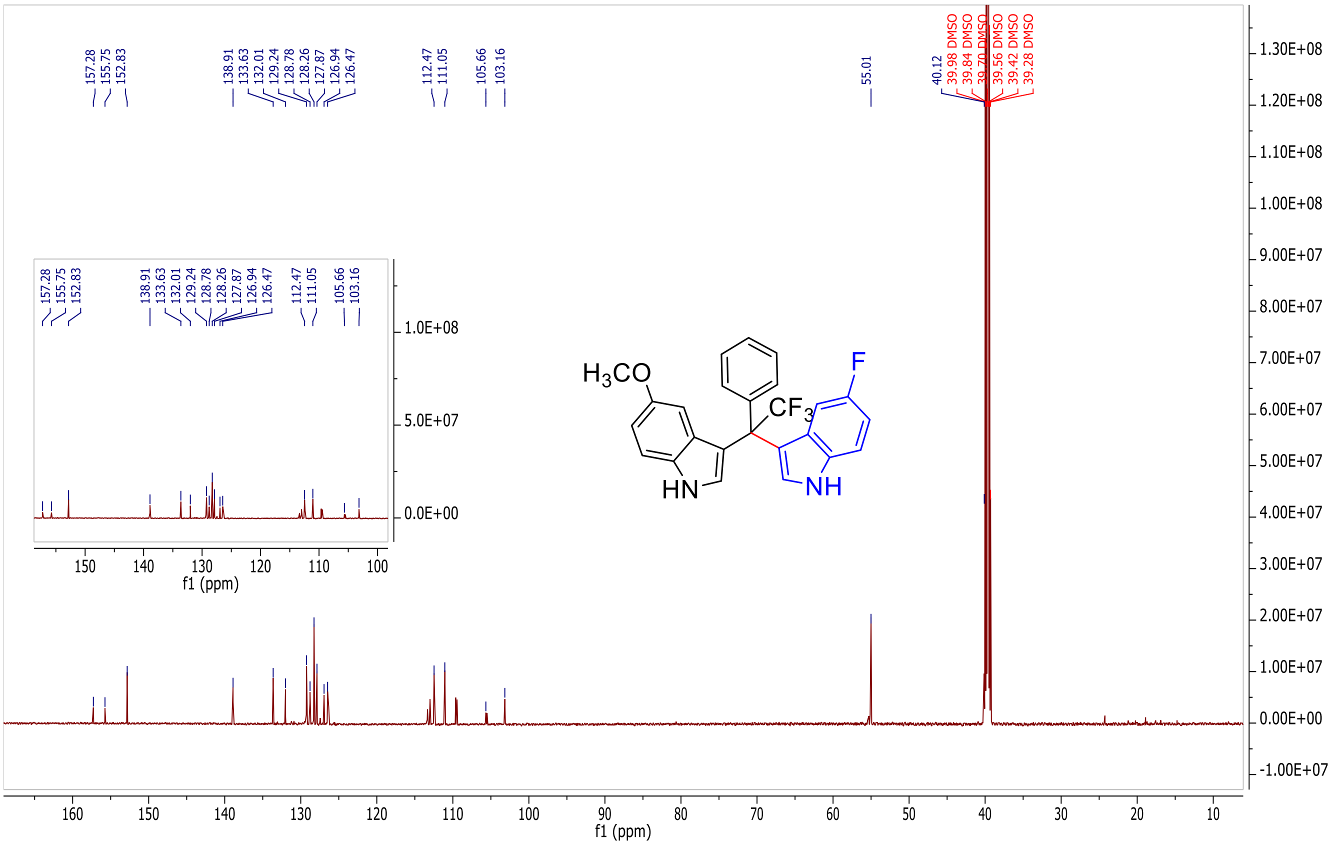

Figure S25. ^{1}H (600 MHz) and ^{13}C (151 MHz) Spectra of 5-fluoro-3-(2,2,2-trifluoro-1-(5-methoxy-1*H*-indol-3-yl)-1-phenylethyl)-1*H*-indole (**11**)

References

1. Bandini, M.; Sinisi, R. *Org. Lett.* **2009**, *11*, 2093-2096

2. G. M. Sheldrick, SHELXT – Integrated space-group and crystal-structure determination. *Acta. Cryst. A*. **2015**, *7*, 3-8.

3. G. M. Sheldrick, Crystal structure refinement with SHELXL. *Acta. Cryst. C.* **2015**, *71*, 3-8.
